# PseUI: Pseudouridine sites identification based on RNA sequence information

**DOI:** 10.1186/s12859-018-2321-0

**Published:** 2018-08-29

**Authors:** Jingjing He, Ting Fang, Zizheng Zhang, Bei Huang, Xiaolei Zhu, Yi Xiong

**Affiliations:** 1 School of Life Sciences, Anhui University, Hefei, 230601 Anhui China; 2 School of Life Sciences and Biotechnology, Shanghai Jiao Tong University, Shanghai, 200240 China

**Keywords:** Pseudouridine site, Position specific nucleotide propensity, Nucleotide composition

## Abstract

**Background:**

Pseudouridylation is the most prevalent type of posttranscriptional modification in various stable RNAs of all organisms, which significantly affects many cellular processes that are regulated by RNA. Thus, accurate identification of pseudouridine (Ψ) sites in RNA will be of great benefit for understanding these cellular processes. Due to the low efficiency and high cost of current available experimental methods, it is highly desirable to develop computational methods for accurately and efficiently detecting Ψ sites in RNA sequences. However, the predictive accuracy of existing computational methods is not satisfactory and still needs improvement.

**Results:**

In this study, we developed a new model, PseUI, for Ψ sites identification in three species, which are *H. sapiens*, S. cerevisiae, and *M. musculus*. Firstly, five different kinds of features including nucleotide composition (NC), dinucleotide composition (DC), pseudo dinucleotide composition (pseDNC), position-specific nucleotide propensity (PSNP), and position-specific dinucleotide propensity (PSDP) were generated based on RNA segments. Then, a sequential forward feature selection strategy was used to gain an effective feature subset with a compact representation but discriminative prediction power. Based on the selected feature subsets, we built our model by using a support vector machine (SVM). Finally, the generalization of our model was validated by both the jackknife test and independent validation tests on the benchmark datasets. The experimental results showed that our model is more accurate and stable than the previously published models. We have also provided a user-friendly web server for our model at http://zhulab.ahu.edu.cn/PseUI, and a brief instruction for the web server is provided in this paper. By using this instruction, the academic users can conveniently get their desired results without complicated calculations.

**Conclusion:**

In this study, we proposed a new predictor, PseUI, to detect Ψ sites in RNA sequences. It is shown that our model outperformed the existing state-of-art models. It is expected that our model, PseUI, will become a useful tool for accurate identification of RNA Ψ sites.

**Electronic supplementary material:**

The online version of this article (10.1186/s12859-018-2321-0) contains supplementary material, which is available to authorized users.

## Background

Pseudouridylation, which occurs at the uridine site and is catalyzed by pseudouridine synthase (PUS), has been observed in various RNAs of all organisms [1–4]. As the most abundant posttranscriptional modification, pseudouridylation plays an important role in the structure, function and metabolism of RNAs [5–9]. Therefore, it is crucial to identify pseudouridylation information for revealing the biological principles.

Although some experimental techniques for identifying Ψ sites have been developed, they are both time-consuming and costly [10–13]. Facing the exponential-increasing of RNA sequences in the post-genomic era, it is urgent to have an accurate, efficient and low-cost method to identify Ψ sites on RNA segments. Former studies suggest that computational methods or statistical learning methods are promising candidates because of their low cost and reasonable efficiency [14, 15].

Unfortunately, to the best of our knowledge, only two computational methods have been developed to predict Ψ sites in RNAs. Li et al. [15] built a model called PPUS to predict the PUS-specific Ψ sites in *H. sapiens* and *S. cerevisiae*. This model employed support vector machine (SVM) as the classifier and used the nucleotides around Ψ as features. Besides this PPUS model, Chen et al. [14] developed another model called iRNA-PseU to identify Ψ sites in *H. sapiens*, S. cerevisiae, and *M. musculus*. This model was built by incorporating the chemical properties of nucleotides and their occurrence frequency density distributions into the general form of pseudo nucleotide composition (pseKNC) [14]. Despite the promising results offered by these two computational methods, it is suggested that the performance of computational methods can be further improved by introducing other effective features such as position-specific nucleotide propensity and position-specific dinucleotide propensity [16].

In this study, we have developed a new model, PseUI, for Ψ sites identification from RNA sequences in *H. sapiens*, S. cerevisiae, and *M. musculus*. Based on the RNA sequence segment, we first generated five different kinds of features including nucleotide composition (NC), dinucleotide composition (DC), pseudo dinucleotide composition (pseDNC), position-specific nucleotide propensity (PSNP), and position-specific dinucleotide propensity (PSDP). Then, we selected a relevant feature combination by using a sequential forward feature selection strategy [17, 18]. Based on the selected features, our model was built by using a support vector machine (SVM). Finally, the prediction results provided by our models for the three species, *H. sapiens*, S. cerevisiae, and *M. musculus*, were compared with iRNA-PseU’s results by using both jackknife tests and independent validation tests on the benchmark datasets, and it is convincing from the result of comparison that our model PseUI can offer more accurate identification of Ψ sites than iRNA-PseU.

To develop a really useful feature-based analysis method for a biological system as reported in a series of recent studies [19–23], one should observe the 5-step rule [24]: (i) construct or select a valid benchmark dataset to train and test the predictor; (ii) formulate the biological sequence samples with an effective mathematical expression that can truly reflect their intrinsic correlation with the target to be predicted; (iii) develop a powerful algorithm (or engine) to operate the prediction; (iv) perform cross-validation and independent tests properly to objectively evaluate the anticipated accuracy of the predictor; and (v) establish a user-friendly web-server for the predictor that is accessible to the public. Below, we are to describe how to deal with these steps one-by- one.

## Methods

### Benchmark datasets

Three benchmark datasets, H_990, S_628, and M_944, were used for training in this study, where H, S, and M represent for *H. sapiens*, S. cerevisiae, and *M. musculus*, respectively, and 990, 628, 944 are the number of examples in each dataset. These three datasets are the same as that were used in Chen et al.’s work [14]. In their work, they downloaded RNA sequences with experimentally validated Ψ sites of *H. sapiens*, M. musculus and *S. cerevisiae* from RMBase [25]. In addition, they collected the RNA segments with uridine at the center but not experimentally conformed as Ψ sites from genomes as negative samples. More details about how to construct these datasets can be found in the reference [14].

The positive subset of H_990, S_628, and M_944 contains 495, 314, and 472 RNA segments, respectively, and each of these RNA segments has a uridine at the center position that can be pseudouridylated. The negative subset is composed of 495, 314, and 472 RNA segments, respectively, and each of these RNA segments has a uridine at the center position that cannot be pseudouridylated.

We can formulate each RNA segment, denoted as R_ξ_(U), in these datasets as follow:

1 $$ {R}_{\xi }(U)={N}_{-\xi }{N}_{-\left(\xi -1\right)}\cdots {N}_{-1}U{N}_1\cdots {N}_{+\left(\xi -1\right)}{N}_{\xi } $$

where the center U represents ‘uridine’, N_-ξ_ represents the ξ-th upstream nucleotide from the central uridine and N_+ξ_ represents the ξ-th downstream nucleotide.

The RNA samples in both of H_990 and M_944 are all composed of 21 nucleotides, while those in S_628 are composed of 31 nucleotides. Namely, the value of ξ is 10 and the RNA segment length is 2 × 10 + 1 for the datasets H_900 and M_944. The value of ξ is 15 and the RNA segment length is 2 × 15 + 1 for the dataset S_628.

Corresponding to the training datasets, Chen et al. [14] provided two independent testing datasets for *H. sapiens* and *S. cerevisiae*, i.e. H_200 and S_200, but not for *M. musculus*. The detailed sequence information for all the aforementioned datasets is given in Table 1; and the sequences of the five datasets can be found in Additional files 1, 2, 3, 4 and 5.

**Table 1:** The information of training datasets and independent testing datasets

Species	The name of training/testing datasets^a^	The length of the RNA sequences (bp)	The number of positive samples	The number of the negative samples
*H. sapiens*	H_990	21	495	495
	H_200	21	100	100
*S. cerevisiae*	S_628	31	314	314
	S_200	31	100	100
*M. musculus*	M_944	21	472	472
	–	–	–	–

^a^H_900, S_628, M_944 are the training datasets for H. sapiens, S. cerevisiae, M. musculus, respectively; H_200 and S_200 are the independent testing datasets for H. sapiens and S. cerevisiae, respectively

### Feature representation of the RNA samples

One of the key problems in designing a predictor based on machine learning is how to encode an RNA sequence as a feature vector containing highly discriminative information. With the explosive growth of biological sequences in the post-genomic era, one of the most important but also most difficult problems in computational biology is how to represent a biological sequence with a discrete model or a vector, yet still keep considerable sequence-order information or key pattern characteristic. This is because all the existing machine-learning algorithms can only handle vectors with equal lengths for all sequence samples, as elucidated in a comprehensive review [26]. However, a vector defined in a discrete model may completely lose all the sequence-pattern information. To avoid completely losing the sequence-pattern information for proteins, the pseudo amino acid composition [27] or PseAAC [28] was proposed. Encouraged by the success of using PseAAC to represent protein/peptide sequences, the concept of PseKNC (Pseudo K-tuple Nucleotide Composition) [29] was developed for generating various feature vectors to represent DNA/RNA sequences. Particularly, recently a very powerful web-server called Pse-in-One [30] have been established that can be used to generate any desired feature vectors for protein/peptide and DNA/RNA sequences according to the need of users’ studies. In the current study, five types of features, nucleotide composition (NC) feature, dinucleotide composition (DC) feature, pseudo dinucleotide composition (pseDNC) feature, position-specific nucleotide propensity (PSNP) feature, and position-specific dinucleotide propensity (PSDP) feature, were proposed to encode the RNA segments for identifying pseudouridine sites in RNA. Three of them, NC, DC, and pseDNC, can also be generated by Pse-in-One server [30].

#### Nucleotide composition (NC) and dinucleotide composition (DC) feature

Nucleotide composition, a classic method for the characterization of nucleotide sequences, is widely used in previous studies [31–33]. Theoretically, a k-mer nucleotide composition for an RNA sequence is a 4^*k*^dimensional vector which is consisted of the frequency of each k-mer types. Thus, we can obtain 4 types of nucleotide frequencies and 16 types of dinucleotide frequencies when k is equal to 1 and 2, respectively. We called these two features as NC and DC, respectively, and a 4-dimensional NC feature vector and a 16-dimensional DC feature vector were generated for an RNA segment.

#### Pseudo dinucleotide composition (pseDNC) feature

The pseudo oligonucleotide composition, or pseudo K-tuple nucleotide composition (PseKNC) [34–37], can be used to represent an RNA sequence with a discrete model or vector. This type of pseudo composition can still keep considerable sequence order information, particularly the global or long-range sequence order information, via the physicochemical properties of its constituent oligonucleotides [38]. In this study, we choose the value of K to be 2, namely, using pseudo dinucleotide composition (pseDNC) feature to represent the information of RNA sequences. Three physicochemical properties, free energy, hydrophilicity, and stacking energy, were used to generate features of pseudo dinucleotide composition (pseDNC), which are listed in Table 2.

**Table 2:** Three types of physicochemical properties of dinucleotides in RNA

Dinucleotide	Free energy	Hydrophilicity	Stacking energy
GG	−3.260	0.170	−11.100
GA	− 2.350	0.100	− 14.200
GC	−3.420	0.260	−16.900
GU	−2.240	0.270	−13.800
AG	−2.080	0.080	−14.000
AA	−0.930	0.040	−13.700
AC	−2.240	0.140	−13.800
AU	−1.100	0.140	−15.400
CG	−2.360	0.350	−15.600
CA	−2.110	0.210	−14.400
CC	−3.260	0.490	−11.100
CU	−2.080	0.520	−14.000
UG	−2.110	0.340	−14.400
UA	−1.330	0.210	−16.000
UC	−2.350	0.480	−14.200
UU	−0.930	0.440	−13.700

More details about the pseudo dinucleotide composition (pseDNC) feature refer to [38]

#### Position-specific nucleotide propensity (PSNP) and position-specific dinucleotide propensity (PSDP) feature

While position-specific amino acid preferences have been widely used in bioinformatics to predict functional site in biological sequences [39–42], the position-specific nucleotide preferences were first introduced in Li et al.’s paper [16], which were obtained by calculating the differences of the frequency of nucleotides in specific locations between positive and negative RNA segments.

For position-specific nucleotide propensity (PSNP) feature, according to the equation (1), the RNA segment can be reformulated as:

2 $$ {R}_{\xi }={N}_1{N}_2\dots {N}_{2\xi +1} $$

where N_j_(j=1,2,...,2ξ+1) represents the nucleotide at the j-th position of the RNA segment, and can be any one of the 4 nucleotides, i.e., *N*_*j*_ ∈ {*A*, *C*, *G*, *U*}.

First, we calculated the frequency of occurrence at the j-th position for the 4 types of nucleotides from both the positive and negative samples, respectively. Then, we combined the 4-dimensional positive vectors and the 4-dimendional negative vectors individually. In this way, we obtained two 4× (2ξ + 1) position-specific occurrence frequency matrixes, i.e., *Z*^+^ and *Z*^−^, where *Z*^+^ was obtained from all the positive samples, and *Z*^−^ was obtained from all the negative samples. Next, we defined the position-specific nucleotide propensity (PSNP) matrixes, denoted as *Z*_*PSNP*_, as below:

3 $$ {Z}_{PSNP}={Z}^{+}-{Z}^{-} $$

As for position-specific dinucleotide propensity (PSDP) feature, according to equation (2), the RNA segment can be rewritten in a dinucleotide form:

4 $$ {R}_{\xi }={N}_1{N}_2\dots {N}_{2\xi +1}={D}_1{D}_2\dots {D}_{2\xi } $$

where *D*_*j*_ = *N*_*j*_*N*_*j* + 1_(*j* = 1, 2, …, 2*ξ*) represents the dinucleotide at the j-th position of the RNA segment, and can be any of 16 types of dinucleotides, i.e., *D*_*j*_ ∈ {AA, AC, AG, …, UU}.

Similarly, following the principle we used to generate the *Z*_*PSNP*_ matrix, we can get the 16 × 2ξ position-specific dinucleotide propensity (PSDP) matrix. Both of the PSNP matrix and PSDP matrix can then be used to encode the new samples.

For the features encoded by PSNP and PSDP, we should pay particular attention to the fact that the propensity matrices (*Z*_*PSNP*_/*Z*_*PSDP*_) were only generated from the training samples without the one validation sample when evaluating the model using the jackknife test.

**Fig. 1:**
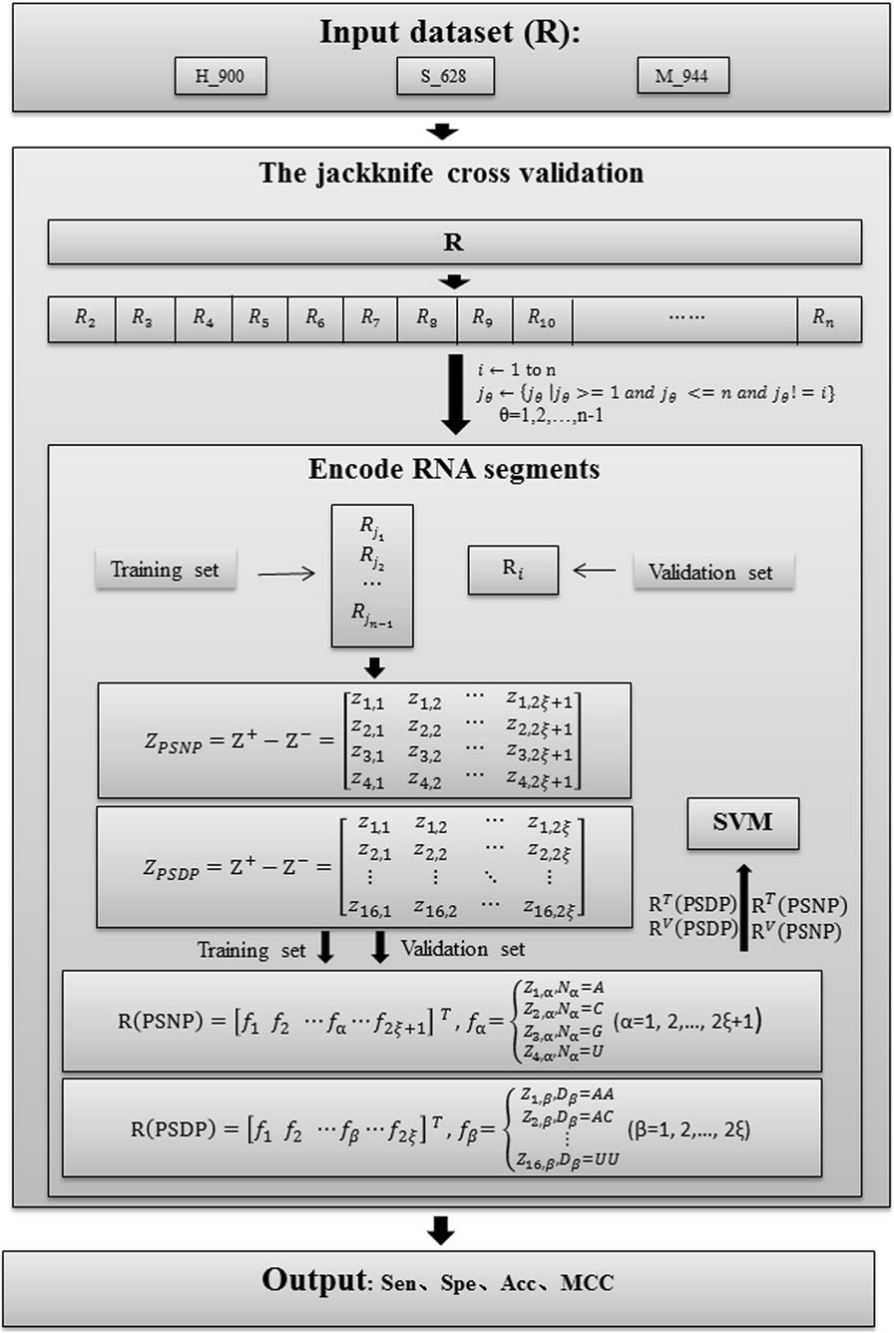
Flow charts of the jackknife cross validation for features encoded by PSNP or PSDP

Figure 1 clearly described the jackknife cross validation for features encoded by PSNP/PSDP. The validation process has four steps: (1) Input the dataset (R), e.g., H_990, S_628, or M_944, which is assumed to have n samples. (2) Divide the dataset (R) into n subsets and each subset will contain only one sample. (3) One subset is selected as the validation set, and the rest are used as the training set. The samples of the training set will be used to calculate the frequency of nucleotides at specific locations, and the position specific propensity matrices (Z_PSNP_/Z_PSDP_) will be obtained and then used to encode the RNA segments in the training set and the validation set. In such way, the feature matrices *R*^*T*^(PSNP/PSDP) and *R*^*V*^(PSNP/PSDP) can be obtained to represent the statistical information extracted from the training set and the validation set, respectively. A model will be then built by SVM based on the training set, and evaluated on the validation set. The whole process will be repeated for n times and each time a different sample will be selected as the validation set. (4) Count the results from the previous steps and calculate the evaluation parameter, i.e., Sen, Spe, Acc, and MCC, which are described in “Evaluation parameter” section.

### Model construction

#### Support vector machine

As a popular statistical learning method, SVM has been extensively used to build bioinformatics models [43–52]. Both of the PPUS and iRNA-PseU models [14, 15] mentioned in the background section were built by using SVM due to its high efficiency and robust output. In this study, we used the Matlab function FITCSVM to build our models. Different kernel functions can be used in SVM training, and we selected the radial basis function in this study. Two parameters c and g were referred for the radial basis function, which were called box constraint and kernel scale in FITCSVM, respectively. Here, we optimized these two parameters based on the jackknife test using a grid search.

In statistical analysis fields, three different validation methods have mostly been used to evaluate the performance of a machine learning model: independent dataset test, subsampling (or K-fold cross-validation) test, and jackknife test [53]. The jackknife test has already proved its effectiveness in many aspects [54, 55]. It is not affected by the random partition of the samples, and the final result is unique. In addition, the training set used by the jackknife test is only one sample less than the initial training set. Therefore, in most cases, the actual model evaluated by the jackknife test is very close to the expected model, which will offer more accurate results. Based on all these advantages, the jackknife test was used to evaluate the performance of our models.

#### Evaluation parameters

In recent studies, four evaluation parameters, Accuracy (Acc), Sensitivity (Sen), Specificity (Spe), and the Matthews correlation coefficient (MCC) have been frequently used to measure the predictor’s quality [46, 56]. The original formulas of the four parameters, particularly the MCC, are lacking intuitiveness and not easy to understand for most biologists. To make the most readers easy to understand, we here introduced the Chou’s intuitive formulas of the four parameters, as elaborated by the four sub-equations in Eq. 19 of [57], or the four sub-equations in Eq. 14 of [58]. Particularly, the advantages of Chou’s intuitive metrics have been analyzed and concurred by a series of studies published very recently [19, 20, 22, 59, 60]. The Chou’s intuitive metrics are formulated as below:

5 $$ \Big\{{\displaystyle \begin{array}{l}\mathrm{Sen}=1-\frac{N_{-}^{+}}{N^{+}},0\mbox{\fontencoding{T1}\fontfamily{cmr}\selectfont\char"13} Sen\mbox{\fontencoding{T1}\fontfamily{cmr}\selectfont\char"13} 1\\ {}\ \mathrm{Spe}=1-\frac{N_{+}^{-}}{N^{-}},0\mbox{\fontencoding{T1}\fontfamily{cmr}\selectfont\char"13} Spe\mbox{\fontencoding{T1}\fontfamily{cmr}\selectfont\char"13} 1\\ {}\ \mathrm{Acc}=1-\frac{N_{-}^{+}\kern0.5em +\kern0.5em {N}_{+}^{-}}{N^{+}\kern0.5em +\kern0.5em {N}^{-}},0\mbox{\fontencoding{T1}\fontfamily{cmr}\selectfont\char"13} Acc\mbox{\fontencoding{T1}\fontfamily{cmr}\selectfont\char"13} 1\\ {}\mathrm{MCC}=\frac{\begin{array}{ccc}1& -& \left(\begin{array}{ccc}\frac{N_{-}^{+}}{N^{+}}& +& \frac{N_{+}^{-}}{N^{-}}\end{array}\right)\end{array}}{\sqrt{\begin{array}{cc}\left(\begin{array}{ccc}1& +& \frac{\begin{array}{ccc}{N}_{+}^{-}& -& {N}_{-}^{+}\end{array}}{N^{+}}\end{array}\right)& \left(\begin{array}{ccc}1& +& \frac{\begin{array}{ccc}{N}_{-}^{+}& -& {N}_{+}^{-}\end{array}}{N^{-}}\end{array}\right)\end{array}}},-1\mbox{\fontencoding{T1}\fontfamily{cmr}\selectfont\char"13} MCC\mbox{\fontencoding{T1}\fontfamily{cmr}\selectfont\char"13} 1\end{array}} $$

Where *N*^+^ represents the total number of positive RNA samples; *N*^−^ represents the total number of negative RNA samples; ${N}_{-}^{+}$ represents the number of positive RNA samples that are incorrectly predicted as negative RNA samples; ${N}_{+}^{-}$ represents the number of negative RNA samples that are incorrectly predicted as positive RNA samples. In addition, it should be noted that the set of metrics in eq. (5) is only valid for the single-label systems (in which each sample only belongs to one class). For the multi-label systems (in which a sample might belong to several classes), whose existence has become more frequent in system biology [61] and system medicine [20] and biomedicine [60], a completely different set of metrics as defined in [62] is needed.

### Feature selection

In this study, we generated five types of features which composed a high dimensional feature vector for each sample. In order to obtain a more compact and effective feature subset, we conducted a sequential forward feature selection (SFS) [17, 18] process on the original features, which is described as follows:

In the first round, the performance metrics of each of the five types of features were calculated based on the jackknife test using a specific prediction engine, respectively. According to Acc or MCC, the best type of feature was selected to enter the next round of calculation. In the second round, the remaining four types of features were added to the type of feature selected by the first round. Similarly, according to Acc or MCC, the best combination of features was selected to enter the next round of calculation. This process continued to run until the Acc or MCC converged. The subset obtained with the highest Acc or MCC value will be regarded as the optimal feature subset.

## Results and discussion

### Performance of single type of feature

In this section, we evaluated the performance of each type of features using SVM over the rigorous jackknife test, and the feature PSNP was found to be particularly excellent for identifying Ψ sites. The performance of each evaluation index for the three species, i.e., *H. sapiens*, S. cerevisiae, and *M. musculus*, were listed in Tables 3, 4, and 5, respectively.

**Table 3:** The results of feature selection for H_990

Feature subset	Sen (%)	Spe (%)	Acc (%)	MCC	Kernel scale	Box constraint
NC	62.83	51.31	57.07	0.1424	0.5	4
DC	46.87	74.95	60.91	0.2273	2	256
pseDNC	44.24	76.57	60.40	0.2199	4	1024
PSNP	66.06	60.61	63.33	0.2671	8	512
PSDP	55.15	57.17	56.16	0.1233	0.5	1024
PSNP+NC	65.05	61.21	63.13	0.2628	1	4
*PSNP + DC*	*64.85*	*63.64*	*64.24*	*0.2849*	*2*	*8*
PSNP+pseDNC	64.44	62.42	63.43	0.2687	1	8
PSNP+PSDP	66.26	59.39	62.83	0.2572	8	1024
PSNP+DC + NC	64.85	63.43	64.14	0.2829	8	128
PSNP+DC + pseDNC	63.03	63.23	63.13	0.2626	4	32
PSNP+DC + PSDP	64.24	63.43	63.84	0.2768	1	2

The feature combination with the maximum MCC was italicized in the table

**Table 4:** The results of feature selection for S_628

Feature subset	Sen (%)	Spe (%)	Acc (%)	MCC	Kernel scale	Box constraint
NC	71.97	45.22	58.60	0.1785	1	8
DC	64.33	59.87	62.10	0.2423	0.25	1
pseDNC	58.92	62.42	60.67	0.2135	0.25	0.5
PSNP	50.96	72.93	61.94	0.2448	1	0.125
PSDP	49.36	73.57	61.46	0.2363	0.25	0.03125
DC + NC	59.55	61.78	60.67	0.2134	4	512
DC + pseDNC	62.42	60.51	61.46	0.2293	1	1024
DC + PSNP	63.69	65.29	64.49	0.2898	0.5	16
DC + PSDP	60.51	66.88	63.69	0.2744	0.125	2
DC + PSNP+NC	61.78	65.61	63.69	0.2741	0.25	1
*DC + PSNP + pseDNC*	*64.97*	*66.88*	*65.92*	*0.3185*	*0.25*	*2*
DC + PSNP+PSDP	63.38	67.20	65.29	0.3060	0.25	2
DC + PSNP+pseDNC+NC	61.78	65.92	63.85	0.2773	0.25	2
DC + PSNP+pseDNC+PSDP	62.74	67.52	65.13	0.3029	0.25	4

The feature combination with the maximum MCC was italicized in the table

**Table 5:** The results of feature selection for M_944

Feature subset	Sen (%)	Spe (%)	Acc (%)	MCC	Kernel scale	Box constraint
NC	56.99	53.18	55.08	0.2233	2	2
DC	61.86	52.75	57.31	0.1468	4	1024
pseDNC	72.46	44.28	58.37	0.1744	4	128
PSNP	73.31	66.31	69.81	0.3972	0.5	1
PSDP	68.22	60.38	64.30	0.2869	1	256
PSNP+NC	69.70	70.34	70.02	0.4004	0.25	0.125
*PSNP + DC*	*74.58*	*66.31*	*70.44*	*0.4103*	*1*	*2*
PSNP+pseDNC	74.15	66.53	70.34	0.4080	0.5	1
PSNP+PSDP	68.64	70.97	69.81	0.3963	0.125	0.5
PSNP+DC + NC	74.15	66.10	70.13	0.4039	0.5	0.25
PSNP+DC + pseDNC	73.09	67.80	70.44	0.4095	0.5	0.5
PSNP+DC + PSDP	74.58	66.31	70.44	0.4103	0.5	0.25

The feature combination with the maximum MCC was italicized in the table

**Fig. 2:**
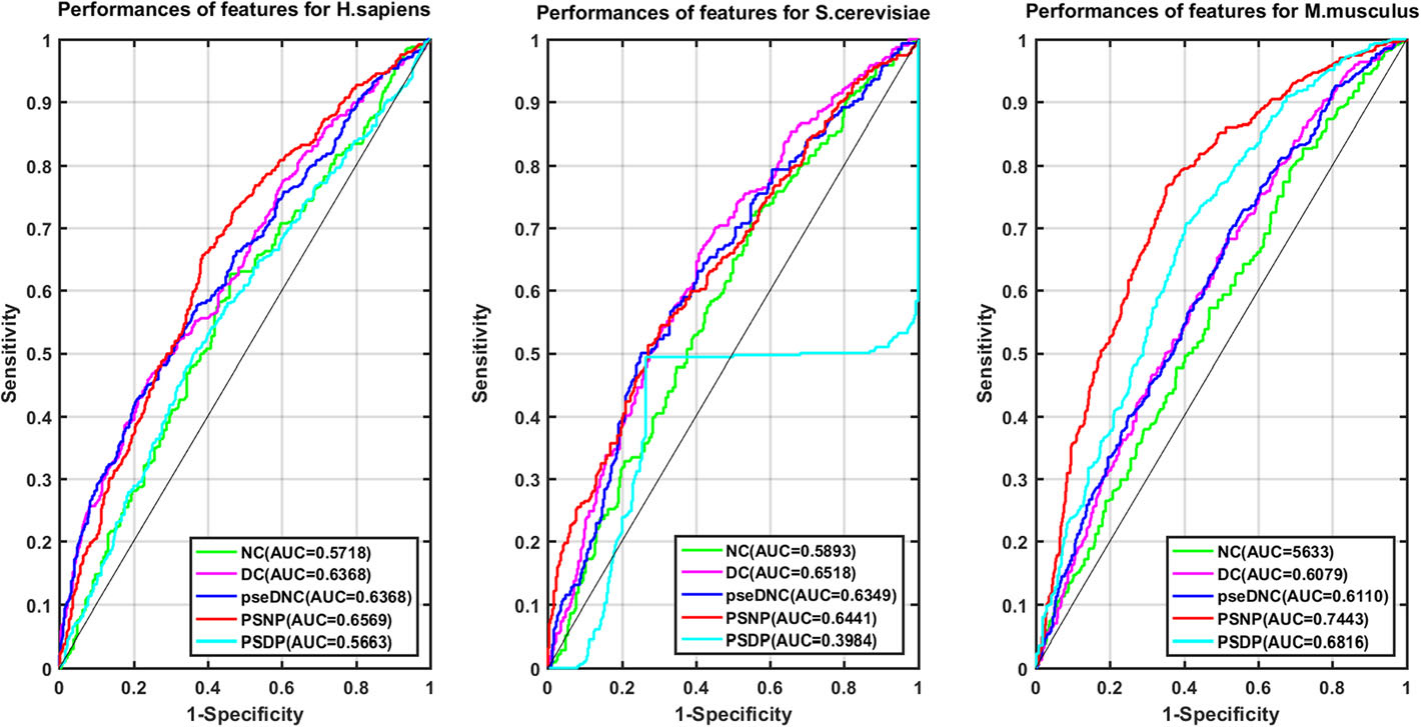
The ROC curves that show the performances of the five type of features for H.sapiens, S.cerevisiae, and M.musculus, respectively

In addition, the receiver operating characteristic (ROC) curves [63] were employed to show the results more clearly. On the ROC curve, the diagonal line from point (0, 0) to (1, 1) corresponds to the random guessing model, and the point (0, 1) corresponds to the ideal model with no positive example wrongly predicted. When comparing models, if the ROC curve of one model is completely enveloped by the curve of the other model, it can be asserted that the latter model is superior to the former in performance. However, it is difficult to judge when the ROC curves of two models cross. In this situation, the area under the ROC curve (AUC) will be used as the more reasonable criteria for comparing model performance, and the lager AUC indicates better performance. The ROC curves of the five types of feature for each species were plotted in Fig. 2, together with the AUC values.

As shown in Fig. 2, the AUC values of PSNP are 0.6569, 0.6441, and 0.7443 for *H. sapiens*, S. cerevisiae, and *M. musculus*, respectively. For *H. sapiens* and *M. musculus*, the AUC values of PSNP are much higher than those of the other four types of features. For *S. cerevisiae*, the AUC value of PSNP is only 0.0077 lower than the highest AUC value 0.6518 given by DC. Moreover, the accuracy was improved from 62.10 to 64.49% when PSNP was added in the second round of SFS for *S. cerevisiae*, which was shown in Table 4. These results all indicate that PSNP offered the best performance among these five types of features and the addition of PSNP provided a great possibility of improving the model performance, which may lay the foundation for our future works.

### Feature subsets selected by SFS

For the selection of feature subset with SFS described in the “Feature selection” section, we run three rounds of calculation for the datasets H_990 and M_944, respectively. Finally, the subset that made up of DC and PSNP features was chosen as the optimal feature subset. The results of each round for *H. sapiens* and *M. musculus* are shown in Tables 3 and 5, respectively. For both *H. sapiens* and *M. musculus*, the best models were built based on the feature subset PSNP+DC.

For the dataset S_628, four rounds of calculation were conducted, and the subset with a combination of DC, pseDNC, and PSNP, was selected as the optimal feature subset. The results of each round are listed in Table 4. The best model of *S. cerevisiae* is built based on the feature subset DC + PSNP+pseDNC.

### Comparison with existing methods

In this section, we compared our model PseUI with the latest model iRNA-PseU [14] by using two validation methods (i.e., the jackknife cross validation and independent tests) to confirm the predictability of our model.

Unfortunately, after a careful study of Chen et al.’s article [14], we found that some of the results reported by the authors were not reasonable. For example, the values of Sen (Sensitivity) and Spe (Specificity) for *S. cerevisiae* using the jackknife cross validation were 64.65 and 64.33% (see Table 6). However, according to the ROC curve in Chen et al.’s paper [14], the value of “1-Specificity” is estimated to be approximately 0.24, thus the “Specificity” value should be approximately 0.76, when “Sensitivity” is 0.6465. This “specificity” value (0.76) is significantly different from the aforementioned “specificity” value (64.33%). Besides this big discrepancy in “specificity” values, the optimized parameters g and c were not reported in the paper.

**Table 6:** A comparison of PseUI with iRNA-PseU and re-iRNA-PseU on three training datasets

Training datasets	Predictor	Sen (%)	Spe (%)	Acc (%)	MCC	AUC
H_990	iRNA-PseU^a^	61.01	59.80	60.40	0.21	0.64
	re-iRNA-PseU^b^	65.05	58.79	61.92	0.24	0.65
	PseUI^c^	64.85	63.64	64.24	0.28	0.68
S_628	iRNA-PseU^a^	64.65	64.33	64.49	0.29	0.81
	re-iRNA-PseU^b^	66.88	64.33	65.61	0.31	0.69
	PseUI^c^	62.10	71.02	66.56	0.33	0.69
M_944	iRNA-PseU^a^	73.31	64.83	69.07	0.38	0.75
	re-iRNA-PseU^b^	79.87	60.81	70.34	0.41	0.75
	PseUI^c^	74.58	66.31	70.44	0.41	0.77

^a^The predictor developed by Chen et al. [14]

^b^The predictor we re-implemented by the method proposed by Chen et al. [14]

^c^The predictor proposed in this paper

To have a more accurate comparison with Chen et al.’s method, we wrote our programs in strict accordance with the description of their paper to re-implement iRNA-PseU. The software LIBSVM-3.22 was used to train the SVM models. To obtain the best performance of the jackknife cross validation, we used a grid search to optimize the SVM parameter g from 2^− 15^ to 2^− 5^ and parameter c from 2^− 5^ to 2^15^ with a step of 2. Finally, the parameters g and c were set at 0.01562 and 2 for *H. sapiens*, 0.0003 and 32,768 for S. cerevisiae, and 0.00098 and 4 for *M. musculus*, respectively.

**Fig. 3:**
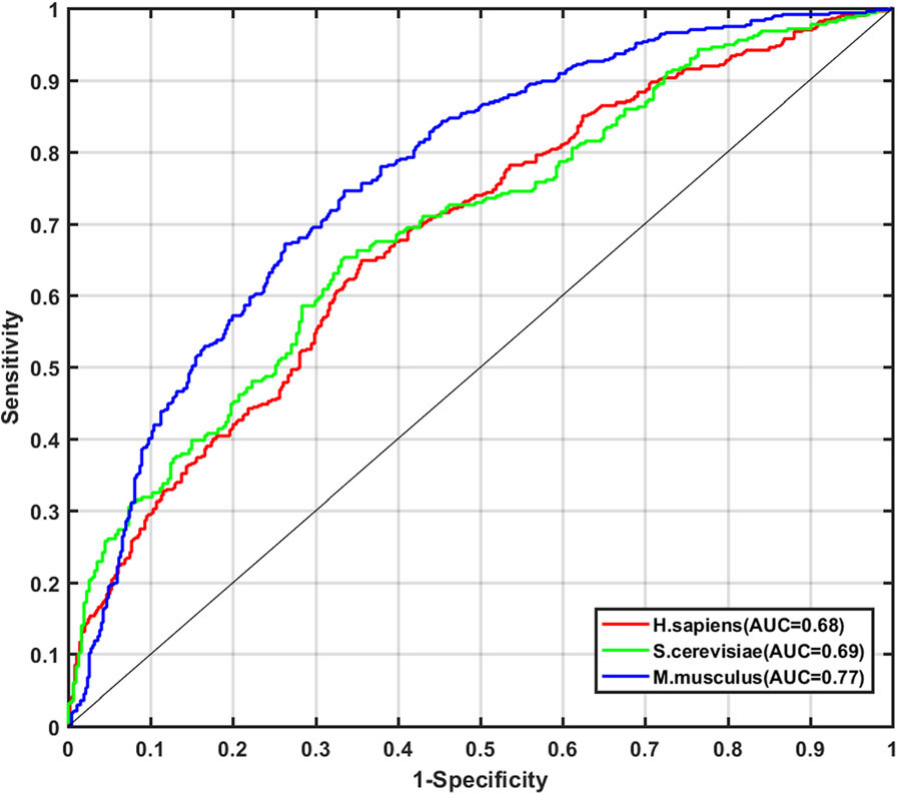
The ROC curves of the best models for H.sapiens, S.cerevisiae, and M.musculus, respectively

Then, we compared the proposed PseUI with the re-implemented iRNA-PseU (named re-iRNA-PseU) by using the jackknife cross validation. The comparison results for the three training datasets, i.e., H_990, S_628, and M_944, were listed in Table 6, and the ROC curves of PseUI were shown in Fig. 3. As shown in Table 6, both Acc and MCC obtained by PseUI are higher than those obtained by re-iRNA-PseU. For Acc, improvements of 2.32%, 0.95%, and 0.10% were observed for H_990, S_628, and M_944, respectively, and for MCC, improvements of 4 and 2% were observed for H_990 and S_628. In addition, as shown in Fig. 3, the AUC values of PseUI are 0.68 and 0.77, which are 0.03 and 0.02 higher than the corresponding AUC values of re-iRNA-PseU for *H. sapiens* and *M. musculus*, respectively. These findings confirmed that the PseUI outperformed the re-iRNA-PseU in both accuracy and stability for identifying Ψ sites. Note that the re-iRNA-PseU is superior to iRNA-PseU according to the evaluation metrics shown in Table 6.

Next, we compared our models PseUI with the re-iRNA-PseU on the independent datasets. In this study, independent datasets are only available for the species of H. sapiens and *S. cerevisiae* (i.e., H_200 and S_200), so the comparison was only conducted on these two datasets. The results were listed in Table 7.

**Table 7:** A comparison of PseUI with the re-iRNA-PseU on two independent datasets

Datasets	Predictor	Sen (%)	Spe (%)	Acc (%)	MCC
H_200	re-iRNA-PseU^a^	58.00	65.00	61.50	0.23
	PseUI^b^	63.00	68.00	65.50	0.31
S_200	re-iRNA-PseU^a^	63.00	57.00	60.00	0.20
	PseUI^b^	72.00	65.00	68.50	0.37

^a^The predictor we re-implemented by the method proposed by Chen et al. [14]

^b^The predictor proposed in this paper

As shown in Table 7, the predictive Accs of H_200 and S_200 are 65.50 and 68.50%, which are similar to the corresponding cross validation Accs on the training datasets. This means that our model is stable and has good generalization ability for predicting Ψ sites. When compared with re-iRNA-PseU, the proposed PseUI model showed improvements of 4 and 8.5% of the Accs values on the two independent test sets, respectively. As for MCC, PseUI outperformed re-iRNA-PseU with improvements of 0.08 and 0.17 for H_200 and S_200, respectively. All these results confirmed that our proposed model PseUI is superior to re-iRNA-PseU.

### Web implementation

As demonstrated in a series of recent publications [58, 61, 64–75], user-friendly and publicly accessible web-servers or source codes represent the future direction for developing practically more useful analysis methods and computational tools. Actually, many practically useful web-servers have significant impacts on medical science [26], driving medicinal chemistry into an unprecedented revolution [76]. For the convenience of academic users, we did the same and established a user-friendly and publicly accessible web server for PseUI, which is freely accessible at http://zhulab.ahu.edu.cn/PseUI. Users can easily get their desired results without complicated mathematic calculations. The final online PseUI method was trained on H_990, S_628, and M_944, which are composed of 21, 31, and 21 nucleotides, respectively. The detailed procedure to predict Ψ sites by using PseUI method is as follows:

Firstly, a query RNA sequence is submitted and the RNA sequence should be longer than 21 bp for *H.sapiens* and *M.musculus* or longer than 31 bp for S.cerevisiae in FASTA format. Secondly, PseUI identifies each uridine site in the query RNA sequence, and a corresponding 21-nt RNA segment for *H.sapiens* and *M.musculus* or 31-nt RNA segment for S.cerevisiae is constructed by placing a sliding window centered on the uridine site. Thirdly, according to the reconstructed RNA segment, the vector for the statistical information of the sequence is extracted by the features, and then submitted to the SVM classification engine for prediction. Finally, the users can get the result they desired. Please notice that the reconstructed RNA segment for unequal number of nucleotides around the target uridine is filled with its mirror image [47].

## Conclusion

In this study, we proposed a model, PseUI, for accurate and efficient identification of Ψ sites in RNA sequences. We compared our model PseUI with the latest Ψ site identification model iRNA-PseU [14] by using two different methods, jackknife cross validation and independent tests. The results showed that our model is more accurate and stable than iRNA-PseU. In addition, the performances of the five types of features used in this study were systematically evaluated and compared, and the feature of PSNP was found to show the best performance. To facilitate the use of our model, a web server was built at http://zhulab.ahu.edu.cn/PseUI, which allows the academic users to easily use our model to predict the Ψ sites in RNA sequences.

## Additional files

Additional file 1: The benchmark dataset H_990 for H.sapiens. The benchmark dataset H_990, S_628, and M_944 is formed by 495, 314 and 472 Ψ-site-containing sequences and 495, 314 and 472 false Ψ-site-containing sequences, respectively. Both H_200 and S_200 are formed by 100 Ψ-site-containing sequences and 100 false Ψ-site-containing sequences, and none of the samples included here occur in the corresponding benchmark datasets. Each of these samples for *H.sapiens* and *M.musculus* is 21-bp long with the uridine located at the center, and each of these samples for S.cerevisiae is 31-bp long with the uridine located at the center. None of the sequences included here has ≥60% pairwise sequence identity to any other in a same subset. (DOCX 56 kb)

Additional file 2: The benchmark dataset S_628 for S.cerevisiae. The benchmark dataset H_990, S_628, and M_944 is formed by 495, 314 and 472 Ψ-site-containing sequences and 495, 314 and 472 false Ψ-site-containing sequences, respectively. Both H_200 and S_200 are formed by 100 Ψ-site-containing sequences and 100 false Ψ-site-containing sequences, and none of the samples included here occur in the corresponding benchmark datasets. Each of these samples for *H.sapiens* and *M.musculus* is 21-bp long with the uridine located at the center, and each of these samples for S.cerevisiae is 31-bp long with the uridine located at the center. None of the sequences included here has ≥60% pairwise sequence identity to any other in a same subset. (DOCX 45 kb)

Additional file 3: The benchmark dataset M_944 for M.musculus. The benchmark dataset H_990, S_628, and M_944 is formed by 495, 314 and 472 Ψ-site-containing sequences and 495, 314 and 472 false Ψ-site-containing sequences, respectively. Both H_200 and S_200 are formed by 100 Ψ-site-containing sequences and 100 false Ψ-site-containing sequences, and none of the samples included here occur in the corresponding benchmark datasets. Each of these samples for *H.sapiens* and *M.musculus* is 21-bp long with the uridine located at the center, and each of these samples for S.cerevisiae is 31-bp long with the uridine located at the center. None of the sequences included here has ≥60% pairwise sequence identity to any other in a same subset. (DOCX 54 kb)

Additional file 4: The independent dataset H_200 for H.sapiens. The benchmark dataset H_990, S_628, and M_944 is formed by 495, 314 and 472 Ψ-site-containing sequences and 495, 314 and 472 false Ψ-site-containing sequences, respectively. Both H_200 and S_200 are formed by 100 Ψ-site-containing sequences and 100 false Ψ-site-containing sequences, and none of the samples included here occur in the corresponding benchmark datasets. Each of these samples for H.sapiens and M.musculus is 21-bp long with the uridine located at the center, and each of these samples for S.cerevisiae is 31-bp long with the uridine located at the center. None of the sequences included here has ≥60% pairwise sequence identity to any other in a same subset. (DOCX 26 kb)

Additional file 5: The independent dataset S_200 for S.cerevisiae. The benchmark dataset H_990, S_628, and M_944 is formed by 495, 314 and 472 Ψ-site-containing sequences and 495, 314 and 472 false Ψ-site-containing sequences, respectively. Both H_200 and S_200 are formed by 100 Ψ-site-containing sequences and 100 false Ψ-site-containing sequences, and none of the samples included here occur in the corresponding benchmark datasets. Each of these samples for *H.sapiens* and *M.musculus* is 21-bp long with the uridine located at the center, and each of these samples for S.cerevisiae is 31-bp long with the uridine located at the center. None of the sequences included here has ≥60% pairwise sequence identity to any other in a same subset. (DOCX 25 kb)

## Abbreviations

DC: Dinucleotide Composition
NC: Nucleotide Composition
PSDP: Position-Specific Dinucleotide Preferences
PSNP: Position-Specific Nucleotide Preferences
PUS: Pseudouridine Synthase
SFS: Sequential Forward Selection
SVM: Support Vector Machine
