# Supplementary material for: PseUI: Pseudouridine sites identification based on RNA sequence information
Source: BMC Bioinformatics. 2018 Aug 29;19:306. doi: 10.1186/s12859-018-2321-0 (PMC6114832; doi:10.1186/s12859-018-2321-0)
Supplement: Supplementary file 1 — The benchmark dataset H_990 for H.sapiens. The benchmark dataset H_990, S_628, and M_944 is formed by 495, 314 and 472 Ψ-site-containing sequences and 495, 314 and 472 false Ψ-site-containing sequences, respectively. Both H_200 and S_200 are formed by 100 Ψ-site-containing sequences and 100 false Ψ-site-containing sequences, and none of the samples included here occur in the corresponding benchmark datasets. Each of these samples for H.sapiens and M.musculus is 21-bp long with the uridine located at the center, and each of these samples for S.cerevisiae is 31-bp long with the uridine located at the center. None of the sequences included here has ≥60% pairwise sequence identity to any other in a same subset. (DOCX 56 kb) [file 12859_2018_2321_MOESM1_ESM.docx]

**The benchmark dataset H_990 for *H.sapiens*.** It is formed by 495 Ψ-site-containing sequences and 495 false Ψ-site-containing sequences. Each of these samples is 21-bp long with the uridine located at the center. None of the sequences included here has $\geq60\%$ pairwise sequence identity to any other in a same subset.

**I. 495 Ψ-site-containing sequences (positive samples)**

>P1

GAUAAAAGAGUUACUUUGAUA

>P2

AAAUAAGCUAUCGGGCCCAUA

>P3

CCCGAAAAUGUUGGUUAUAUC

>P4

UAACUUAGCAUUAACCUUUUA

>P5

UGGUGUCUGCUACCAUGAGUG

>P6

UGUGCUCGUGUAAGCCCGGGG

>P7

UCACGUCCUUUGCAGGUCACC

>P8

GCUGGAUUUGUAGCUUAUCUU

>P9

CCCUGGGCAGUAUAGAGACGU

>P10

GGGCUACCUCUUCAAGAGGGC

>P11

CGGCAGCCGCUCUCCACUCCC

>P12

GCUUUUAACCUCUUGUUCUGA

>P13

UGGGGAAGGAUUCCGUGCCUU

>P14

GCCCACUGCUUAGGGGUCACC

>P15

AGCACACCCCUUAGCGCUUGC

>P16

GAGGGAGCUGUAGGAAUAGUG

>P17

AGGAUGUAGUUAGAUUCUGGC

>P18

UGUGGAUGGUUACAGCCCAUC

>P19

GCUAGAAGGUUCGAUGCCUCU

>P20

UGGACUUAGAUUCCCUGAAGA

>P21

GUCAUCUAGAUUAUGGGGAAA

>P22

AUCGAGUGUUUCGACCGAGUU

>P23

UAUACUAAAAUUGGAACGAUA

>P24

ACACGCAAAUUCGUGAAGCGU

>P25

GGCAAGCUGUUGAUGAUUUCA

>P26

AAUAAGACAGUGCUAGACUCA

>P27

UGUAAUAUUUUAUCCCUGGAC

>P28

CGUUUGUACUUUGGGGAAAGG

>P29

UUUCGCCUUUUACUAAAGAUU

>P30

UUUGUGCCCUUCGUGCAGCUG

>P31

GGUUGGUGGUUCGAGCCCACC

>P32

GUUAGCGCGUUCGGCUGUUAA

>P33

CGGCCCGGGUUCGAUUCCCGG

>P34

CAUGAUUAAAUUCAGCCUAAA

>P35

GGCAGGAGACUGCAUGUCACC

>P36

GAGGCGUAGGUGAGGGUCGCG

>P37

AUCUCAUACUUACCUGGCAGG

>P38

UUACCAUUUUUUGUUUGCAAC

>P39

UUGCAGGGCUUUCGGCUGGAG

>P40

UAAAGGAUUGUAUCAAGUAGA

>P41

CCUGAUAUCCUACACAACAAA

>P42

ACCUGUUAUGUAGAGGAAAUA

>P43

GAGCUCUCCAUUCACAAACAU

>P44

CCUUGGGAGAUUGAUUUGCAC

>P45

GUAUGAUUUAUCUGGUUCAAG

>P46

GAAAAAGACAUAGUAUACCCU

>P47

UUGGACAAGUUGUUACUGACU

>P48

UAAAGUUAUAUACUCUAUGUU

>P49

AGACUAUACUUUCAGGGAUCA

>P50

CCUCUCAGGGUUUCACACGUA

>P51

CUUUUUCAUAUUCUAAUAAAU

>P52

GGAAAGUCCCUUACUCGGCCC

>P53

CUUCCCGCUGUAGAGCUGCUC

>P54

AAUCAGAUCUUUUCUUUCUUG

>P55

ACUCCGGCUCUAGCCUCCCGC

>P56

CAUGCAGAACUUUGCCUUCAG

>P57

UUGAGGAAGUUACAAAUAAGC

>P58

AAUUAAUUGGUUCUUUGCACU

>P59

UAAUCUGGUAUAUGGCAUGUG

>P60

AAAGGAUGGAUUAAACGAUGA

>P61

UGGUAUGUGUUUUCCAAACUG

>P62

CUUCUAUCGCUUCUCGGCCUU

>P63

GCUUUUUCUGUUCCACCUCUG

>P64

GGCACAGACUUUCUAUUUUUC

>P65

UUCCACUUUCUUUAGUGGCAU

>P66

UGCAAGCAACUCUGCUCUGUG

>P67

GGCUACCUACUUUCAUGAGUA

>P68

CACGAGGAGUUCAACCCUCCA

>P69

CCCAGCUCCAUCCGUGGCUCU

>P70

CUUCCCUCCUUUAGAACUCAU

>P71

AGUGACUUUUUAAAGAGACAC

>P72

UUUUUUUUUUUUCCCUAAGAG

>P73

AGAGCCCCAAUUCCUACUUCA

>P74

UUAAAUCAGUUCCAGCCUGCA

>P75

AUCACAGAUAUUCAGAUUCCU

>P76

UGUGCAGGCAUUUGACUCGCU

>P77

UUUGCUUGAUUUUGUAGAUGA

>P78

UCUUCCCCUGUGGUUCCAGCC

>P79

CUAAUGCUCUUUAGCUGUUAA

>P80

UAAUAAAAGUUUACAACUUGA

>P81

ACACCUGGGUUCCAAUCCCAG

>P82

GACCUUUACCUUGGGGGACAA

>P83

GGGCAGGCUCUUCAAGUGUGG

>P84

AACAACCGCUUUGAGGAGUAU

>P85

AUUCUUUUUCUAGGGAUGUAA

>P86

GUGCUCUUGCUGCAGUUCCCU

>P87

CCAUCACCCUUACCCGACAAC

>P88

GGCCAUGGAGUCCACAGCCAC

>P89

ACCCAGCUGCUUUUGAGUCCC

>P90

CCGAAACACGUAUUUUAAGCG

>P91

GUUCAAGCGAUUUUCCUGCUU

>P92

UCCCUGCCCUUUUAAUACAGG

>P93

ACCCUACCUGUAGGAACACAU

>P94

AUCUUUGGUAUGGUUGCCUUA

>P95

UCAUCAUUAUUAUUUUGGUUA

>P96

AGAUCAAGUGUAGUAUCUGUU

>P97

GGUGAGCCAAUAUCAUGCCAC

>P98

UUGUCAGAAUUUAGACUGAAA

>P99

UUUUGUGGAGUUUGACACCUU

>P100

AUGUGAUGGUUUUGGAUGGAG

>P101

ACAGUCAGUUUUAGGAACUAG

>P102

CCCAUGAUGAUUGGUGAGUUG

>P103

GGAGAUGAGUUUUAUUCUCAG

>P104

AAAACAGGACUCUGUGUCUCA

>P105

GCGGCCCUACUCUCUGACCCC

>P106

ACAGGGCAUCUUCUAUGCGAU

>P107

UUGCCUUCUUUUAAGAGAUGG

>P108

UAGGGCCACCUGUACCCCAUG

>P109

AGAAAAUACUUAAAGGGAUCA

>P110

AAUGCAGAACUUAAGAAGGUU

>P111

UUCAAGGAGUUUCAACAAUUU

>P112

CCUAUCAUUGUGAAGCAGAAU

>P113

GAUAACUGGCUUGUGGCGGCC

>P114

UUUUGGGUUUUAAGCAGGAGG

>P115

CGCUUGAUCUUGAUUUUCAGU

>P116

AACGUUCUAUUGUCAAACUCU

>P117

CAGUCAGUAGUUGGUCCUUUG

>P118

AGGAAAGCAUUCCUGACAUUC

>P119

ACCUCCACGGUAUAUGAGUGU

>P120

CCGGGACCAGUGGAGACUGUA

>P121

GGGAAAAGUUUUGGUUGCCCU

>P122

AUAAACCCUGUAGGCCAGGGU

>P123

GUGGUGUAACUGUGCCUUUUU

>P124

CAGAAAAAUUUGGCACCAGGC

>P125

CACAAAUAACUAGUAGUUUCC

>P126

AUUGCUCAUCUUUUCUCCCCA

>P127

ACAUUAUAUGUAAUGUUAGUA

>P128

CUACAAAGAUUUCACCAUCUA

>P129

GGAUGACAACUUUGCCUCAAU

>P130

UUGCUUGCAAUAUCCUAGAAU

>P131

AUUGCAGAGUUGAGUUAAGUU

>P132

GUACUCUGCGUUGUGGCCGCA

>P133

CCUGGUGGUCUAGUGGUUAGG

>P134

UUAUUUGUGUUGGGGGAAAUG

>P135

GGUCCCCGGUUCGAAACUGGG

>P136

GGUUAUCACGUUCGCCUCACA

>P137

GGGAGAUUUUUUUGUAGUUUU

>P138

CGGCUGCACUUUCGUCGCGUU

>P139

UGGUUCUGGUUAAACAGCUGU

>P140

UCUUAAGAUCUGGUUACGGUA

>P141

AUUUCCUUGUUGAGAACAGGA

>P142

AGAUACCAGCUUUUUUUUUUU

>P143

CUCCAGACCUUGUAUGAUUAC

>P144

CCGCCGCACGUACUAAGGAAG

>P145

GGUGCUUUUGUACCUAGGUUU

>P146

UGCUUAGGCCUAGCCAGUGAG

>P147

UGUGCCUGGCUUUGCUGCUCC

>P148

UCACUGUCCGUACAAACACAA

>P149

CAUGCUGAGCUUCUGUGGGAA

>P150

GAAUUGAAAUUGUUGGACAUC

>P151

CCAACUCCUAUACCUUACCAG

>P152

AGAAUGAGACUCUGUCUCAGA

>P153

CGAUACCUAUUUUCUAGUGCU

>P154

AGUGCAUAUUUACAAGAAGCA

>P155

AAAAUGUCACUUUGCCUGCUG

>P156

AAUGUAUCAUUAUCUUCAUUC

>P157

AAGACAGAGCUAUUUCAGAGG

>P158

CCACUGUCCCUACCUACUAUC

>P159

ACGUGGCCUUUUCUACCCUGG

>P160

UCCUCCCUUUUAUUACAACAU

>P161

UGAUCUUUUUUUAAAAAACAC

>P162

GACAUGCAGUUAAGUGAAUUU

>P163

GAUUGACCUUUUACAAGCCUU

>P164

UUCUUUUCCCUUUAUGUCACA

>P165

GUGAUCUGGUUUUAGCCCAGA

>P166

AAUGGAGUACUUUGAUGAAAA

>P167

CUAUACUGCAUUUGAAGUGUA

>P168

GCAUUAUAUUUAUUUUUCUUU

>P169

UCUUUCAGGUUUGACUCCUGG

>P170

UGGAGAAAAGUAGGAUGAACC

>P171

UACUGAAGGUUUUAGUCCUCA

>P172

AAAAAAAAACUAUGCCUAAAU

>P173

GCUGCAGAUAUCACCCAGGCA

>P174

ACGGCGGGAGUAACUAUGACU

>P175

AAUAUUUUAAUUACUUUUGCA

>P176

UUUUAUUUAUUUAUUUUUGAC

>P177

AACUGAAAGCUUCGAGAAGUU

>P178

CCGAGUGGUCUAAGGCGCCAG

>P179

UUCCUGCUCUUUUACCUCCAA

>P180

AUUAAACAUUUUUGAAGCUUU

>P181

AAGAUGCGGUUCGACUCCGAG

>P182

GACAUUGACAUUCAACCGUAC

>P183

UGGAAAUACGUAUCAAAACUG

>P184

CAGGGGUAGUUACAACACCGU

>P185

UAAUGACAGUUACUCUCACUC

>P186

GCUAAAUUUGUGUAAUUGGAG

>P187

GCUUGCCUCUUUUCUACAAGC

>P188

CUUUAUUUAUUCUCUGUAAGU

>P189

UACUAUUAUGUUGGGUUUGCA

>P190

UAAAGAAUGUUUACUGCCCUC

>P191

GGUAAAAUGUUAGGUGUUGAC

>P192

CUUCGUCCCCUCUUUGGGCCC

>P193

GUGGCCGCGCUGUGCCUGCUC

>P194

ACAGCAGCAUUUUCCAGAAUC

>P195

UGCCGGGAGCUUGGUGCGCUA

>P196

ACAAGGAGGUUGAAAUCCUUC

>P197

GGUCCUAGGUUGGACUCCUCC

>P198

CCUUUCAUCUUACAGGUGAAC

>P199

AAUUGUUUGGUAUGCGUCAGC

>P200

GCCCUUGGGAUGCUCUUUUCA

>P201

AUUUGCCUCCUAUCUGACCUU

>P202

ACAUGCUGCUUAAGGGAGCAC

>P203

GGCUCCCAAAUUGGAACCAGG

>P204

CGGCUGAACUUUCUGACCACA

>P205

ACUGCUGAAUUAAUGUGAUUU

>P206

ACCGGCGGGCUUCGAAACCGC

>P207

GAGCGAGAGAUUCAUGCCACC

>P208

UCUCUUUCUUUUCUUUGAAGU

>P209

UUUUGAUACUUGCCUAACAUG

>P210

GAACAUGCCCUUCCUGAAGAC

>P211

UUUUAUCAUUUUCAAGAUGGU

>P212

GUCCUAACGGUUAGGAAGGAU

>P213

GUUCACGCCGUACAGCUGGGU

>P214

GGCUGGAGGAUGCGGGCGUGC

>P215

AAAUUGGACUUAAGUCUGAAU

>P216

AGACUUUCCUUACCCAGAAGC

>P217

GCCAGAACUGUAGGUUAUAAG

>P218

GCGGCCGCGGUAAUGCUGGUG

>P219

AGUCAGCCAAUAAGUCUUUUC

>P220

UGUAGAAAUUUGUGCAAACAA

>P221

UGUUUUAAAAUGUUCUAAGUG

>P222

AGGACUGAUUUAACCCAAGAU

>P223

GCUCUUUCUGUGGCAAAACCA

>P224

CAGCCUAUUGUAUAUACACCA

>P225

GUCAUCCUCCUACGGACCAUG

>P226

CUAUUACACUUUGGGGAAUAU

>P227

AGUAGUAGGUUAAGGGUGGAA

>P228

GGGAAAGGACUAGACGCCAAG

>P229

UAGGGUAUCAUUUUUACGGGC

>P230

CCCUGGACACUUUUUAGGGCA

>P231

CCAGUCCCGCUGCAUUCUCGG

>P232

AGACUGAAGAUCUAAAGGUCC

>P233

AUCUGAUACGUCCUCUAUCCG

>P234

UUUGGUGUUUUCUCCCAUCCU

>P235

AAAAAGAGGAUCCGUGCUGUC

>P236

CAGCCGGCCGUACACUUGAAG

>P237

AAAUAAUGAAUUGAUGAGAAA

>P238

AAACAUUAGCUUUGCGCAGUG

>P239

CUUUAAGGAGUAAUUCUCCAG

>P240

GGUCCCGGGUUCAAAUCCCGG

>P241

UAGACCAAGUUAAGCAACAAC

>P242

CUCUGGAAUAUUCUCAAACUU

>P243

AUAAAAAUGGUAACAAACUCU

>P244

GUUCUCGGAUUUCUUGCUGAA

>P245

AACAGGUUAUUUAGUUUUUAA

>P246

UUUUUAAGUAUACAGGGGUCU

>P247

AACUGGAAUCUGAGGGUCUGG

>P248

GCUCUGAAUGUCAAAGUGAAG

>P249

GCGGGAACCAUUUUACGACAA

>P250

UCUGGUGAGUUAAUGGGGUUG

>P251

CCUGGACCUGUUCUGGUUAAA

>P252

GAGACCAGCCUACAGCCGCCU

>P253

CCCACCCGGAUACAAACCCGG

>P254

GAGACAAAAGUAUACACGUGA

>P255

AGGCUGAAGAUAAAGAAAAUC

>P256

AUUUGUCUGGUUAAUUCCGAU

>P257

UACUUCCUAGUUUUAUAGGUC

>P258

CACGCGCAUCUAUUGGAGCCU

>P259

UAUGGAGGCUUUUGAAUCGUA

>P260

CAGCCACUACUUCGAGGUGGA

>P261

AUUUAAACUCUGUUGCAAGUG

>P262

UAGAUGCACUUAAGUUAGCCU

>P263

GGGGGGAUGGUAUAUGGCCCU

>P264

GAAAUAUCAGUGUGUCAUCUC

>P265

UCUUUAUGUGUGAAGACACAA

>P266

CUAGUUAUUUUAAUAAAGGAA

>P267

CUCCUCAGGUUCUCUAUCGAC

>P268

UGCCAACACUUUGAGGGAUGC

>P269

GUUGUGGGCUUUCACGUACUG

>P270

GAUAAAAGGCUAAUAUCCAGA

>P271

UGCAGUCACUUAUUGUCCAGG

>P272

AAAUCAAGACUUGGAACGCCC

>P273

AGGUUUUAGUUUUUGCUUUAU

>P274

AAGGUAGCCGUUUGGCGUCGU

>P275

AGAGUUAACCUGCUGGCUGUG

>P276

CUGGAGCACUUACUCAGGUGG

>P277

AAUCGUCCUGUCAAAGGGAGU

>P278

CUUUUCCCAAUACCCCGCCAU

>P279

GGCUUUUGGAUUUGUGCAUAG

>P280

GGAAUUUGAAUAGGUCUACAG

>P281

CUAUUGAGUGUCUACUGUGUG

>P282

CGAUCGUGUAUUUAUGUAGGU

>P283

AGUGGUGUGAUUCGUGCAGAA

>P284

GGAAUAGUGUUCAAAUCACUG

>P285

AGGAAGGCCAUGGAAUCUGCU

>P286

GUGACGGAUUUAUACCUAUGC

>P287

UACUUGAAGGUACACCCCAUA

>P288

AAAAAGCGUAUUCUGAAUUUA

>P289

UUCUGGACUGUAGAGUUCUUU

>P290

GCACUGUACUUAAAAUCCCCC

>P291

UACUGUAAUUUUCCAUUACCU

>P292

UUCAUGGAGGUAUCGAUAUCC

>P293

AUGGACAGUUUAAAAUGGGCU

>P294

ACGAGAGGGUUACAGACCCUU

>P295

CUAAACCAUCUUCAAAACAUA

>P296

UUAAUGGAUAUAACCCAAUAG

>P297

UCUGAAGGGAUAAAGAUUACU

>P298

UGUGUGUGUGUGUGUGUGUCA

>P299

AGCAUGCCUGUAGUGUGACAU

>P300

GUCCAUAGUAUUUUUUUUUUU

>P301

GUGUGGCAAAUACCCAUAUCC

>P302

UAGACUGAGCUGUUUGAUUAU

>P303

UGUGGGCAGGUGUUUGAGAAG

>P304

AAAGCUUCGUUAUACAUUUGA

>P305

GCUACCAGGGUAUCGCCGUGG

>P306

CCCCAUGUUAUUCCUAAUAGG

>P307

CUGAGCACGCUACCAAAUCUC

>P308

GCCGCCCAGUUUCGGUUCCUA

>P309

UUUUUGUUAGUACACCAUCUC

>P310

CUAAUUAUGCUGGACUGUUUU

>P311

UGAAUGUUAAUUUCUGAGGUU

>P312

AUUCAGUUAAUGACACCAAAA

>P313

AGGCAGCAGCUACCAAGAAAC

>P314

CGGCACGCGCUUCGGGCAGCC

>P315

GUGCUGGCUGUAGGUGAAUGG

>P316

ACAGUUAAAAUUUCAAACAGC

>P317

GGGUGGUGACUUCAUAUGCCA

>P318

GAGAAGAAAUUAUUUGACAUU

>P319

CGUCGAGGUCUUCUUCUCGCG

>P320

UGUACCCAUAUAUAAUUCUCC

>P321

AGAAUAGCUUUAUCUUGGUUU

>P322

AAGAAGGUACUUCUUGGAGCC

>P323

AGGCAAUGGAUUUGAAUUUUA

>P324

AUAAAUGUUCUUGGUGUGGGA

>P325

GCUACUGAAAUAAAAGAAGUA

>P326

UUCAGUCAGCUCUGCAGGCUC

>P327

UCCUUCGUAUUAUACCCAGCC

>P328

CACUUUGUCGUAAGUAAUGGC

>P329

ACAGAUACACUACAGUAGAAG

>P330

UACGAGUGCCUAGAGUGCGGC

>P331

ACACUGUGGAUUGGACACCUG

>P332

CAAUAGCGUAUAUUAAAGUUG

>P333

AAGCUCCUAGUUGGAUCUUGG

>P334

CCAAAGCGUUUACUUUGAAAA

>P335

CAGCUAGGAAUAAUGGAAUAG

>P336

GAGGUGAAAUUCUUGGACCGG

>P337

UCGAAGACGAUCAGAUACCGU

>P338

GGGGGGAGUAUGGUUGCAAAG

>P339

UUGCAAUUAUUCCCCAUGAAG

>P340

AGGGGACGUGUAAACCAGUCC

>P341

AAAAAGUGCAUAUGUCGAAUG

>P342

GAAGCCUUGCUGAUGAGAGCC

>P343

CUGGCUACGGUAGCUACAGAU

>P344

GCACUGCUCAUACUAGACAGU

>P345

CCUGAGACGUUAUUUAAAACU

>P346

GGAUGUUGAAUAAAAGGCAGU

>P347

GUUCAAUGAAUAGAUGAUGCA

>P348

GAAGAGCAGUUAAAGAAAGAG

>P349

CCGACAGUGAUGGCUGGGACU

>P350

UGCUGUACCCUCAGAUCGGGG

>P351

GGACUAGAAAUCCAUUGGGGU

>P352

CCUCACCCGCUAGUCUGGCUG

>P353

AGAAAGAUUGUAGCGUUUUAC

>P354

AGUGGGAAGAUUCAUAGGUAG

>P355

AAUGUCUAUAUGAAGUAGGAA

>P356

CGAGGGCCGGUAUACAUUCGG

>P357

CCUGGGCUACUACGAUGGCGA

>P358

GUUCACCAGUUCCCCUCUUCU

>P359

AAGAGAGAUGUCCCCUGUGAU

>P360

UCAUACCUACUUUCAAAGUCA

>P361

GUGAGUUGGAUAAGGAGAUCA

>P362

AAAACAACUUUUGGGGGACAG

>P363

ACAGAAAUAAUUAAGAGGAGA

>P364

GAGCACAGAAUACCAGAAGCA

>P365

UGGGAUGUGUUACGGACGGCC

>P366

UCCGAGAACCUACACCGCGUG

>P367

UUGCUCCAGUUCAAGUCUGCU

>P368

AACCAAACAGUAAGCUUACUU

>P369

GUGCAAGGGUUCCACCCCUAC

>P370

UCCAAGCACUUUAAUUCCAAU

>P371

AGAAGCCUCCUAUAACAAAGG

>P372

CCUGGGGAUAUGAGUAACUUU

>P373

UCUCUCUCACUAUUCUUUGGU

>P374

GAUCUCAACCUAUUCUCAAAC

>P375

GGAAAAGCCUUCUCCCAAAGC

>P376

GAAGCAGCGGUGUCCCCAGCC

>P377

UUCACCAUCUUUCGUUUGAGU

>P378

CAUUGACCACUUCGGCUUCAA

>P379

CGAAGGUGGCUUUGCGCAUGC

>P380

UGUUUUAAUUUCUACACACCC

>P381

GAAGGACCCCUUCCCAAAUAG

>P382

GCAGCACUGGUAAAACCUUUC

>P383

UACUUGGGCAUAUGUUAAAUA

>P384

CUUAUGUUUGUUAGGACCAAA

>P385

UGGGAGCUGGUGUCUUAUGCC

>P386

UGUCUCAUGCUACAAGAAGAG

>P387

GACAAGGAGAUGGCUGAAGAG

>P388

CGGGAAAGUGUAGAGAAAACC

>P389

AUUUCAAGGAUAUGUGCCAGG

>P390

ACCAUCCUUUUCUUGGGGUUG

>P391

CAAAUUUAGGUAUGAAAUUCC

>P392

GGUUUGGACAUUGAAAUGGCU

>P393

GUACCGUUAUUGCCACCACAA

>P394

AGAUGUGACAUUCAACACUAA

>P395

GAGGCAGGCUUGGCGGUGGCG

>P396

GCAAUGGGUUUUCAUGUGAUC

>P397

GGAAGCCCCUUAAGUCGGGUA

>P398

CUCUUCAUGUUACACUAAUGA

>P399

CAAGUCGGCUUUGCUAUAAAC

>P400

CUCCCCAGACUGCUUCAGCCG

>P401

AGUACCCAACUCCCGCAUCCC

>P402

GAGUUCUCUUUUCUUUGUGAA

>P403

CCGCCCGCUUUAACCAGUGCA

>P404

GUCAAGAGCCUGCGGCUACAG

>P405

AAAAGGAGACUUCAUGUGCCC

>P406

GCCAAAUAUGUAGAGGACCUA

>P407

AAUGAGUGUGUUGCACAGACA

>P408

UCGUGUGCUGUGAUCGCACCU

>P409

AGUCCUCUUCUAAGCAAAGUG

>P410

AAGAUCUGUGUAUGGGAUUAA

>P411

UAUGGUUCCUUUGGUCGCUCG

>P412

UGCGUGCAUUUAUCAGAUCAA

>P413

UGACCACGGGUGACGGGGAAU

>P414

GGCCCCCCAGUCCCCCCAGGG

>P415

AUGGCGGUCCUGAGGUCUAGG

>P416

UAGCGAGGUGUAAAGAGGGCA

>P417

ACAACGACCCUAUCUACGUGA

>P418

AUCCCCCAUCUAAGAAUCCUA

>P419

UGGAGCGAGCUAUAGAGUCCU

>P420

GUAAGGAAAAUAACGAUUCGG

>P421

AUUUAUUAUUUGAGUGUUGUU

>P422

GUUUUGGACCUCCUGGUUCAG

>P423

AUUUCACAGGUAAGCCAAGAU

>P424

AUAUAUAGGAUUCGUGUUCGC

>P425

AGUUUGGGCAUUUGACUGACU

>P426

GAAGAAUCUGUGAAAGAAUAU

>P427

GCCCACCCUGUAGGAGCGUAA

>P428

AAGAACUUGGUGCAGGCACCA

>P429

UUUCUUCCUGUGUGUCCCCUA

>P430

UAAUUUUGCAUUAUAUCUAGG

>P431

AACUGAGCAGUUCAUGGAGAA

>P432

AAGAAGGAGUUUAUAACGUUC

>P433

AAGAGUGGGUUAAAGUCCUAG

>P434

UUAUUGGGCGUAUUUAUCAAU

>P435

GCGGCCCCUGUAGUGGCGCCG

>P436

UCCAAGCAUCUUCCGAAGAGU

>P437

CAAUUCUGUGUUCACCCUCAC

>P438

UCCUCAGCGUUACCGCCACAC

>P439

GGGUUCCAGGUUUCAGACACU

>P440

AAUGUCUCGGUAUAAAACACG

>P441

UGAAGUUCCCUAUCUACGGAA

>P442

CCUUAGUAACUAUGCAGUUUU

>P443

UCACCAACCAUUAAAGGUGUU

>P444

AAUUUUGACUUUAAAUCCUCU

>P445

UGUUCAUUUGUUCAUCCAUGU

>P446

ACCCACCCACUCCACACCACC

>P447

AGUCUUCCUUUAAUUUUGUUA

>P448

AGGGAACGAAUUCGUGGGUGC

>P449

CAACCACCUGUUUCACUAAUA

>P450

AUUACUCAUAUCAGAUUUAAA

>P451

UCCAACCAGAUUAGGGGAGAG

>P452

UACUGGAACAUACAACACACA

>P453

GAAAAGUCUGUAAUUCUUUCC

>P454

CAUCGCGCUGUUGGAAAGCGG

>P455

ACUCGUCCGCUAUCAAGAUUU

>P456

GAAUUUUUAAUCUGUUAUGCU

>P457

CUGAUGAACCUAUGCAAGUCC

>P458

CUAUGGGAUGUGUGGCAGAGC

>P459

CCUUGUCUAUUGUUGAAUCGG

>P460

GCAAUUUUGUUGUUACAGACU

>P461

ACUAUUAGAAUAUAGAAAUAC

>P462

AGGGAGCAAAUCCCACCUUUA

>P463

CGGGAUUACUUUGAGGACCAG

>P464

AAUCUAUGUGUGUGCGUGUGU

>P465

UUCUAACGUCUUCAUACCCAA

>P466

GUCACCGGAGUUUUUUACCUC

>P467

GUGGCCCGGAUGGUCAUGCUG

>P468

CCCCCCACCAUUCCUACCGCU

>P469

GAGACUGGUGUUCUCAAACCC

>P470

GUGGAUGGGGUGUCGUGGGCC

>P471

UUCAUCCGCAUGAGAAUGACU

>P472

CCAGGCUACUUUGGGAAAGUU

>P473

AUGCUGGGGUUCCAACCCUUC

>P474

UGUUAACCAGUUUUUUUUUUU

>P475

CGGGUAGCGGUGCAGUCCCGG

>P476

AGCUUUCACUUUAGAACAUUU

>P477

GCCAGGAAGCUUCGGGAAGCC

>P478

GAGCCACAGUUAAAUUCUGGC

>P479

CAGGUCCUUUUUGGAGGGCAA

>P480

AGAAAGUGCAUUUAUUGUCUU

>P481

GUACUCUGCUUGCGGAGGGCU

>P482

UAGUGUAAGUUAAAUCCUUAU

>P483

UGCGAGGGUCUAAGUAGGGUC

>P484

CUCACCUGCCUUCCCCUGAAC

>P485

ACCCCAAAGAUGGUGAACUGU

>P486

AAUAUGCAGUUUAAAUCUGCC

>P487

UUAAUUUAGGUUUCUUUUUUU

>P488

UUUUGAGUUUUUUUGUGGGGG

>P489

GGCUACAUUUUCUACCCCAGA

>P490

UAGCUUUGGGUGCUAAUGGUG

>P491

GGGAAAAAAUUCUCAGUUGUC

>P492

AGGCAGAGGUUGCGGUGAGCC

>P493

AGCCCCCCCCUUCACUUUCCU

>P494

GUUGUGUUGUUUACAAUACUC

>P495

GGCGAUGCCUUCCAUCAGUCG

**II. 495 false Ψ-site-containing sequences (negative samples)**

>N1

CCUUUUUCCUUCUCCUUCCUU

>N2

UUACCUGACCUUAACUUUUCC

>N3

UCUGAUAGACUGUCUUCAAUU

>N4

UGUAAAGCUGUACUUACGGGU

>N5

CGGGAACACUUCAUUACCACC

>N6

GUUCCCCAGCUGAAGAGUUCU

>N7

CAACAGUCUGUUUUACGGGAA

>N8

AUUAUUUGUUUACCACUUAGA

>N9

ACACAGGAUGUCAGCGUCAUC

>N10

UGACACUAGAUAGGGGGCCUG

>N11

UGUGGUGACAUCACAGUUCAU

>N12

GACUCCACAGUGAUGUACUGG

>N13

UCUUUAGGUCUUUGUAUAUAC

>N14

UGGAUAUUAUUGUUCUAUUGG

>N15

UUUCCCAGUCUGUCUGUUUCC

>N16

UGUUGAUACUUGGUCAUAGAG

>N17

UUCUCUUUUAUUAGAUUCAUU

>N18

AAUGAAUCAAUUUGUAUUCUU

>N19

AUGUCUUUUUUUCCCCCUGGA

>N20

UGGAUUUAGCUACUGUGUCAA

>N21

CCAUGCAGUUUUUAUCACUAU

>N22

UUUUUUGCUAUUCCAAAUGAA

>N23

GGGUGUUGUUUCCUAAGUUUC

>N24

CACCCUUGUCUAGUUUCUGAU

>N25

UUGCCUACUGUUUUACUGUAU

>N26

AAUGGUGAUAUUGUCAAAUGU

>N27

UUUCACCUAAUCUAAUGAAAU

>N28

UUGAAUUUCUUUUUAUAGUGU

>N29

UCUAAAGUACUCUUUCUUUGU

>N30

AUAGACCGAAUUGGGUAGUGU

>N31

UCCUUCUGUUUUUUAUUUUGU

>N32

ACCCGUCUGGUUCUCACAUAU

>N33

UUUUUUUAAGUUGAGAGACCA

>N34

UUAGGAGUUAUGGGACUGUUU

>N35

UCAUCUACAUUUUCCAAUUUU

>N36

GAGAAUCUGAUAAUUUUUUUU

>N37

UCUAUUCCUCUUGGGUGUAUU

>N38

UCCUUCCUUUUGUUCCUGCAC

>N39

UGGGAUUAUUUCAAUAGUCUU

>N40

UCUGUAGAUAUCUGUUAAAAC

>N41

UGAGGAUCAAUGUGUGUUUUG

>N42

UUAAUAACUUUGGAUAAAAGU

>N43

CAGUCCAAGAUCUUCUUAUGG

>N44

CUUUUUUUUUUUUUUAAAGAU

>N45

ACUGAAGCUGUCUUCAGACAC

>N46

UCGUUACGGAUGGUUGUGAGC

>N47

CACCAUGUGGUUGCUGGGAUU

>N48

AGCAGUCGGGUGCUCUUACCC

>N49

CGGGAGGAAAUUCUUUUCUGG

>N50

GUCACCUCUUUCUUUAGGUUA

>N51

UGUGUUAGGAUCUUUUUGCAU

>N52

AGAACUGGGUUAUGAUGGUGC

>N53

UCUCACCAUCUGGUUAUCUCA

>N54

UUGGUCCUAGUUGGGCCAGGU

>N55

AGUUUCUCCAUCUAACCUAAA

>N56

AAUGGUCAGCUGUGAAUGGAA

>N57

AGUCCAAGAAUGUAAUAGUUG

>N58

AUUCUCCUUAUGUAAACCUCC

>N59

GGAUCUAUGGUGCAUGCUGAU

>N60

UACAGGCAAUUGCUAUGUAGA

>N61

UAGCCGGGCAUGGUGGCACAC

>N62

AGUAGAUCUGUGAGGCAGUUA

>N63

UAAAAAGGGGUGUGGUCAGAA

>N64

GAUGGUGGCAUCUCAUGUAUU

>N65

AAUUCAGGGAUGGUGUCAGGU

>N66

CUUACAAUCCUCUCUAUUAUA

>N67

AUCCUAUGGUUGGUCCCUCAA

>N68

UUUAUUGUUCUCCAUCUUUGG

>N69

UGUUAGCCAAUCCCAAUUAAA

>N70

UCCCUACAGGUCCUGACAAAC

>N71

GGUUUUGGGGUGUGAGAGAGA

>N72

UUAAAAGGGAUCUCAAGGAAU

>N73

AAGAGUAAAAUAUCUACAAAA

>N74

GUAACAACUAUCAGGAGCUAC

>N75

CACUGUGCCUUCUAUCCCUUC

>N76

UUUGCCAGAAUUUCCAGAAGG

>N77

GUUUCCUCUUUGGCUCCUAUG

>N78

CAUAUCAACUUUUAUUCUCUC

>N79

UGCACCAAUUUGUCCAUAUGC

>N80

UAUUAUGUAUUUUCCUCAAUU

>N81

ACAUUUCCAAUGCUAGCCCAA

>N82

AAGUCCCCCAUGAACUCCUCC

>N83

GUUUGGAGGCUAAUUAUGGGA

>N84

CUGUAACUCCUUCCAUGGGUG

>N85

GGGCAAAGUGUAGACACUUUG

>N86

AUAUCUUGGGUAUUCUAAGUU

>N87

UUUGGGCUAGUAUCAACUUAU

>N88

AUGGCUGCUAUGAACAUACUG

>N89

CAGACUGAUUUUCAGAGUGGU

>N90

UGUACAAGCUUGCAAUUCCAC

>N91

GUUGUUUUGAUUUGCAUUUCC

>N92

UUAUUUGAUUUUCUGGAGUCC

>N93

CUUCGAGGCUUUUCCCCACUG

>N94

GCCAGCACCAUUUGUCGAAAA

>N95

UAACAACAAAUCCUGAAGAAA

>N96

AAUAACACAUUAAAGCAAUCA

>N97

AGCAAACCAAUAGCCAACAUC

>N98

UAGUCACAAAUAAUAUAAAAU

>N99

AGGAAGAAAUUAAAGAAGAUC

>N100

GUUUGGAUAAUUAAAAAAAGA

>N101

AAUGCAAAGCUCAGAGCAGGC

>N102

UCUAGGGAACUUUUGGGACUU

>N103

AAACUUAAGAUAUAUGAAAGG

>N104

AGGGAGGGUCUGAGGGACAUU

>N105

UGGGAUAGCAUUUGAAAUGCA

>N106

AUUAUGAAACUGUUGUGGUGU

>N107

UAAUGAAGAAUUGUAAGAUAA

>N108

AGUGUUUUGAUAUCUUGUAAA

>N109

AUUUUGAGCUUUAACUUCAGA

>N110

UGUGUGAUCUUCUGUUUGAAU

>N111

GAUCUUAAUCUAUUCUGUUCU

>N112

AAACAGCAUCUUGGCAAACAC

>N113

GAGGUGUCUAUUUAACUGACA

>N114

UGUAAUCCCCUUCAGGUCUUU

>N115

AGCAAUUGUGUUGUGGUUUGG

>N116

UAUAUAUAUUUUAGAUGGGUG

>N117

GGUGCCCUCAUCCCUCAACUG

>N118

CUCCCCUGUAUUUCAGCUAUU

>N119

AGAUCCCCCCUUUCCUCUGCU

>N120

UUCUGACCAGUAUAAGGUGGA

>N121

ACCAGAUGGAUUAUUGGUAAA

>N122

UGAACUCCUUUACAUUUAGCC

>N123

ACAGAUCUUUUACUUGCUUGG

>N124

AGUAUUUUUGUAUCAAUUUCA

>N125

UAACUGUGGCUUCAUAGAAUU

>N126

UUUUUGAGUAUAGGCUUUUGU

>N127

UUUGAUUAUUUCCUGCCAUGU

>N128

UUGACGAAAUUAUAAUUAAGU

>N129

GAAGGUAUAUUCUUUUGGUUU

>N130

CAUAACUUCUUACUUGGUCCU

>N131

CAUGGAGAGAUGUUCUUUACU

>N132

CUUUAUAUGUUCCUUGACAUU

>N133

UAUAAAAGGUUGAGGCUCUGG

>N134

GCAUCUAACCUCCCACCAGAA

>N135

AAGUGAAAAGUGUGAUGCAGU

>N136

CUCCCUCUUCUGCCCACUCUG

>N137

AGCACCAGAAUGCUGGAAGAA

>N138

GCCCAACAAAUCACCAUAGCA

>N139

UAAGUUCACAUAAACAGCUUC

>N140

UUGGUGGGCUUUGAAACUCUU

>N141

UAUUAGAAUGUAAAUUAGAGC

>N142

AAAACUACACUUCCUAAUAGG

>N143

CACUCACACCUCAAUUAACAA

>N144

CAUAUACAUAUAUAUACGCCC

>N145

ACAGUUUGCAUAGACAAACAC

>N146

UGGUGGAAAGUACAUGUUCUA

>N147

UGUUUCCAAUUACUUCACAAG

>N148

AAACUAGAUAUAGAUUAACCA

>N149

CAUGACAUCUUCAGGACACAG

>N150

GUUCUGUGCCUAUGUAAUCCA

>N151

AAAGAUCAAGUGAUCAUAGGU

>N152

GAAUAGACAUUGUUUAGAUCU

>N153

ACUAUUGUCCUGAGGCCUGAG

>N154

GGUAUUGUCAUGAUCAGCCAU

>N155

UGAAAUUUUUUUGUCUAAAAA

>N156

CUUUCCUAGAUUGGAAAGACU

>N157

UGUAAAAUGGUUAUUGGAGGG

>N158

CCCACUCCUCUCAGAGCCCUC

>N159

AAAGUUUUAUUGAACCCAGUG

>N160

UGGGGGACUAUUCUUGGUUGG

>N161

GCUAUCAGGGUCUGUGCUAUG

>N162

GCCAGUCAGGUGAGUGACAUC

>N163

GAUUGUGGUCUUGCCCCAAUC

>N164

ACCUGGGGAUUUACCCCUAUA

>N165

AUGGUUAAAGUAAAACAGCGG

>N166

CGAUGUCUUCUGUGUAACAAG

>N167

AACUGUGUGCUUUUCAAUACG

>N168

GCAGCUGGGGUAAGUGCUCUA

>N169

CCUUCUAAGAUAUCAAAUAAA

>N170

CCAAGGAAGGUUCACAUUUGA

>N171

UGUGGAUUUUUAUGGCAAAUG

>N172

UUUGCCUAUCUUGAGAGCACA

>N173

CAGAAAGCAAUGGAAGCCCUC

>N174

CUUCUAAUUUUUCAGAGGAAA

>N175

UUGAACAGCUUGUGGUGAGUA

>N176

ACUUGCCAGCUCAAAGAAUGG

>N177

GGCCAGGUUUUUGGCCACCAG

>N178

GCUCAAGGAAUACCAGAGGAG

>N179

UUAUAAUCAGUAGAAAUUGGU

>N180

GCAAGCUGGCUUUCGCCAGCC

>N181

AAGGCGGUCAUCACAGAGUGG

>N182

GAGGCCCCCAUGCUUUCUUCU

>N183

UGACCUCGACUGAACACCAGU

>N184

CAGUAUGUAAUCAAAAGAAAA

>N185

AGCACCUGAUUAGUUGCUCAC

>N186

UGCCAUCUUAUAAUGGCGAUU

>N187

GCAGGCUGCUUUCUGCCGAGA

>N188

GGGCAGACCUUCACUCACCAC

>N189

GGGCACCAGUUUGGCUGGUAC

>N190

ACUUGGGGACUCUCUGAAGAU

>N191

CAGGCAGACAUGGUUUGGAAA

>N192

UAAUGACCUGUUACAGAAAAC

>N193

CCUCUCAUUAUUGUUGAACCA

>N194

GGUAAGUUGCUUGGUUGAGGG

>N195

AUGCCCAACCUAUGGCAUCUA

>N196

GAGAGGAUUGUGAGGAACAGG

>N197

AUAAGGACAAUGGAAGAAGGA

>N198

AACUACUAGAUUCUUGACGUU

>N199

UUCAGCACAGUGGAACAAAGG

>N200

AAAAAAAGUUUUGUUUUAGCU

>N201

ACUGCCUACAUUAAGCUGGGC

>N202

GUCAGAGGCUUAGGCAGAAGG

>N203

GGUCAGAGAUUGAGGCUAACU

>N204

UGGACACGAAUACAAGAGGAC

>N205

ACCAGAAUUAUAAAAGUCUUC

>N206

CACACAUUUAUCCUCACACAA

>N207

AAAACAAGCCUCAACGGAUUC

>N208

AUCAAUGCUCUAGUUAAUGAU

>N209

UGGCAGAAAAUAACCUCAGAG

>N210

AUGGUCUCCCUACCAAAAAAA

>N211

AUGGGUUUAGUGCAGAGUUCU

>N212

AUCAGUAGCCUUUUUUUACUC

>N213

AUUUUUCAAAUUCAUUUGGAG

>N214

AAAUGAAAACUCAUAACAUUG

>N215

GUUAGGGAAAUGCACAUCAAA

>N216

AGGACCCAGCUAUACCACUCC

>N217

GUAUAGGAAGUGAGGCAGUAU

>N218

UAAUUUAAUUUAAAAAAAUAA

>N219

AGAAGGCAUAUUACCUAAUUC

>N220

GAUCAAAACAUCUGCCAGGAG

>N221

CCAGAAAAGAUAGCUCCAGAU

>N222

AAUAAAAUGAUCCAAGACCUA

>N223

AAAAGGGAAGUAGACACAUUA

>N224

AGGCUGGAGAUAGAAAACCUA

>N225

UACUUUUCCUUAACAUCUUUU

>N226

AACUAUCAAUUCUGAAUAUCU

>N227

CCACAUUCAUUAAAGAAACUU

>N228

GAUGAUGCCCUCCAGGUCCAU

>N229

AAACCAUCCCUGCAUCCCUGG

>N230

UAGUUUCUGUUUCCAUGAUCU

>N231

UCUGUUCUUGUGGCUGUCUUC

>N232

AUUUGUUGUUUUGAUUAUUAU

>N233

UUCAUUCUCAUCCACUCCUAU

>N234

GAUUGUGUUUUCCUGUUUUUC

>N235

CACUCUCUGCUGGCAAGCUCU

>N236

UGGUUUAAUAUAUGGAAAUCC

>N237

UAUAAGGAACUCAAGAAGGUG

>N238

GAUUGCAAGCUUGUACAACCA

>N239

AAUACAUUUUUAAAGGGGGGA

>N240

GGGGUUGAGGUCAUCCUUGGC

>N241

UUUUGUGCUGUCAUAGUGCUC

>N242

CAGAAUUGGCUAUUAUUCUUA

>N243

GUAAGGGCAGUACUCCAGAUG

>N244

AUUUAGUUCUUCCCUUAUUAA

>N245

UCAAACACACUAAAAUGCUGA

>N246

AAAGCUAGGUUCCAACCUGAA

>N247

GAGGGAAGCUUCUAAAACAAG

>N248

AACAACUGGGUAAGCAUAACC

>N249

AAUAUAAAUGUAAGAGAUGUG

>N250

GCCAAUGUCCUAUCCACACCC

>N251

UACAAAAAUGUUUCACAUUUA

>N252

GCUCAGGUCUUCAAACUUGUC

>N253

CCCCUGGAACUGGAGAUGGGC

>N254

UGAGCCCAGGUUCUCUGGAAG

>N255

AUCUUUCAAUUUUUUAUGAGU

>N256

UACUUACAUUUAAUAAAUAAA

>N257

AGCCGUGCCGUGAGCAAUCGC

>N258

GGAGAGAGAGUAGGAGCAAAA

>N259

GGAGUGAGUUUGGAAGGAAGG

>N260

CGCCACUAGAUGAGUUUAAGG

>N261

UUCAGGGUCCUAGUGCACCUC

>N262

UCAGCGCCAAUUUCACUAUAG

>N263

CGUGGACUUUUGAAUUACUUA

>N264

CAGGGUUGUGUACUAAGUGUG

>N265

CUCCACCCGAUUCAUGCUGAU

>N266

UUCCCUCACCUAACAAGGCCU

>N267

UGAAAACUGAUCAAGGGUAGC

>N268

GGGUCCACCCUAAUGCUUCCC

>N269

AAGAACCUCAUAUACUAAUUG

>N270

UUACCUCCUAUUCAGGAAAAA

>N271

UUGAUAAUCAUUUUCCUAAAC

>N272

CACUUGCAGAUGGGGUGAUAG

>N273

AGCAAUAUAAUGAGACAUCAC

>N274

UCUUAUUUUAUAAAACAAAAA

>N275

AAAAUCACUCUUUAAUAGAAC

>N276

CCCAAGAUACUUCCAACGCGG

>N277

AAAAGGGACUUUUCAUACAGG

>N278

AACUGCACAGUAUUUGGGAAU

>N279

GAUGAGACUCUCGAGGCCCAG

>N280

GUGGAUCCAUUGCACUGGGAU

>N281

UGGCACUGGCUUCCACUGGGC

>N282

UCCGAGUGAAUUCUUGCCAUU

>N283

UCAAGUAACAUAUAAAGGCAA

>N284

AAUGAAAGCAUUUCUAAGAGG

>N285

UUAAACGCUAUCCCCAUCAAA

>N286

UUCUCAAGGAUAAAAGAACCU

>N287

CCCACACCUAUGGUCACUUCA

>N288

UUCCGGAAGAUUUUACCUCAG

>N289

GCUGCUUGCAUGACCACCUUG

>N290

CAGGCAGACCUUUCAGACAAG

>N291

GUAUAAGAGAUAAAAGCAUAU

>N292

UAAUAUAUAAUAUAUAUUGAG

>N293

UCUGCAACCUUAUAGAUGAAA

>N294

CAUUAAUAUCUGGCCUGCUUA

>N295

CAACAAUUUUUUGUCAAAAAA

>N296

UGGGGUUCUGUGUCUGUGGGU

>N297

UGGAUGGCCUUUCCUUCAGCC

>N298

ACCCCACAUCUACUUAUUUCU

>N299

CCCCCCUUUUUUAAUUUGGGU

>N300

CAUAAGUGAAUUUGGCCUGUA

>N301

UUUUUUACAUUUGGUGACAGG

>N302

CUCUCUCCCAUUAGGAUUUGA

>N303

AGAGACAACAUGGCCAAGGAA

>N304

UUUUGUCAAAUUGACACAACC

>N305

UUCAAAGAUUUGUAUUUAGAA

>N306

GUGCUGAUGUUGAGACAUGAU

>N307

CACUAUAUUGUUGAGAAGCAA

>N308

CACUCUGGUUUGAGAAUCUAG

>N309

AGGAAUAGGGUUAAGAAAAUU

>N310

CAACAUUACCUUAAAAUUGGU

>N311

UAAAACACUAUUUUCUUAAUG

>N312

CUAUGGUUGCUGCCUUGCAGG

>N313

UAAAAAGUCCUAAAAAACUUG

>N314

AGGAGAAGCUUUAGCUUCAGC

>N315

UAAAUAUCGAUUUAUUUUUUG

>N316

CUAGCAUCCCUAGAUUUCCUA

>N317

AGAGGGAAGCUUCCUGCUGAG

>N318

AAGAACAUUGUCUCCUCUUCC

>N319

GUGCAACAGUUAUAUUUACUA

>N320

CCCCUGGCUUUGUUGGAACCC

>N321

GGGACUUGUUUGCCCCUAUGG

>N322

CUCAGGGUCGUGAGUUCGAGC

>N323

GGCUGGCAUUUGUGUUCUCUG

>N324

GGGAUUUGGAUAUGGGGAUAU

>N325

AGACUCUAACUUACCUGACUG

>N326

UAGUGUAAGGUGGGGUUCAGG

>N327

GACAGUGGGCUACACACAGAA

>N328

CAAUCAUAUCUCCUGGACCCC

>N329

GCAUUCUGGAUGAGAGUAUAG

>N330

AUUUCUCUCCUUGUGAAUUAC

>N331

GCUAAGACUUUGAAUCUAUAU

>N332

UAAAGACGAAUAAUUGUUGGU

>N333

ACCUGAGAAUUAAGUUAUCUC

>N334

AACCAAAACUUAGGCUGUUUC

>N335

AGUUGCUUUGUCAUGGUGUCU

>N336

ACCCUUGUGUUCCAGGAGCGG

>N337

AUUAAAGGUGUGCAAAACCGU

>N338

AUUAGGAAAGUUUUAAGUAUA

>N339

AGAGACAAAGUUUGGAGUUGA

>N340

CUCCAUAUGAUGUGUAUAAAC

>N341

CUCUCAAUCGUGUGGGGACCA

>N342

AAGAUGGGAUUCUGUGGCUUC

>N343

CUCUCCUGGUUUGUGUACCAA

>N344

UAGAAAUGGAUGGAUCCAGAA

>N345

CUGGGCUACAUAAGUGAAGGG

>N346

UGUGUGUGUGUGUGUGUAUGU

>N347

CCAAGGCUGUUGGGAGCCGCC

>N348

UAAUUCUCCUUAAAAGGGACG

>N349

UUCCGGUUUGUUGCGUUCUUC

>N350

AAGAAGUAAGUUGGGGAGGAA

>N351

AGCCACAAAAUCCCAGCUUGC

>N352

GAGGUUAGACUCAGGGGAAAG

>N353

CAUGAUCAUCUCGUUAGAUGU

>N354

AGAGAACACCUAAACCUGAUA

>N355

GGGCCCCCAAUGGAGGAGCUA

>N356

CGUAGAGGAGUGGGGGGAGGG

>N357

GCAGUUUGAUUGCAAGGUCAG

>N358

ACAGGAGAAUUGGAACCCUUA

>N359

UUUUAUUGUCUUAUGGGGAGU

>N360

AAGUACAAACUCAAGAUCCUC

>N361

GCUCAGUUGGUAGAGCAUGAG

>N362

GACAUGUUAUUUUAGUUGGGU

>N363

GGAUGAGUCCUCCUCUUUUGC

>N364

ACAGUCUUGGUUUCAUCUUUC

>N365

GACAGUGAUUUUAUACUGAAA

>N366

GAAUGUCAGCUGAGAAAAUUU

>N367

GGCUGGGUGAUGUAGUUUUCA

>N368

CAGCCUGGGCUAACCAGCAUG

>N369

GUACCACUACUAUUUAUGGGG

>N370

UCUAUUGGUAUGGGUUGGGAA

>N371

UGGGUCUUCAUGUAGAGCUUG

>N372

UGGUACCUGGUAUCUGCCUAA

>N373

UCCAUGGUGAUCUGAUAGAAU

>N374

AAAGGUUUUAUUCGGCUUAUA

>N375

UGGGUCAAGUUAACACCUAAA

>N376

CACUGUUCUGUCAAUGUUUAA

>N377

UAAUAAAAGCUAGAGCUACUG

>N378

GUAGCCUUGUUUGAGGAAGUG

>N379

UACUCUUACAUCAGGUUCAAA

>N380

GUUCUUAUCAUAUAUUUCUUU

>N381

UUUUGCUUGGUAUAGUUGACU

>N382

CCGGAUGGCCUGUGUGAAAAU

>N383

UGAUGAUUAAUGUGGGAGGGG

>N384

CUAACAAAACUUUUUUUUUUU

>N385

AUCCUAAUCCUCCACAGUUGU

>N386

GGGGGGGGGGUAGGUAGGAAA

>N387

ACCCAGCGUGUGCCUCUGGGU

>N388

CUAAGACCUCUGGUGAAUGGA

>N389

UUCCCAAAUCUAAAGAAUGAG

>N390

UUACAGGAAAUCCAUCUCAGG

>N391

CAAAAGGAUAUACUUUCUUCU

>N392

AAGAAAUUAGUGAAACAACAC

>N393

GGAAAAGCCUUGAAGAUAUGG

>N394

AACAUAGUACUAGCGGAGGAU

>N395

UUAGGAAAGUUAGAAAAGCAC

>N396

AACUAAGGGUUUGAGUCAUUC

>N397

GAACAUUUUUUUCAGGUGAUU

>N398

UCUUCCCCAAUACUCCCAGAG

>N399

UUGUCCCAUAUAUCCUAUCAG

>N400

CAUUCUGAGGUUGUUCCAGGA

>N401

CCGGGACCUUUGGGGGAGCCG

>N402

UGUGCCUUUUUAGCCGAAACU

>N403

GUGGGGGAAUUUUUGAACCCC

>N404

UCCUCUGUGUUGAGGACAAGG

>N405

UCCCCGGGGCUUCGGCCUCGG

>N406

CGGCUCCGAGUCCCCCCCGCC

>N407

GAGGUGGGACUCAAACCCACG

>N408

CCUGGUAUGGUGUAGAGGAGA

>N409

AGAGGAACACUCCUCCAUUGU

>N410

UCCCCCUCCUUAGAUUUGGGA

>N411

ACUCUUUUUUUUUUUUUACAA

>N412

AUUACAAGACUGAUGGGACUC

>N413

UAGGUCCCAGUAUAGCCUCAC

>N414

GGCAAGUUGAUUCCCCCAGAG

>N415

GAUCAAAAACUAAAGAGAUUG

>N416

UGUGGGGGCAUCUUGAGGACU

>N417

CCACAAAUCUUUCAACCCAAA

>N418

GGGGAAUAUAUUCUAGCUGGG

>N419

UACACCCAACUUCUCAAAAGU

>N420

UAAAAGAACUUCUGGAGAUAU

>N421

GAAUGGCAAAUCAAAAAGACA

>N422

UAAGUGGAUAUUUCUCAUAAA

>N423

UUACAGGUAAUAUAAGACCUG

>N424

ACUGUGGCUAUGGAACGCUCA

>N425

UGUGUCUGGCUGUUUGUCCCA

>N426

CAUUCCAUCUUUGUAUCAGAC

>N427

GUCAUGCCAUUAUAAAUUUCA

>N428

UUCAUAAUGUUGUUCCACCUA

>N429

AGAUGAAAGGUGUAGCCCAGA

>N430

AAAGAAGAAAUAAUACCCCAG

>N431

UUACCAUAGUUAAAUAUAGAA

>N432

ACAACAGGAAUUGGAGGGUAC

>N433

GAUAAAGACUUCCACAGCCAG

>N434

UAUUUGCAUGUAAAGCUGUUG

>N435

GACCCCAGGUUUGAAGCACAC

>N436

CUGCUUUUCUUUGGUCUCAAG

>N437

AUUGAGUGUGUGGGCAACAAA

>N438

GAUAAAUGCGUACUAGAAAAG

>N439

CAAGAUAAACUGCAGAAUGUU

>N440

UUUUUUUAGGUGAAAAGAACA

>N441

GAUAGUGGCCUCCAGAGGCUG

>N442

UUUUUGCUUUUUUUGUUUUUU

>N443

UAUAAAAUUCUGUGUAUAGCU

>N444

AUGCCUUUAAUCCCAGCCCUC

>N445

GGGCACUCAUUCACCCACCAG

>N446

UAAGGAUUUAUUUAUAGUCAG

>N447

UGCCCCUGAAUUAGACAUCAC

>N448

CUGAGGAAACUGCCAACUGUU

>N449

CACCUCUUGUUAACAUUCCUU

>N450

UUUCUCUAGCUCCUCCAUUGG

>N451

AGGAGAGGUAUUGCUGGAUCC

>N452

CUCUAAUGAAUGUGGCUGCCC

>N453

GGAGCGGCUCUCUUAGAACCG

>N454

CAUCCAGGGCUGGAGAGAUGG

>N455

CUGGUGUGUCUGAAAACAGCU

>N456

GGGAAAUUGCUAAAACAAUCA

>N457

CGAACCUUCCUGAUGCACCAG

>N458

ACAUUCCAUUUUAUAAUUGUC

>N459

GCCACACACGUGCACCUUUCU

>N460

UACGGGCAAUUUUUUCACAAC

>N461

ACUAAAGUUGUGAAGAACGUU

>N462

UCCCAGCCAAUCGUUGGCCCA

>N463

UUUCCGGCUCUGCCUCGCCUC

>N464

CAUGUGUGGGUUUUAUCAAUA

>N465

AGAUGUAGUCUCCCCUCCCCU

>N466

CUUUGUGCAGUCAGGCUUGUC

>N467

GCAAAAACAAUCCAACUAAUG

>N468

GGGAAUUACUUGUAGGGAUAA

>N469

CUCUGCUCCCUGCGCUAAUUU

>N470

GCUGUCCUUUUAAUAACUAGG

>N471

UUGUAUAACAUAGACAGCUUU

>N472

UGAGAGGAGUUCAUUAAGGGG

>N473

CUGAAAGCGGUCCAUGGGUCC

>N474

AAUGGGAUUAUAAUACGGCAC

>N475

CCCCCCUUGGUGACUAAACUU

>N476

ACCCAGGUUUUGGGACCAUGG

>N477

CACAGAGCAAUAUAUCGACAC

>N478

AAUACUAAAAUUGGUUAGUAA

>N479

CCGUAGGGGAUAAGAAUCGCC

>N480

GUGGUUACUCUAACCAAAGGG

>N481

GGGAACGAGAUGCUCAACGGG

>N482

GCCCUUAUGAUACUAAUGAAA

>N483

UGAGCAGACCUGAUAAAAUUU

>N484

UCCCAAUGACUUAACCACCAA

>N485

UAGCUAGAAAUAUAUCAGAGG

>N486

GCCUUUUUUUUUUUUUUUUUU

>N487

UCGAUUUUAUUCAAUAUUAUU

>N488

GGGUCUAGCUUUUCGGGUGUG

>N489

AAACGUGAUUUAAAUAAUGUG

>N490

GACACAGCACUUGAAUACCCA

>N491

UGGGGAAGGGUUGUUUCUCAG

>N492

UCAAGGGGGGUUAGUAGAAAU

>N493

CUAUGACAAAUUAGUUGGACA

>N494

UGUCAGGGACUUAAGUUUUAC

>N495

ACAGGAAGAAUUGAGUCACAC
