# Supplementary material for: PseUI: Pseudouridine sites identification based on RNA sequence information
Source: BMC Bioinformatics. 2018 Aug 29;19:306. doi: 10.1186/s12859-018-2321-0 (PMC6114832; doi:10.1186/s12859-018-2321-0)
Supplement: Supplementary file 2 — The benchmark dataset S_628 for S.cerevisiae. The benchmark dataset H_990, S_628, and M_944 is formed by 495, 314 and 472 Ψ-site-containing sequences and 495, 314 and 472 false Ψ-site-containing sequences, respectively. Both H_200 and S_200 are formed by 100 Ψ-site-containing sequences and 100 false Ψ-site-containing sequences, and none of the samples included here occur in the corresponding benchmark datasets. Each of these samples for H.sapiens and M.musculus is 21-bp long with the uridine located at the center, and each of these samples for S.cerevisiae is 31-bp long with the uridine located at the center. None of the sequences included here has ≥60% pairwise sequence identity to any other in a same subset. (DOCX 45 kb) [file 12859_2018_2321_MOESM2_ESM.docx]

**The benchmark dataset S_628 for *S.cerevisiae*.** It is formed by 314 Ψ-site-containing sequences and 314 false ψ-site-containing sequences. Each of these samples is 31-bp long with the uridine located at the center. None of the sequences included here has $\geq60\%$ pairwise sequence identity to any other in a same subset.

**I. 314 Ψ-site-containing sequences (positive samples)**

>P1

GGUGCUAACAUGGGUUCUACACCCGAGGCUA

>P2

CGAGGCUAUUUCUGUUUUCGAAACAGCGCGU

>P3

GGGCUAGUAAAAUUGUAGUGUCUGAAAUUGC

>P4

UCGUCUCUUUGAUUUUCUUUAAGGCGCUAUU

>P5

GGCGUUGUGGAAGUUUCAGGCCUUCGAACUU

>P6

AAAGACACUGGUUCAUGAUGACUGAACAAAG

>P7

ACGAGUGGGGACAGUUCAAUUCUGUGGUAAC

>P8

GACAAGACUUCGAAUUUGAACUUCAACCACA

>P9

AGUACUUGGAUAAUGUUGUCAUUGAGACCGC

>P10

GAAAAAUGAGCAGUUUAAGGCAAGACAUCAC

>P11

GGAAACCGGGCGUAUUGAGCCCUCGUGCUCG

>P12

GACGUCCUUACCUUGUUCACCCAAGACUUCU

>P13

GCUCUAUGAUGUCCUUUUCUACCAUAUCGUC

>P14

GGAGCUGCCCAACUAUAACUCUUCGCUUACU

>P15

GGUUUUAAAGUAUUCUUUACAUUGAGUUUCG

>P16

GGAGUACACUCAUUGUAAAGGAAAACCAUGA

>P17

UACGGGCGUGUGGUCUAGUGGUAUGAUUCUC

>P18

UGGUAUGAUUCUCGCUUUGGGCGACUUCCUG

>P19

CAGGAAGACAAAGCAUGCGAGAGGCCCUGGG

>P20

CGAGAGGCCCUGGGUUCAAUUCCCAGCUCGC

>P21

UCGAUCGUCGUGACAUCACAAUUGUUGACGA

>P22

AAUAAUUUAUACGUAUACUACAUCGAGACCA

>P23

UACUCAACUGCAAUGUAGUGGUUUACCGAAC

>P24

UAUUAAAAACCAGUUUCAGGCCAGUGUCUUG

>P25

UAUAAAUUUUUACGCUACACACGUCAUCGAC

>P26

AAAAAUGCAAUCUGAUACCCAAGAGGCAAAC

>P27

UUCUUUCCAAAAGUAUUGAAAAAAUGCAAUC

>P28

GUUAGACGUUUCAGCUUCCAAAACAGAAGAA

>P29

GUCUAAGGCGCCUGAUUCAAGAAAUAUCUUG

>P30

GAACUGUGGGAAUACUCAGGUAUCGUAAGAU

>P31

UUUGCCCGCGCAGGUUCGAGUCCUGCAGUUG

>P32

UCAUAAUUAAGCUUUUAGUAGAAGUCAUUAC

>P33

GGCAGACUUAAGAUCUGUUGGACGGUUGUCC

>P34

UGUCCGCGCGAGUUCUAACCUCGCAUCCUUC

>P35

ACUUCGGUCAAGUCAUCUGGAGGUCCUGUGU

>P36

ACGGAUCCAUCAAAUUUGAGUGUUAUAUAUA

>P37

UUUACUAUUAUUGGAUGUUGCGCCAUUGUCC

>P38

AACUGUUCCUGCAUAUUUCAAUGAUUCUCAA

>P39

UCCGUGUCAUAUUUGUUCACCCAUGCUCCAG

>P40

CUAACGGUAUAGUUGUAGCCACUGAGCAUAA

>P41

AUUAUAACAAGAAAAUUGACAUGUACUUUAG

>P42

UUACCCAAUUAAGGGUAUUCCAGGCGAUGCC

>P43

CUACCGCUUACAUUGUAGUGCUAUUACAAGG

>P44

AAUCUAUUCUAAGAAUUGCAGAAUCUUUUGC

>P45

GCGGGGAUCAGCGGUUCGAUCCCGCUAGAGA

>P46

AAUCCAACGUUGCCAUCGUUGGGCCCCCGGU

>P47

CUAUAAUUGCUGAUCUUCCACUUCCCGAUCU

>P48

AAACGGAAGAAAACUUGGUGCCCCAGGCCAA

>P49

GCGCCACACUAGUAAUGUGGAGAUCAUCGGU

>P50

ACUACAAGACCAGAUUGAAAAAGUCACUGGU

>P51

CUAUCCGUCCAGACAUUGUCCACACUGUUUU

>P52

ACCUCCGCUGAAUCCUGGGGUACCGGUCGUG

>P53

AUUGAACCGCUUAAGUUCCGUUGUGAAGUUU

>P54

CCUGCUGCUCUUUUCUUUUCCAAAGAAUUUC

>P55

AUGCUGGUGUCAGUGUAGAUGCUUGUGUGUG

>P56

ACAGCUAAUUGUUUAUUAGCAAAGUUUGCAC

>P57

UUUUCAAGAUGGUUUUUUUAGGUAUCUAUUA

>P58

ACGAAAAUUCUGCUGUAGUCUUGGUUAAGCA

>P59

UGACCUUUAUGUAUUUUGCUUCGUGGCUGCU

>P60

GCCACCAAGCGCAAGUGGGUAUUCUUUAUAA

>P61

GCGAAGAUCCCGAGUUCGAACCUCGGUUGGA

>P62

GUUCAAGACGUCGCCUUUACACGGCGAAGAU

>P63

AUUGAGAAAAGUUUAUGAGUUAGCCAAACCU

>P64

AUGUAUUUUCUCAUGUAGCUAGUUGGGGUUG

>P65

CGUAAUGGCAACGCGUCUGACUUCUAAUCAG

>P66

CAGAAGAUUAUGGGUUCGACCCCCAUCGUGA

>P67

GGUCAGAAUGGGCGCUUGUCGCGUGCCAGAU

>P68

CAAGCUAUGCGUUUAUUAGAAUCCAUCAGGA

>P69

AUAUUUGGGUAUAUCUGACGAUGAUAUUACU

>P70

UUACAACCCAAAGACUGUUCCAUUCGUCCCA

>P71

UGCUCACAUUGCUUGUAGAUUCGACGAAUUG

>P72

UAAGGAUUAUAUUUGUAAUUGGCUGGGAAAC

>P73

CGGUUGCUGUCUCUUUUGAAUUGAUUGGGAU

>P74

GAAUUGUGUGCGCAGUUACCAAUCACCGUUU

>P75

AAGAACUGAAUCUUGUAGAAUUGAAGGACAG

>P76

CAAGCACUCCAAGUGUAGACAAGAAUUUUUG

>P77

UGGUAGCGCAGCAGAUUGCAAAUCUGUUGGU

>P78

GUAACGGCUAUCACAUCACGCUUUCACCGUG

>P79

UGUUAAGUACAACUGUAAUUGGUUCAGUUGC

>P80

GACUAGAUCGUCAUAUGAGAAUGAUAAUAAG

>P81

AUACUGCUGAGAGAGUUCUGGAAGAGAAAAA

>P82

GAAGAAAUGGUGCUAUAGUAGGUUGGGAGAU

>P83

GGCUUAGAUCAAGUGUAGUAUCUGUUCUUUU

>P84

CGGUAUCCAAACCGGUGCUGGUGUUGUCGAC

>P85

CCCAGAUCGUUGUUGUAGACUCUCUGGAAGA

>P86

AAAAUUUGAAAACUUUGGAACCAACAACGAA

>P87

GAGAAUAAUGAGCUUUAUGUGGAUUUGUUAG

>P88

GAGCAUUGACAGGCAUAGGAGGUGGUGGCUU

>P89

AGGCCAAAAUGUUUAUAGAUAAUAAAAGUUG

>P90

UCUCUUUACAGACGAUACUACAUUCCGCCAU

>P91

UAAACAAAUUCCCUGUAAUCCCUGAACACAC

>P92

CGCCACUACUCCAAAUGCUACGCAGCCAAAU

>P93

UAAAAAUCAGUGAUGUAAUGGAGAGCUUCAU

>P94

GAAGAGAGCUUGUCUUCUUAUCAGCCGUCCA

>P95

AGGCCUUUGGUUCUGUAAUCUUCUUCACCGA

>P96

UAACAAGAAACGUGAUGGUACACACAAAGCU

>P97

GGUGGCUCCACGCCGUUCAGAUUUGAGACCU

>P98

GUUUACACAUUUUGAUACAACCGUAGACGGC

>P99

UGGAGCGUGCAGCUAUGCAUGGGAGUGAUUU

>P100

AUUUACAUAUGGCUGUUCCAUCUCUAAUUUA

>P101

GGUCAACUAAACAUAUUUAUCAGGGCAUUUC

>P102

AAGAUCCAAUAACUAUUACUCGCCACUUGCA

>P103

UCAGCUCGUUCGCUCUGGAAAGGAUCAUUUU

>P104

GAGAUGAAGAAGAUGUAGAGGGAACAGAUGG

>P105

GCGUAUGACUCUUAAUCAUAAGGUUAGGGGU

>P106

GAAAGCAUUCAAACUUUAUGAUUACAGUGUU

>P107

UAGGGGAUUUGUGACUUUCAAGGCAACGUCC

>P108

AAGGACCGGUAUUAUUGUAGGGGAUUUGUGA

>P109

AACGCUGUCCCUGAAUGGCAGAAUCAUUACC

>P110

GAAAUUAAACUAAUUUUACAACAAUUAGUAA

>P111

GAGCAAUAUUUCAAAUCUCUGAUUGAUUCUG

>P112

UGACGUGUAAUAGUUUUAUUUCUUUGAAAAA

>P113

AAGCAAUUGGAAAAAUCUAUUAACCCAGAUG

>P114

AACUCAGCAAACUAUUAUCAACAUCAUGUCG

>P115

UAAGUGCUUUGUUUAUACGAUAUAGCACUUU

>P116

GUUUUGUUCUUAUAUUUCCAGCUAGGGAGCC

>P117

CAUUCAUGGUUCUUGUGGGCUGGAUGAUCGA

>P118

UUACACUCACACCAAUGAUGGCGAUAGUCUC

>P119

CGAGAAGAUAGAGUGUAGCAAUGAUUAAGCG

>P120

AGGUGGCCAUUUGGCUUUUAGAGCGCUCCUG

>P121

ACUUCAAAACUACUUUUUAAGACCAUCCUUU

>P122

UCUCCGAAGAACUCAUCCUUGUUAGCUCAGU

>P123

UCAGUUGGUAGAGCGUUCGGCUUUUAAGCGC

>P124

GGUUCGAGCCCCCUAUGAGGAGUUCUUUCUU

>P125

UUUGCCCCGUAUCUGUAGCCAAUUGAAAGGU

>P126

CGCGCCCAUAUCCUGUAAAUGUAGAGGCAGU

>P127

UGCAAAAAUGGUGCAUCAUCCACAUCUGUUA

>P128

UUGGUAUUCACUCUGUAGUAAUCAAGCCAGA

>P129

CCACUGACUACACUGUAGUCACUGAAUAUAC

>P130

CAGCCAACCCAAGUCUAACCGUCUCCACAGU

>P131

CUUCAAGAAAUCUUGUUCCUCGUGGCCCAAU

>P132

GCUUCCACCUUACUUUGAACUAGCCAAAAGG

>P133

UUCAUUUUUCCCAGUUUGUGGAAGAACCUGC

>P134

GUGAAAGAAAAACUUUUGGACGGAAACAAGA

>P135

AUCCGCCUAUAAGUUUGAGGCCUUAUUUGGA

>P136

AGGAUGACGCGAGUGUAGAUGACGUAGCCAA

>P137

CACGACAGACCAGGUUUGUUGUCCAUGGCCA

>P138

CCAACCAAUUGGCCGUGUCGUUUUCAAGUUG

>P139

ACACACGUCAAACUAUUGACUCCAGAAGAAA

>P140

ACAAACUCGUUACUAUUACUCUACUGUCUUU

>P141

GUCAAAAAGGCAAUGUAGAGGAGUCUGAUUU

>P142

AAUUUGACAUAUCUAUAUUUAAGGAGACUAG

>P143

AUUAACUUGACAGGUUCGUUUUAUUUGCCCA

>P144

CUGCUUUAUUUUUCUUUGAUAUGGGCAAUGG

>P145

CUGGUGCUAUUAACUUCUACUACGGUUACGU

>P146

AAGAACACAAACUGUUUAAGGCACAGUACGC

>P147

UUGCUCCAUUUUGGUUCGAUCCCUUAAUGCC

>P148

CAGGUGAUCCUGAUGUAGACUUAAACCCUGU

>P149

UUCCGAUACGGAGACUGUUUUGGUCUCUACG

>P150

GAACAAAAAGAGCCAUGAUCAGCUCUUACAA

>P151

UUGGAGAAUUCUUGUUGGAUUGUCACUAAUU

>P152

UGGUAGGAAACGUUGUUUAAUGGGUUCGAAU

>P153

UGAAUAACACCUCUGUAGGCGAAGAAAAGAC

>P154

UGUAAUAGAUUUUUAUCAGAUAAAGGAUAGG

>P155

UGAACGUGGUUGGGAUAUGGAGAUUGGGAGA

>P156

UUCUCUCCUCUACAUUUAAUAUGUUCUAUAU

>P157

AUUGAGGUUGCGACUUUUUCGUUUUUGCUUU

>P158

GAAGGGCUCUGCCACUCAGGGCGUGGUAAGA

>P159

CAUUUAGUAGGUCGUUUAGCUUCCGUUGUUG

>P160

UUAUGAAACCAUGGGUUUCUUGACCCUACCA

>P161

CAGAUGUGCCAACUGUGUCAAGGAAAGAAUC

>P162

UAUUCCGUGAUAGUUUAAUGGUCAGAAUGGG

>P163

CUACUUUAAAUGUUGUCCACCCAUCCGCUCU

>P164

AUAAGCUAGCACCUGUUGACAAGAAAGGCCA

>P165

AGGUCAGGCGCUUUCUUUGGGAGAAAGACCU

>P166

AAAGACCUAGUUAGUUCGAGUCUAUCCUAUC

>P167

UGGUAAAACAGUUGAUUUCAAAUCAAUCAUU

>P168

AUAAAAAUAAAAUUAUUAAUAAUAAUAAUAC

>P169

GAGUGGUUUAAAGUGUGAUAUUUGAGCUAUC

>P170

UUCAAUGGUAGAAAAUACGCUUGUGGUGCGU

>P171

UUACAGUAUGAAGGUUCAAGUCCUUUAAAUA

>P172

GGUAAGUUACCAAAAUUUGAGUUUGGAGUUU

>P173

ACAUUGAAUGCGAGUUCGAUUCUCGCUAUCU

>P174

UCUAAUUAUAUAGGUUCAAAUCCUAUAAGAU

>P175

GGUACAAAUAUAGGUUCAAUCCCUGUUAGUU

>P176

UGUUGGUAGUUGGAGUUGAGCUGUAAACUCA

>P177

CAAAUCAAGAAGGUAUUUUAGAAUUACAUGA

>P178

UGGUAAAGCGAUAAAUUGAAGAUUUAUUUAC

>P179

AUGUAUGUUUUUAGGUGCAUAUUAUCUAAGU

>P180

GACAGAUAAGGUUCUUUACAAGAGAAACGGA

>P181

GAGAAGCAGGAUGAAUUGAUUAAAAGACGUC

>P182

ACAUCCUUUCGACUGUAACGGACCUACCCAG

>P183

CAAGAGUAGACCAUGUAAGAUUGUCGACAUG

>P184

GGCAAAUCCGGCGCUUCCCCCUCAAAAAAAA

>P185

CGGGUGUAUACAGAAUAGCAGAAUGGGCAGA

>P186

CCUAUGGGCGGGUUAUCAGAUAUUAUCAGGU

>P187

CACUUUCGGUUUUGAUCCGAACAACCCCGGU

>P188

UUUACAAGUAUCCUAUAAUCCCAAAGUGAUA

>P189

UUACGGUGACGAGUUUGAGGCUCUAUAUACC

>P190

GUCGCCAUCUUAGUAUAGUGGUUAGUACACA

>P191

UAGUCGGUUAUGGCAUCUGCUUAACACGCAG

>P192

AAUUACUCAACAUGAUAAGAAUACCAAUGUU

>P193

UUUAUACGAAAUGAAUGGAUUAACAGCAAGC

>P194

UGGGUUACAGAAGUUUUAGGCAAUGAUAUAC

>P195

UUGAGCAUAUCAAUGUAGAUGUUAAGACGAU

>P196

GGCUAAGGAUGACGCUGUUAAGCAAUUAACU

>P197

GCAUAAGGUCAGUUGUAGCACUUGUCACGGA

>P198

UGCGCUGGACCCUUGUAGUACCUGUUCAGGG

>P199

UGUUGAUCCAAAGUUUAGAAGAAACCACAAG

>P200

AAUAAUAGCAGUUUUUUAGAUAUUGACGAUG

>P201

CAUAGGUGAUAAGCAUGCGCAUAUAUAUACA

>P202

AGCUCUACAAAAGUUUGAGGCGUUGCUAAAG

>P203

UUCGUACUUGAGUUAUUGGAUAUAUGAAGCA

>P204

ACGUCGUUCGAAGAAUUUGUCGAAGAUAAAC

>P205

ACCGUGGAGCUAAAGUACUGGUACAACUUGG

>P206

ACGAAAAAGAAUCUUUAGUCGUUAAGUUCGU

>P207

CGGACGAAAACACUGUAAUUGCAAUAGUUAG

>P208

AUUAACUUGCACCCAUCCAAGCUUGUUAUCA

>P209

GGCGUCAGGUCGAGGUCCUGAUCUCUUCGGA

>P210

ACCCCUACCGGGGGAUAAUCGAGUCACUUAA

>P211

UACAUUUAUUAUUUAUUCGAUAAUACCCUGC

>P212

CCCGGGCGGUUAGUGUAGUGGUUAUCAUCCC

>P213

CUUUAGUGCCCCUUGUAGGAGUAUGGUAAAA

>P214

GGGAAUAGACACCUGUAGUUUAAUUGACUCU

>P215

GGUCACUGGUAUGGUUCUACCCCAAAAUCCA

>P216

GCUUUACGGUUGGAAUUGCAGAAUAAUGAAC

>P217

AUCUGUCGUCAAGAAUCGCAGAAAUAGCAGA

>P218

UUACGGUCUGAUUAUUGCCAUUGUCUUUUCU

>P219

UUAUUUGCAUAAGAAUUGCAGAACGAAAGCU

>P220

GCCAUAAUGAAGUAUUUGUUCACGCAAGCAC

>P221

UUAGGGCCACAUCUGUAGUCUUACCUCCAAG

>P222

AGUCAAAUGAAAGUUUAGACGAUAAUGAAGA

>P223

UUAAGAAACAAACUGUAAAAGCCCCAAAGAA

>P224

CCCAAUAAGUGAUUCUCGAGACGAAUUGAUC

>P225

CGAUCGUCUAGUAACUCUGACCGGAUCGAUG

>P226

UAAAUCAUUUCAUCAUAACCCAUAGCUGAAU

>P227

UGAGUGAUGAGGAGCUUGCUCUUUUAAGCUC

>P228

GUGUCACGGGCUCUGUUUACUACUACACAUA

>P229

AGUCAAUCACGAUUGUAGACGUUACUAAUAA

>P230

CAGCAAUUAAAAACAUUCCAAUAGCCGGUAG

>P231

GUCCUACCUCCGUUGUAGGCAGAAGCGUCGU

>P232

GAAGAUCGAGCUAUUUAUUUGAACACUCGGU

>P233

UGGCCCCAAUGGACUUUGCUAUUGUUGUCGG

>P234

CUACCACUUACCAUGUAGUAUCAUUUUUCGC

>P235

UCGAGAAGAGCUGGCUACCUUAUUGAAACUU

>P236

GGGAAAAACAGAGAUUUCACAGAUGUUACUA

>P237

UCUUCGAUUCGUUUUUAAUGAAAGGGAGAAG

>P238

GAGAGCGUAGAGCUUUCAGUGGACUGCCCUU

>P239

AUUAUAGGUUUUGAUUGACGGUCCAAAAGCU

>P240

UUUAUCUGCCCCAGUUGCUGUGACUGUCACG

>P241

GAACUUGCCCAUCGUUUGCGACACGAUCACU

>P242

CGGUUCCGAAUAGUGUAACUGUUGGUGCUGA

>P243

GUCUUUUUGACUAAUUUUCAACCUAUUCUUA

>P244

UGCGUACCUGUUGAAUUGAAGAGGUCGGAAA

>P245

CAGUGGCGGCCGCUUUGUUGUGAAGGACCUA

>P246

CAAAGAAUGGUAUGGUUGUGGGGCUUGGGGC

>P247

UGAUGGGUCUAUACCUACCAGAUGGUGGUCA

>P248

UUCCUUCUUCUAGUGUAACCUUUACGAAAGA

>P249

CGUCGUGAGACAGGUUAGUUUUACCCUACUG

>P250

CACUAAUAGGGAACGUGAGCUGGGUUUAGAC

>P251

AAGACCCUGUUGAGCUUGACUCUAGUUUGAC

>P252

UGUCCCUAUCUACUAUCUAGCGAAACCACAG

>P253

GAGUAACUAUGACUCUCUUAAGGUAGCCAAA

>P254

CAAGGGGAAUCUGACUGUCUAAUUAAAACAU

>P255

CCUAUUCUCAAACUUUAAAUAUGUAAGAAGU

>P256

AUGAGGUAAAGCGAAUGAUUAGAGGUUCCGG

>P257

AUGUCUUCGGAUGGAUUUGAGUAAGAGCAUA

>P258

UCUUUGAACGCACAUUGCGCCCCUUGGUAUU

>P259

GCGGCUUAAUUUGACUCAACACGGGGAAACU

>P260

AGAUUAGGCUCGAGCUCCCAAAGCUUUAAGU

>P261

UUAACCUCUAAAAUCUCUGAUAUCUUCGGAU

>P262

UGUCGCCGCUGGUUGUAACCCUAUGGAUUUG

>P263

UUAUAUACCUUAGUUUCACAACAACAUAAGG

>P264

GGCUACUCCAGUUUCUAAGAUGAAGGAAGUU

>P265

GGACGGUAUCCACUGUAAUUUGACUCUAUUA

>P266

UGUGGAAAUGCUUCUUACACGGUUAUGGAUU

>P267

GUAUUUUCCAAGACAUGGAUCGAAAUCUCCG

>P268

CAUUGUUCCCAAGUUUAAGGCGCAUGUAAUU

>P269

UUUCAUUCUAUGGUGUAGCGUGAGAUCGAGG

>P270

CCUUUGGUGAUAAUGUAGUCGGUGUUGAUUA

>P271

ACCAUUUGAAUGAUUUUGCUCAUAUUGAAGA

>P272

AGAGAUUGAUAUGCUUGCGACGAAACUUCAA

>P273

UACUGUCUUGCCAUGUAACUGGACUCCAGGU

>P274

UGAUUUCUUGGGCUGUGUUGGUUGCGCCAUU

>P275

GAAAGUGAGUGGAUCUUCCCUGGAAGUACGA

>P276

GGAUUCCAUGUAUGUUGCCAUUCGGGUUUUU

>P277

CCAAGGCUCCACUUCUUCCAUUGAUGAAGCA

>P278

AGGACUGCACCGAAAUAUUUCAAUCAAAAUC

>P279

CGUUAUGGGAUUAUGUAGAUGAGAACACUAU

>P280

UCGACUGUAGCGGUAUUUCAGCAAACGGUAC

>P281

CCAACUGCAAGGCCAUUACCGAUGCCGCUCC

>P282

GUUAUUGAACAUAACUGGUGUGGCCGGUUUC

>P283

UUGAUUAAUGCUGCAUUUACGUACCAAGGUA

>P284

UAAAAUCCUCAUACUUACCUUAAGAUAUCAG

>P285

AAGGAGUUGGUAAUGUAGAGGCCCAGAGGAU

>P286

AACCCUGGAGGAGUUUCAGGCAAGAAAGGAA

>P287

AUAUAAGGAAAAGUUUCAGGCUUUAAAAAUA

>P288

GUAGUCCCUAGAACAUUCGUUGGUGCUGUGA

>P289

AACGGAGGCCUUUGGUAACAACCCAAAAUUC

>P290

GGGGGACAAAAAGGUUUGUUGACAGACAAGC

>P291

CUGAUAAUGAAUGUGUAGCAGCCUGGGGUGU

>P292

GAUGGAAUUCUUGUAUUAGAACAUAAAUCUG

>P293

UACUAGCGUAAAUGCUCAAAACGCAUCCAAC

>P294

UUAGACCAUUUGGUGUGUCGACGCUGAUUGC

>P295

GCAGAUUUUUCGUGAUUGAAUCAAACAAAGA

>P296

GGGGCAGCAACUGAAUAGCAGAAGAAAUUUG

>P297

AUUGCCCCUGGUUUAUUAGAUAAAAGUCCCA

>P298

GGACCAAAACCAUGGUUGGGAGCGCAGACAG

>P299

UCACAGGUGCUUCUUUUAGUGUCUCCCAUUG

>P300

UUUACUGAAGUAUUUUGAAUCUCCGCAUAUA

>P301

GGACACUUCUGAUGUUUCAAAAGAUAUCCCA

>P302

CCCAAUGGGUUGGAAUAUGAGCAACCAACGG

>P303

CAUCAACACCGUCUAUUCUAACAAGGAAAUU

>P304

GUGGGGAUACCGUUAUUAGAUACUAAAGAUG

>P305

GAUGAUCUUGACCCAUCCUAUGUGCUUCCAU

>P306

UUCUCGGUAAACUGUUUGCCGCACAGGGUAU

>P307

AUGGAGGUGGGGAUUUUUCCCUGAGCUGGUA

>P308

UCUUAUCCGGCAUUGUUCACCCAAAUAGGGU

>P309

GGUUUGUUCGAGACCUUCAACACUGAGGACU

>P310

UGUCUUGGCCGCAUGUUACAACAAUGACGGU

>P311

UCUUUUGUGUACAAAUUUUUUAAAGGAGCGA

>P312

UCUUAAACAUUUCUGUUGGUGAAUCUGGUGA

>P313

GUCGCCGCCGAACUAUUACUCCACCUGUUUA

>P314

AUGGGAAUGAACUAUUAUCUGACUCCCACAU

**II. 314 false Ψ-site-containing sequences (negative samples)**

>N1

CACCAUAUUGAAACGUCUACAAAUGAUCGUA

>N2

CCACUCCAUCACCCAUCUCUCACCAUCAGUA

>N3

ACUCCGCUCUGCAACUCAAAGCCUCUUUUCG

>N4

CGGCGGGUUCUACGGUACCUAUAUACCACCA

>N5

AGUUUAUCUUACUUAUCUUUUCCUGGAAGAG

>N6

UUUCAAUUUUCAUUUUAAUAUUUUUUUAAUA

>N7

GGUGCUCGAGGAUGAUGAGAGGACACAACUU

>N8

AAACUAUGAUCUCAAUACAGGCAUAAAGAAU

>N9

AGAGUUGGUUAGGCAUGGUUUGUGUCCAUAU

>N10

CUAGCUUUGAAAAGGUAUACUGGAUGCCGGU

>N11

GAUAGUCACACGAAAUGAAAAAGGUGAAAAA

>N12

UCAGGAGCAGAUUUGUUCCGACGUAUCCACA

>N13

CAGACAUAAUUUCGUUUUCUUUGAGCUUAAA

>N14

AAAAUCAUCAAAAAAUUGAGUACCUAGAUCC

>N15

ACGAGCAAAGUUCCUUCAUAAACAAACCGUA

>N16

GUGGUCGCAGAACAGUUCGGAAACUACUCCA

>N17

CUUGCCAUUAUUAUGUUUUUACUCACAUAUA

>N18

UUCUUAUUCGAUAAAUUCAACCCGGAACCUG

>N19

UUGCUAUUACCGGGGUUUUCCCUUGGAUUUG

>N20

UACGACGGUUCUCAUUCGACUAUUGGAAACA

>N21

AUGGAUCUAGGGUUUUAUGCCUUAGGAUUUU

>N22

UUGUUUAUGUAUUUUUGCGAAAUAUAUAUAU

>N23

CUAGUAUUCGGACAUUUUCUUUUGUAAACCG

>N24

GUAACUUAGGUAAAUUACAGCAAAUAGAAAA

>N25

GUAUUUUAUUCAUUUUUUCUUGCUUAUCUUC

>N26

AAUGUAUCAACCAUAUAUAAUAACUUAAUAG

>N27

GAGGUUGUUUAUCUUUCGAGUACUGAAUGUU

>N28

GAAAAUGCGUGUUCCUUGUGCCAUCCGAAAG

>N29

UUUGCAAUGAUAUCCUCUUUUUCACCCGAUU

>N30

UAUAAAUACCUUAGAUGUUACCAGCUGGGAA

>N31

CAGCUGGGAAGAUUAUUACGGAACACAUUCU

>N32

AUAUUUUAUGUUAUGUUAUGUUAUGUUUGCU

>N33

UAUUCGAAAGUUUGAUUAGAGAUCACGGCCG

>N34

UUGAAUUGAACUGGUUGGCCUAUUUUCAAAG

>N35

AACUGUAUAAACAUUUUCCUUUUAUUUGGCG

>N36

AGAGGACUCAUUAGUUUCCGUUCGCGGUGAU

>N37

UUGCAAUUAGAUCUGUGGAUACAACGUCAGC

>N38

UCUCAACUGAACUCUUUGUAUGUUAGAUCGG

>N39

AGUUUCUCCGCCUCAUUCUUCAGGCCAUCUA

>N40

CUUUUGAGACGAUAUUGUUGUAGUACGUCGU

>N41

AGUACGUCGUCUUCAUCAUCAUCACUUAGUU

>N42

GAGUCAGAACCGGAAUCAUGGGCGUUCAAUU

>N43

GGUAUUCUUUGCGUCUGCGUGAAUGAAGAAG

>N44

CGCGUCGAAGAAGGAUAAGGAAAAUUUCAAG

>N45

AAAAGGUUGACCGGAUGCGUUAUCCUCUUCC

>N46

AUCCGUAUCCCAAGGUUUAUCUCUGUUAUGU

>N47

AUAUCCGGCUUUUCCUUAUAGGGAAUAGUGG

>N48

GAAUGGUACUGCAUAUUUUUAUUAUUUUUUU

>N49

GGCCAGCGCUGGAUCUAAAGAUGGCGUCGCC

>N50

GCGGUUAAAUCUGGAUUUUUUGCCACCUUUU

>N51

UAAGAACUAGGUAAAUAGGAAGGAAUGGUUG

>N52

UAAUCUCACCACUACUGUAUACCCGCUCCUU

>N53

CCGACCAAAAUAAGGUCCCUGAAAAUGAAGC

>N54

AAUUGGCCUCGUUAUUGGGCUAUGACACGUA

>N55

ACUUUGUUGGUUGGAUUUAUUUCGAUUUACA

>N56

UAAGACAAUUACAUUUUGGGUUAUUUGAUAU

>N57

AAAGGGGCGAAAUGCUUGUCCCAUACUUUGG

>N58

UCUUAAUGCCUUAAGUCAAAAAAUAGUUUUU

>N59

UGUGAUCAAAUGCUUUGCAAAAGGGUAAAAC

>N60

AGGGUAAAACAGCGUUUACAAUAGGAUAGCC

>N61

UUUUGUUAAAUAUUAUGACAUAGAAGAAAGU

>N62

UACAGUAAGGAGGUCUACAGAUGGACAUCUU

>N63

CAGGGUUUUGGAUAGUUUUUUGUUUUCCAAU

>N64

AUUUGUUGAUUUGUUUAUUUAUUUGUUGGUU

>N65

AAGUUGCAUCCAAACUCUGUCCUGUCAACUC

>N66

GGAAAGCUGAUACAGUGUUGCAUGAUGGUGC

>N67

AUUUAGAAAGCGAGUUGAAUGCCAUGUAUAG

>N68

UAGAACAAUUUUUAUUCCCGUUUUACUGAUC

>N69

AAGCUUCCAGUCCUCUCUAAUAGCCACCUUU

>N70

UUAUCAGGGGGAUCUUCAAAGAAAGGAACUU

>N71

CGACAAAGUUUUAGUUAUCCCCUUCUACGUG

>N72

UCCCAGAUGCCAUUAUUAACCUUGACGAUGC

>N73

CUCCUGAUUUGGAGGUUAUGGUAGAUAAGGG

>N74

UUCAUUUUAACCUUGUAUUGUAUUUCCUUUG

>N75

UGGGACCGGACCCUCUUGCGUAAAGUAUAUA

>N76

AAUAUUCCCCUAGUUUUCUUCUCCUCCUUUA

>N77

CCUCUUUUCUUUAUUUCAUUCUCACCGCUAA

>N78

AGAUUAUAAAGGAUAUUGUCAUAUCGGAUAC

>N79

UCUUUGAAGGUUCGUUCAAAGAUAUCAUGUU

>N80

UUUUUGGUACAUGCCUUGUUGCUAUGUUAUU

>N81

UCUCCACUUGCCCAUUCUUCAGUUUGUUCCU

>N82

AUCCCGACAUGAAGCUCUCCAUUACAUCACU

>N83

UAAUUCCACCACUACUAACUUUUGUUCUGGA

>N84

UAGUGGGUGUCUUGGUAUGGGAGGGCAUGGU

>N85

GAGUAAAUACAGCCAUCUUUUAUUACGGUAA

>N86

UGUUUUUGAAAAUCUUCAAGCUUGGGAACCC

>N87

CAUUGAAGGAGUCGGUGGCCAACUUAACGAC

>N88

AAGGUCUUCCUAUGAUUCCGGCGUUCGUCAC

>N89

GUUGGACCAAGGGUUUCCGCAUCGCGGACAC

>N90

ACAGCCUCGCCCUCUUCCGUUGGUUGUUUUG

>N91

GCGAAUUCUAAUCAAUCAAGAGUAUGGUGCU

>N92

GGCGUUAUAGAUGUCUACGAUUUCAGUGGGU

>N93

CUUCAAGAUAACGAAUUACAAAUGUGGGACU

>N94

UACGUAAGCAAAGUUUUAUGUAACAAAAAAA

>N95

CUUCAGCCUCUCUUCUACCUCUCUUGACAUC

>N96

UGGAAUCACUAAUAAUAAACCACGCAAUAAG

>N97

ACGCAAUAAGAAAAAUCAUCACAUCUUUCAA

>N98

CGCACAAUACAACAAUUAAUAAUCUAAGACA

>N99

CCUUGGCGGCCAAAGUACCUUCUGCAACAUU

>N100

CUGCAACAUUGACGGUAGCGGCUUCGUUAGC

>N101

AAGGAAAGGAGGCAAUGAGAACUUGGGUCUU

>N102

AGAAAGACCGCGUUUUCUAAGGAAUUGGACC

>N103

AUCGUACCACCCAGGUCCAAAAGAUCACAGC

>N104

AUUGGGAUUUCAAUUUAUGACCCCAGGGAAA

>N105

AGGAAGGAAAGAGUCUGCCAAACGGAAUUCG

>N106

ACGGAAUUCGGUGGGUUAACGUAUUUCGGAA

>N107

GCGCAAGGGGGGUAGUAAGGCAGUAGUAGAU

>N108

GUUAGCUCCCUUGAUUCAGCUUAUCAAAGCA

>N109

CAGCCGUUCCCGCCGUAGCGGCGGAGGCUCU

>N110

CAACUCGCUGACUAGUGCCGAUAGAUUGUAU

>N111

AAACCCUUUCGGUGAUCAUAACAAAAUUUGA

>N112

GUGUCCACCACACCUUGCACAACAUAUCUCC

>N113

UACCUCUUCGUAGAAUGCGGGCCAUCCACAA

>N114

UAUCCACGCUGGAGGUGGAAGAAACCGAUCC

>N115

AUAGUUUCUGUCCCGUAAGCCAUAGCAGCAA

>N116

GUGCCACCAGGGAUAUACAAACCAACUUUUU

>N117

GCAAAGUAGAACAAAUCGGCAGCCUCCCAAG

>N118

GACAUGGUCUCCAAGUGGCAAAAUCCAACGU

>N119

UGUUUUACCAUGAAUUGAUUGGAGAAAACAC

>N120

UCCUCUUUGGAGAACUGGAGAAUCUCUUCAU

>N121

UUCCGAUGAGGAAUCUUGUGGUUUUGUCAAA

>N122

CAUGGGUGUGGGGGAUUUAGGAUCAUCCAUG

>N123

AUCUCAGAGGCUGAGUCUCAUUUUUCUCAAG

>N124

GGUGGACAAACUCAAUCCAACCAACAGCAAU

>N125

UAUGUUGAUAUAGGAUACUUCAGUCUACGAA

>N126

CAAAUUAGCCCCCAAUCCCAAUAGUUCGAGG

>N127

AGGCAAAAUUGCAUAUGCGGAUGAAAUCAAA

>N128

GGAUAGAGUUAAAAAUAAACGAGCACUGGAG

>N129

GACAUAUGGCGAGGCUUUAGUACCACCACCG

>N130

UCUAUAUUAGCGUGCUUCUUCUAUAAAGAUU

>N131

UUGCCACAGCUCUUUUCAUGGAACAUUGGGC

>N132

GACCUGCUAGAAACUUCUUGUGGGUCUCCUC

>N133

CAUUUCACGAGGAAAUCAAAACACUUUACUA

>N134

UGGAAGCAAUACGGGUUGAAAAACCUGGUGU

>N135

AAAAAAAAACUCCGAUACGGGGAGUCGAACC

>N136

GUUGGAAUGGAACCCUAAAAUAUUUCCUCUU

>N137

AAAGUUGCAUUUGCCUUAGCCGUCCUGACAC

>N138

UUAUUACAGCCCUCUUGACCUCUAAUCAUGA

>N139

CCACACACUUUAACAUCAGAAGAAAACUUUU

>N140

ACCCCAUAUCAUUAAUAAAGAGAGUACCGAA

>N141

CUAAUAUUAUUGCCUUAUUAAAAAUGGAAUC

>N142

CUUUUUUUAUGAUAUUUGUACAUAAACUUUA

>N143

UCCUUCUUACAUAGAUGGCACCUAUUCCCUU

>N144

UUCUAGUGGUGAUGAUGUGUUUCUUAGUCUU

>N145

UGGGGUCGGCAUUGAUCAUCUUUGCUACGUG

>N146

GUUUAUUUUUCACUUUUUUCUGGUUAUACUA

>N147

CAAUAAAUCGGAAAAUUGCGACUAUGACCCC

>N148

CUAUGACCCCCCUUAUGCUGGAGAUUUGGCU

>N149

UUCCCCACUUCAUUCUUAUAGUACCAGAGUA

>N150

UACCAGAGUAUUUACUCCAUCGGGAAAAAUC

>N151

UGAAAAUAAGAGAAAUUUAUUAGUCAUAUUC

>N152

GUUGAAAGUGGAGCGUCAUUGCUAUAUAGAU

>N153

UGGCGUGUUAAAAAAUGUUUUUGGAAGUAAA

>N154

UUCAGUAGUAAGGGAUACAAAAUAUGUCUCC

>N155

AGCGAAAGAAAUAACUAAACCAAAAAAUUUU

>N156

CAAUACUGAGGAAGAUGCGGCAAUUAUAAAU

>N157

AAUUAUAAAUAAAAGUCAGGAUGAUGCCAAG

>N158

UAUUGAAAAAAGUGAUGACAAAUUCUUCGAA

>N159

UAGAAAAUACAAAUAUACUGGAGAGGGAUCU

>N160

AAGUAAAACUGAAUAUAUUAGAUACCUUUAC

>N161

CCCAGAGGAGCAAUUUGAGAUAGAACAAACU

>N162

AACUAUUAAAGUUAUUUAUAACCCGCCCGCU

>N163

CCCAAUAUCCGUCCAUAAUAAGAAAGGUGAC

>N164

AAUGGCUCGACCAACUUCAAGAAGGCAGAGA

>N165

UAGACGGGGACCAAGUCUUCGAUUUGUCUGC

>N166

UUCCAGGAAGGAGUGUCUAGUGUGGGCAAGG

>N167

AUAUACCAUUCCCCGUUCUUGUUAGGUAAAG

>N168

GUCAGGACGUCUAUCUGUGCGUUUAGCGAGU

>N169

UUCAUCGCGGCAGGAUCCCAGCAGUGGAGUA

>N170

AACUGCCCACAAAAUUUUGCCGUAACUUUUU

>N171

GGGCCAUUACGUUCCUUUGCCAGCUCCAUCC

>N172

UCAUCUUUGGAUUUAUAUUGGGUGUGUUCCA

>N173

CGAGGAACAAGAGAAUUGCCUUCCGCCUGUU

>N174

GCCAUUACCCACAAAUAAUUUAUGUGUUCCA

>N175

CUCACCAGAGAUGAAUAACAAUAAUGCAACG

>N176

UAAAAAUUGUGGGUUUUUCGUUCGCAGAGGA

>N177

GCAAAACCUACCUCUUACAGGCUUUUUUUCA

>N178

CCUCUACUGAGGAUAUUCCCACUAGAAAACU

>N179

AAAUUUUCAUUUCAGUUGUUUUUGUAACAGG

>N180

CAGGCUGUACUGAGCUGAGAGGAUCCAUUAA

>N181

GCGAACUGUUGUUUCUUACAUGACAGUACAG

>N182

UCUUGAGCCGUCAGGUCCUGCAAGGGUACCA

>N183

CCAUAAUCCACCGGUUUUAUCCCAAAAGGCG

>N184

GUAUGGUUAAAAGAAUAUAUUAUUGUAUUAA

>N185

ACCAUGUUCAGUCAAUACGCCAGGUGCUACC

>N186

CCGCUGUGCCUCCCAUUGCUGACGCCCUGAU

>N187

UCCUUUUUGGUGCGCUGAAUGUAUGCCGUAG

>N188

UUUACGUCGGAAAGAUAAAUGCCGUACCAUU

>N189

AUAAAGGCUAUAAUAUUAGGUAUACAGAAUA

>N190

UUUUAUUCCAACACAUUCGAGCUAGUAUAAA

>N191

AAAGCCUAAGAAAAUUUUGCUGGGGACAAAA

>N192

GUUUCUCUCAACCUUUGGUGACACACUAUGC

>N193

AUUUGGCCAUGAAUAUUGAAUAGUAACAAGA

>N194

UCCAUUCUUUUCCCUUUACCGCUGGCCUGUG

>N195

AACCCUCAUCCGAGAUAUACACAUUAAACGG

>N196

AGGAAUAUUAUUAACUAAGCCUUAGAACAAG

>N197

GGCGCGGUCGGGGCUUCGACUCAUUUGAUAC

>N198

UGAUGCUUUUUUCAAUCCAGCCCGCACAAAU

>N199

GAUAAAAUGACUCCAUGGCCAAGUUGGUUAA

>N200

GUUAUUGCUGGGUUGUUGUUAUUCACUUUAA

>N201

AUGACCGUAAAAAACUAAAGGCAAAAGCAUU

>N202

AUAACGAAAAAAAGCUGUAAGAAAAUGCAGU

>N203

UCAAGUCUUCAAAAAUUAUGCCGAUGGUUAG

>N204

UUAAGUCAAUCUCAGUAGCACCUCGCCUUCC

>N205

CUCGCCUUCCAUCUGUUCCCUCUACAUCUUC

>N206

GUUUUUCACCUCCACUAAUACCACGAUCAAA

>N207

UAUCUAUAGACCCCUUAGCACCACACAACAC

>N208

CAAGCAAUCGAAGGUUCUGGAAUGGCGGGAA

>N209

UCAAGGAAGUAAUUAUCUACUUUUUACAACA

>N210

GGUUCCGUUAUUUUGUUGGAAAACUUGCGUU

>N211

AACUCCUCUGGGUCCUUCUUAGCUGCAACAC

>N212

UUUUUCAUGAAAAAAUUUGGGAAAAAGAAAA

>N213

UAUUGGGAACAGGAAUACCAACCGUUCUCAU

>N214

ACGAAAUCCCCUCAGUAUAUAAUGAUCUUUU

>N215

AUAAACCACCGAAAAUAUAUUUAGGCCAUUG

>N216

GAGCAAAAGAGAAACUAGUCUCCGCAUUUGU

>N217

CACCAAUGGCAAUAAUACCUCCUAAAAGUAC

>N218

UGGGACUAGAGUCUCUAAUUUGGAAAAAACU

>N219

UCGAAGUUAAAGGACUAGAAUAGCGGGUAAU

>N220

AGCGGGUAAUCAUUAUAUCCCAUGGUAACGU

>N221

GACUACAAAGUUCCCUGUUUUGGUCGGGUCC

>N222

CACUAGUUACACGAUUCUUUGAGGGUGUAAU

>N223

AGCUGGUUUUGUUCUUAACAAUAUUUCUUGU

>N224

UCACAGAAUUUCACAUUCCCAGUUACUUUAA

>N225

AGGAGGCGGAGGGACUUUUGCCAUUUUGGGU

>N226

UAGUUUGGAAGAAUGUCUAGCAUUAGCGCAU

>N227

AAAAAUGAGAUUAGAUUAUUAAUUAUUGUGU

>N228

UGCGCAGGAAAUAAGUGCUCAAGUUUUCUAC

>N229

UAAAUGUAACGGCCGUGCUCUAGUGAAAUUU

>N230

AAAGCCUCCGUGUGGUGCACUUCCUUCUUUU

>N231

CAUGAACUGGAAUUCUACAAAGGAUCUUAAC

>N232

GGUACGUUUUCUUUUUUUCCUAUAAUAUAAA

>N233

CUAUGGGAUUGUUUGUCCACUUCAGUCUAUU

>N234

CUAUCGUCUAUCAACUAAUAGUUAUAUUAUC

>N235

AAUAUCCCCGAAGGAUAAUUAUUUCUUUGCG

>N236

GUUAUUCCAAAUGUAUUUUUCCUAUUUGCCA

>N237

UCACCACCAAAUGGAUUGUUUUCAUCCAGCU

>N238

GGAGUGUCACUAGCUUCACCCGGGGUCCAAU

>N239

GAUUCGCUUUAUUCUUUACUCCCCACUCAAC

>N240

UUUUUCCACGUAUUUUAAAAAGGGGUAGCCU

>N241

CCUUCAUACCACCAGUAACUCUGGCGAUGGU

>N242

UUUCGACUUCAGCGGUUAUUUUCUUCAGCCC

>N243

GCUCACAAAAUUACCUGCAAUUUGAGAACAC

>N244

GUAUGAUCAAUCAGCUCACGUUAUUUGAAAA

>N245

CUAAGGCUCAUUUAAUGGCCAGGCCUACAAC

>N246

UGACGGUUAGCUUAAUUAACGGCCCUGAUGU

>N247

AAAAAGUUGACGACGUCGUACUUCCACCCUG

>N248

UUGUACGUAUUCACAUGUCCUGGCGUGCGGC

>N249

UGCCCACGGCGGUGUUGCCGCAAAGGUUAAU

>N250

GACACUUUAUCCGAUUGCGGAGUCGAUAUCA

>N251

AAUCUACUGCAAUUUUUUACUUUUAGUUUGU

>N252

AAGCAGGAACACCUCUGGAGAUAAAAGCGCC

>N253

CUUGCACCAAGGGAAUAAUGAUGAUUGCUGA

>N254

UCUUUCCCUCGCUACUCACAGAUGAGCUGCG

>N255

UAUUGUUCCCAUCUAUGGCCUUUUCGUCGGA

>N256

AAAGAGGCGGUAGCCUAAAGAUACGGUAAUU

>N257

AGACGGCCAGAAACCUCCAGGCGGAGUUAAC

>N258

AUCCGCCGAUUUAUUUUUCUGUGUAAGUUGA

>N259

UUUGUUGGCCCUAGAUAAGAAUCCUAAUAUA

>N260

GGCACGCGGACAAAAUGCAGCACGGAAUAUG

>N261

CGCAACAGUAUAAUUUUAUAAACCCUGGUUU

>N262

CCAUCUUGUCUCAACUGAGAAGAAAACAUGU

>N263

CCAUAUACCCAUUCGUUGAAAAAAUUCUCUA

>N264

GAUUUUGUAGCACGAUAUCCGCAAGAAUGAU

>N265

AUUGUACUCCUCCAGUUCCUUAGCAAUUGCC

>N266

CACCAAAUACUCCUCUGCGUAUGCGAGGUUG

>N267

UACCUCUAUAUGCUGUGUAAAAUAGAAAAUA

>N268

AAGAGUUUCUUAAGAUCCGCAGACUCAAUUC

>N269

CUUUCAAAGGUCCGGUGCUGGAGAAACAGCU

>N270

UGCAGGGUGAGGGUGUCAUCGACAGCAAAUU

>N271

ACGCUGAGGAGGAGUUGGAAAGGUACAUCCG

>N272

GGUACAUCCGUGCUAUGGUCAGAGAGCAGAU

>N273

GUUCACAAGGCAGCUUCGUAUAGUAAUACAG

>N274

AUAUCCAACGGAUGCUUAUAUCCCCACGUAU

>N275

UACGGAAAGCAGCCCUCGCAGCGAUUCUGGC

>N276

GCCCAAAUGACCUAUUACUUUUAUUCAUUCU

>N277

CGGUAGUCGAUAAAUUUGUGUCAAAUUUUUU

>N278

UUUUGUUCCGGAUUUUUUUUACCGGCUCUUA

>N279

UGGUUAUAAGGGGAGUGGCAGCGGCGGUAGA

>N280

ACAUGAACCAAAACAUUUGCAUGGCCUUCCU

>N281

CCCCUCAUCUUUGAGUGAGAAGCUUAUAUGC

>N282

GGGAGAUAAUGCAACUCCACCACCAGCGCUC

>N283

AAUGAAGGAAGUAAAUAAGAGGCCGUCGCAU

>N284

CGAUGGUAAGGGUCAUUUCUGGGAGGUCAGA

>N285

UUUAUUUUUGAUCCUUACGGAUUUUUAAUAC

>N286

CCUGUACCAAUUGGAUACACUUUUGAGAUGU

>N287

AAAAUCAAAGCCAAAUGAACAAAUGGCCCAG

>N288

GGGACAUUCUAAUGAUGGAGAUGGUGUUGAA

>N289

UAAAUGCAUAAAAGAUGACAUAGAGAUAUGU

>N290

AUCGAACAUGAAGGAUGCUGUUUUUAAAAUU

>N291

GACAAACAGCCUCCGUCGAUCGAAGCAGCUA

>N292

AUGUCGUCUAAUGCUUACGUGAGAAUACCGC

>N293

CUGUGGACUACCGUGUGCGAUUGCUAUGUGA

>N294

GGAGCUAGUUCAAGUUGCUCACUGGUGGGCU

>N295

CAAAAACUUCAUUGUUCUUUUCAUCAAGGUG

>N296

GGUUAGUGAGUUUUAUGCCCCUCGUGUAGUA

>N297

UGCUACACGAAGGUCUGCUAAAACGAGACGA

>N298

UCGCGCUCGCUGACUUAUGGCGCCAAUUGGU

>N299

GCUAUUAAAGGGCAAUACCACAACCUCAAAC

>N300

CAGAGAGAAACGAUGUUGAACCCUUCUCACU

>N301

ACGUGAUACCGCCUCUUUGGAUAAUCUUGUA

>N302

UGGCACCGACGCCAUUACCACCAAUUGCGUU

>N303

AGGAUUGAGUUACAAUCUGUUUACAAUGAUG

>N304

UAUUCAGUCCCUCACUCAACUGGCGUCAAGA

>N305

AGUUUUAUAAAAUCCUCGCACUAUCGCUGUU

>N306

AAAUAAUCCUUCACAUCAUGAAAUAUAAGCU

>N307

UAGCAUAGUCGGGUUUUUCUUUUAGUUUCAG

>N308

AAUGUGGUAGGAAAAUGAAACAUAUAACGGA

>N309

ACUAAUGUUGGAAAAUAAUCCUACGAUACAG

>N310

UGGCGAUUAUAUUCAUACAAAGGUAAUGGAG

>N311

UUAGCAUACAGCAAAUAUCCCCAUUUGACGG

>N312

UAUAGGAGCCUCCGUUACUUUUGGGACAAAU

>N313

AAGGAUAUAUAAUCAUGGAAGGGUGACGCAC

>N314

UUUGUUGCAAUUUUCUCAGGGGUGGAGUGGA
