# Supplementary material for: PseUI: Pseudouridine sites identification based on RNA sequence information
Source: BMC Bioinformatics. 2018 Aug 29;19:306. doi: 10.1186/s12859-018-2321-0 (PMC6114832; doi:10.1186/s12859-018-2321-0)
Supplement: Supplementary file 3 — The benchmark dataset M_944 for M.musculus. The benchmark dataset H_990, S_628, and M_944 is formed by 495, 314 and 472 Ψ-site-containing sequences and 495, 314 and 472 false Ψ-site-containing sequences, respectively. Both H_200 and S_200 are formed by 100 Ψ-site-containing sequences and 100 false Ψ-site-containing sequences, and none of the samples included here occur in the corresponding benchmark datasets. Each of these samples for H.sapiens and M.musculus is 21-bp long with the uridine located at the center, and each of these samples for S.cerevisiae is 31-bp long with the uridine located at the center. None of the sequences included here has ≥60% pairwise sequence identity to any other in a same subset. (DOCX 54 kb) [file 12859_2018_2321_MOESM3_ESM.docx]

**The benchmark dataset M_944 for *M.musculus***. It is formed by 472 Ψ-site-containing sequences and 472 false Ψ-site-containing sequences. Each of these samples is 21-bp long with the uridine located at the center. None of the sequences included here has $\geq60\%$ pairwise sequence identity to any other in a same subset.

**I. 472 Ψ-site-containing sequences (positive samples)**

>P1

CCUAUUACUGUAGGUGUAAUC

>P2

UUUUUUUUGUUAUGGUACCGA

>P3

UUUGAACCUCUACACCAGCAU

>P4

GUUGUGCAAAUAACUUCAACG

>P5

CUGGGAUGUUUUUUAAGUUUU

>P6

AGGAUCAGAUUAAUGAUCUAA

>P7

UACACACAUAUAUGCACUCAC

>P8

GUUGGUGGUAUUAAAGAAGAC

>P9

AAGAUAAGUUUUUUUUUCUGA

>P10

CCGGAAAUAUUCAAGAACGAG

>P11

UCUACUUCCCUUUUUAGGUUU

>P12

GUAAGGUACAUAAAGAUCCAA

>P13

CAGCGCUGUGUAUGCCGGCAC

>P14

AUUUUAUUUUUUUUGUUUCAU

>P15

AGAUAUCAAGUAUUUGGAAUU

>P16

GAUCGCUGGUUCAGUCCAGUC

>P17

AUCUUUAAUUUAAUUUUUUUU

>P18

GUCUCUGGAUUUAUGGCUUUU

>P19

CCUGUUCCCUUAUAAACAGGC

>P20

UCAUUUUAAAUACAUGGGAAA

>P21

GGAGGCUGCUUUAGCGUCUUA

>P22

UUAACAAAUUUAUCAAUUCCC

>P23

CUUGAACUGGUUUGGAAUUUU

>P24

AGCGUGGCGCUUCGGCGGGCA

>P25

GUCCCUCUCCUUCAUUUCAGC

>P26

UAAAUGGUUCUUUGACCUCCU

>P27

CGAUCGCCGCUGCUCCGCCCU

>P28

AGGAACUGCCUGCACGACGGU

>P29

AGUAUUAAUAUAUAUGAACAU

>P30

AAAAGAUGUGUUCUCAGAACG

>P31

CUUUUUCCCCUUGGUACCUAU

>P32

CGCUAAUAUUUUUUUUAUUAA

>P33

UCUUUUUAUUUGUUUGUUUUG

>P34

GGUGUUUCUGUAGGAUCUUAG

>P35

GCCUCCAAAGUAUAAACAGAG

>P36

AAAUACACAGUAUGUUUAUAA

>P37

CCGAGGCUUCUACACAUCUCU

>P38

UCAGUUUGACUAUUGUUUAAA

>P39

GUGUGGAGAUUAUCAUAGCAU

>P40

AUUCACCCGGUAACCUCCAGC

>P41

CCGAGAGCUCUUUAACCCCUA

>P42

AUUGAUGAUAUUAGGACUUGA

>P43

AAUAGCCAGCUAACUGUAAGG

>P44

UAUCCUAUUUUUCCCUACAUC

>P45

UGGGGAUGUGUUCAAAAUGUA

>P46

UUCAAUUUAAUAUUGAGUUGC

>P47

UCAAAUGAAUUUACAUGAAUA

>P48

CUAGUGAGUUUACACAGGAAU

>P49

UGUGCAUUUGUCUUUGUUAAA

>P50

CCCAAGUCAGUAGAUGGUUGU

>P51

GCAUCACAGUUACGGAGGAUG

>P52

AAGGACGUUGUAGUGGAAUUU

>P53

AAGUCCAAAUUUCUACCUCUC

>P54

GAAUGACAUGUUUUCAGCCAU

>P55

ACUUAAAACUUUCAAAUAAUG

>P56

UUCUUUGUCAUAUAUACCACU

>P57

AUGGCCAGGCUUGAUUUCAAC

>P58

CUGGGUUUUCUUUAUCCUGCC

>P59

GGGAGGUGACUCCCUCCCUUC

>P60

AGAGCGUCCUUAGCGAGCCUC

>P61

AUGAUGAUGGUUCAGUCUCUU

>P62

AUCUAUGAGUUCAAAGCCAGC

>P63

CCAAGGUUUGUAUCCAUUAUG

>P64

ACAAAGGGACUUAAUACUGAA

>P65

GUCAGCCAUUUUUAAUGAUAA

>P66

AGCAGCAUCAUAUUAUCCUAG

>P67

UGAUGCCCGCUAUGAAGCUGC

>P68

UCUUAUUGUAUAGGCUUCUUU

>P69

CCGCGACUGUUAACGUGAACU

>P70

UCCUUCGCUCUAGAGGUCUUC

>P71

ACUAUUGGGUUACAGCGUGAG

>P72

UUCCUCCAUGUUCUUUCUCUU

>P73

GAUCCCUGCCUAUCAAGUGUU

>P74

CAUUGAGUGGUUUCACUUUGC

>P75

GCACCUAACCUUCAAGAGACA

>P76

CCGCUACAUGUUGACAGCGGG

>P77

GAGGAAGCUCUUCAACUCUAC

>P78

UGUUCCUGGCUCAAGUUCAGG

>P79

UAUAUGAAUGUUUUAGCAGUG

>P80

CCUGAGCUUGUUUAUAUUUCA

>P81

UUUUCUUGGUUAUUUUUUGCA

>P82

UGUGGCAUACUGAUGACCUGC

>P83

ACAGAAAAUGUAGAGUGUAAC

>P84

AGAAUGAGCCUAUCUGUCCUU

>P85

AUCUACAUCUUAUAUUACUGC

>P86

UAAGAAUGACUACCAAGAGGA

>P87

GCGGGCCGAGUUCGGGCCUCC

>P88

UUAUGAACUCUUCUGACGUCA

>P89

AAUAUGCAGAUAUACCAAAAA

>P90

UUGUUUUUAAUUAAGGACAGU

>P91

GAAGCAUGGAUACAGCUUGAU

>P92

UACAUUAGACUUUUUUAAUUA

>P93

UAAACUAAAUUAUGAAUGAAU

>P94

CCUGGAUUAAUACUGGUUGGG

>P95

UAAGAAAGAUUUAUAUAAUGU

>P96

GGGCUCAGGUUACACCUUCCC

>P97

ACCUAGGUUGUUUCCCUCUGC

>P98

UAAAUGAGAAUACUACUUUAG

>P99

AACUUGAACUUUCCUGCUUGC

>P100

UGGUGCUGACUACUUGAAAGG

>P101

AAGGGACAAAUUUCAGACUAC

>P102

GGAAAACCCCUUCUAUCUGCU

>P103

UUUAAACCGUUCCAUUUUAUU

>P104

GUGUUCUCUUUUCUUUGGGUG

>P105

UUGACCCCCCUACCCACGCGC

>P106

GGAGAUGACUUUUAAGCACCC

>P107

UGCCUCUUUCUAGGUUCUGCA

>P108

AAAAACUCAUUAUUGAAUCCA

>P109

CAGACCUCUCUUUCAAUGAGU

>P110

UCUGAAGUUCUUCUUAAGCCA

>P111

AGGAGACCGGUUCUUGAAUUU

>P112

UUUUCUAAUAUAUAAAAUACU

>P113

CAGCCUGGCCUACAUUUCAAA

>P114

CCUGGUGGUCUAGUGGUUAGG

>P115

GGUCCCCGGUUCGAAACCGGG

>P116

ACAGGGGACCUUCCUACGGCC

>P117

UGCUCCAUUCUAGCCAUUUCC

>P118

GAGCGACUCAUACCCCCGCUU

>P119

ACUACCUGCCUAUCUCCAGCA

>P120

ACGAGUGAGUUUAAAAUUAUU

>P121

UGGAAUUGGCUAUCCAUACCC

>P122

AUAUUGGAGUUCCCUUAGCAG

>P123

AAGAUUGAUGUAGAUAUUCCC

>P124

GGGGUAAAGCUUUGUACUAGC

>P125

CCCGACCCUUUACCGAUGCAA

>P126

CCUUGAGUUAUAUGCUCGAGU

>P127

GUGCUUCUGCUAAACAAGACU

>P128

AUGUUUAUGUUAGCAACUGGA

>P129

UAGAGAAUUAUUUAUUUCUUU

>P130

AGGCCCUGGCUUAUGCUAGGG

>P131

UAGCAGAACUUACCUGUGCAG

>P132

GUCAGGGUGGUUCGCGCUGGG

>P133

UUCCUGGCUUUUCCUCCUUCA

>P134

AAAGAGAAGCUAUCAAUGGGC

>P135

UUUUACCAAAUAUAUACUUUU

>P136

AAACUGUCUGUAUCAGGAUGU

>P137

CCGAUUGCUCUUCCAAAGGUC

>P138

GGGGUCCAUAUACAAGUAAAA

>P139

UAUCAAGAUUUACAAACCGCA

>P140

AGAAAGUUUCUUAAAGACUCA

>P141

ACUGCAGACCUAUAAUUUAUU

>P142

GUAUAUAUUCUGUGUGGUCUC

>P143

CUGCAUUAGCUAAUGCUAGAG

>P144

CUCAGCAAGCUGUGGACGCUU

>P145

UCCCCGGCAUUUGCGUCUCUA

>P146

CCACUACCACUAUUCAGUGUG

>P147

GAGAAACCUCUAGGAGAUUAG

>P148

AGUUGGGGGUUUUACCUUGUA

>P149

AAUGCAAAUCUUUAAAAAGGA

>P150

CAUGCCCCUGUAGAAGAGUCA

>P151

GGGGAGCAGUUCAAGCCUGCC

>P152

UGGCUGGUGAUAAUGGCCCAG

>P153

GCUGGAGGGCUUCAUCAGCCG

>P154

CCUCGCUUCCUUCCCAGGAUU

>P155

ACUGCAGGCAUUCUGAGGGAC

>P156

CUUCAUUCAGUACAUUGCCAG

>P157

CGCCUUCCUCUUCAACAACGA

>P158

CCCAGCCCUGUGCCACAGCCC

>P159

AAUGUGGGGUUUGAGUCCCCG

>P160

AUUCAGAGGCUUGGGACUUUG

>P161

AUACUAAGAAUAAUAAAUAUU

>P162

AAGGAAAAGGUAACAAAUUCC

>P163

GGUCUUUCAUUUGGAGAUCCC

>P164

GAAAGAACGCUUAACAGCUCU

>P165

ACUCACACCUUACCAUCUUCC

>P166

AACUGAACAAUUCCAAGUACU

>P167

AUGAACCUAUUUUGAGUUUUU

>P168

CAAGGUCUUGUUCCUGGAAGG

>P169

AAUCGCAGCUUAUCACAAGGC

>P170

UGACCGAGAGUCCCACCAAGU

>P171

CUAUUUCAUCUCUUGAACGAU

>P172

UGUGGAUUUGUAUCAAAAAAC

>P173

CAGCAGCGGGUCCAGGGAGCU

>P174

GUGUCAGUGGUUUCCGGUCUU

>P175

UUAAUUUUUGUUUUUAAAGCC

>P176

UGGCAGAACAUUUAGAGGAGA

>P177

ACAUUGUAUGUACAAAAGUGU

>P178

CAAAGUCCUCUAUGUCAUCAC

>P179

AGGAGCAGAAUAAGGAAAGGG

>P180

GGAACUGGCUUACAGGAAGGA

>P181

AGCUCUGGAGUAUUUGCACUC

>P182

AAAUCCACUGUAUGGCAAGUC

>P183

CUGCCAGGGCUUUAUAAAGAA

>P184

ACCUGUCAGCUAACUCAGAAG

>P185

UGGAGUGGUGUUUGGUGCCAG

>P186

CUUGAAGGAUUACCGAAGCUA

>P187

AUAAAAGUUAUACUGUACCCU

>P188

AGGAGAUUCCUACUACUUCCC

>P189

CCUGCCCUCAUUUUAGUUGGC

>P190

GGAAAUAGGGUUCAACCAUGC

>P191

UGGGACUUCUUUAUGGACAGA

>P192

AGGCCGACUGUACCCAUGAGA

>P193

CGGGUUUGUGUCAUUAAAGGU

>P194

CCAAAGAGAUUAAAGACGCCA

>P195

CCAGGGACAUUUUGUAAGGAU

>P196

AACACCGUAGUAUUGUCCAAA

>P197

AGGCUGUUGUUAUUACUACAA

>P198

GUAUCCCUGGUAUCUAAGGCC

>P199

GGUUAGAGCGUGGUGCUAAUA

>P200

GUUAGCACUCUGGACUCUGAA

>P201

GGAGUGAUUGUACCCUCUGGA

>P202

CUCAGGUUUCUAGGGGACGAA

>P203

CGCACUGCACUGUCUCUCUGC

>P204

GUCCCCCCCUUACUUUUCCUC

>P205

UAAGCUCUAGUAAACAUUCUA

>P206

AGAAGCUAAGUAAAAUGGUGG

>P207

ACAAUCUACGUACCUUUCUCC

>P208

GUGCCAGGUGUUUGAUCGGUU

>P209

ACACUGGACCUACAGUGGCUG

>P210

UUUGUAAGCCUAAGGAAGUGU

>P211

AGAAAAGACAUUUGUUGCACA

>P212

ACAGCAGCUAUGUAGACUAGC

>P213

CCCGAAUACAUAAAAGGUUUC

>P214

AUUCAGUAUUUAUUAGCCCAG

>P215

UGAACAAACCUAUAGAAAUGU

>P216

CGUUAUUGGGUACAGGAUUGA

>P217

GAAGUUGUGAUACGCGGAGCG

>P218

AUAUGGGUUAUAUAUGAGGCU

>P219

CGAUCCGAGUUCAAAUCUCGG

>P220

AUCUUGUACAUACCUUUUUCC

>P221

GUUCGGUAGGUUCACGAUGAC

>P222

GAGUUCGUUUUAUUAAUAUCA

>P223

UGCACCUCCCUUAGACGCCGU

>P224

CCCAGCCAAAUCAGAUGACAC

>P225

GUACCAUUUCUUAGUGACGAG

>P226

CUGCUGGGAUUGCAGAAAAUC

>P227

UGAAGACGGGUACAGUCAAGG

>P228

UCAAAAGGCGUUAGAAUCUAU

>P229

GGCCAACCUCUAAGGAUGAUU

>P230

GAGAUAACUCUUCGCUGCCCU

>P231

AGACAAGAUCUUCAGGCAGAU

>P232

UGACCUAGACUUCAACCCCAA

>P233

CCAUCUUUGCUUUUGCCACCU

>P234

CUAAGAUGUGUGUUGGGUUGU

>P235

AAUGUGGACUUGUAUAUUUGU

>P236

CACACACACAUAUGUGUGUGU

>P237

GCUACUUCGGUGUGGAGCCAA

>P238

GGACUCUUAAUCCCAGGGUCG

>P239

AGUUGCGGGAUAUUCCUCAAG

>P240

UGGCUGUGUUUGUUGCCUCAA

>P241

UUAUGGACUAUACCCUGAAAC

>P242

AAUGUCACCAUAUUAGAGGAU

>P243

GUUCUUCAUGUAGCAGACUUG

>P244

GACUGGCUCAUAAAGCAAUAU

>P245

UAAUCACGAUUAAGUCCUGGA

>P246

CAACGUCAGUUACAACGUGGU

>P247

CCCCAUCUUUUUGGGAAAAGU

>P248

GCUUGUCCACUGACUAGGAUC

>P249

CUUCUGCUCAUCUUAUUCUGG

>P250

CGAGAAAGCCUCUGAUGGUCC

>P251

GUGGAUGCUGUAACCCCCAGC

>P252

CUGCAGAAGCUGUGUUCCAGG

>P253

CCCCAGGGAUUUUGGAUCAUC

>P254

AACCCCAACUUACAGUUUACA

>P255

CUUGAAAGGUUUUUAUCUAUG

>P256

UCAAAUUUGCUUCCUUCAUGU

>P257

GUGGCAAUAGUAAGCUUAUCC

>P258

UGACUACGGAUCAGAAGAUUC

>P259

GAUUCCAGGUUCGACUCCUGG

>P260

CUCUGAGAACUACACGGAGAA

>P261

CCAUUGCAGAUUUCAUCUUGG

>P262

GGAAAAGGGAUAGAAGAUACA

>P263

GAACCCUUAAUUAAAAAAUUG

>P264

GAUGGGAAGCUCUUCCAGUCC

>P265

ACUAUCUAGUUUCUGUAGGUU

>P266

UCGAGCAGGUUAAAUCCCUGA

>P267

CGAGCAUGCAUUGCUUGAAAG

>P268

GGAGCAGCGGUACGGGCUGGC

>P269

UAGCAACAGUUUCGCCUACAA

>P270

CCAGCUGUAUUAUGACCCUUC

>P271

UUACAGGAACUAAGUCAUCCA

>P272

AUCCCUUAUGUUCAGACAACA

>P273

UCCCCAAACCUGCUGUUCCAC

>P274

AUUAAUUUGAUAUUUUGAUAA

>P275

UAUGAUGUGGUUUAAAACUAA

>P276

GAAGAGCGCCUAAGCAGUGGC

>P277

ACCUGGUGAUUAUGCACGAGG

>P278

UCAAUCGAAAUUUAACCCGAA

>P279

AACAGGUAUAUGACAUGGUUA

>P280

CUCGUUGUGGUGUUUGGUAAG

>P281

AUCAAGAGGCUACGGUGAUAG

>P282

CAGUGCCCGCUAAAAGUCUCU

>P283

GAGAACUCCCUAUGGACCGUC

>P284

AGUGGGAACGUUAGAACUGAC

>P285

CUGCUGGCUCUAGAUGUGUAC

>P286

AGGGACGUGGUUUUGAAGGCA

>P287

UUCCUAUAUGUAGGAGUGUCU

>P288

UCUAAACAAUUAUCCUUGUUA

>P289

GCAGCGGCGGUAACCAACGCC

>P290

UGAAGAAAGUUUUUGACGUCU

>P291

CUGAUUAAGGUUCAUUUUAUU

>P292

GCGUUGGUCCUACAGCAUGGC

>P293

AAAAAACCGCUAAUGUAGCUU

>P294

ACAGCAAGCAUACCAAGAAGC

>P295

AGCUGCCAUGUACAAAGAGAU

>P296

GCCGUUCCAAUAAUGGAUUCA

>P297

GUCUACACGCUGCCUGAGUUU

>P298

GUUCUGCACCUUCAUCUCUAU

>P299

GUUCAGGUUCUACCCGUUACA

>P300

UACCGGCUUUUACGCCAGACU

>P301

GAUCAUGAAUUACUUCUUGGG

>P302

AGGGGAGUCCUACCCAAGCCU

>P303

ACGGCACCCCUGCUGAUCUCU

>P304

UCCUGGAGUCUAUCGAUGAUG

>P305

AAGAUGUCCUUUCCGGAGGGG

>P306

UCUAAGCCCCUACCCCCUCCU

>P307

AUUCGUAACUUUUAGAAUCCU

>P308

CCAUGUCCGCUAUGGAGGCGG

>P309

UUGCACCCUGUAAGAGUGGAA

>P310

AACUGGAGGUUUUAUUCCUCU

>P311

AAAGUCAGGGUUUAAAUGACA

>P312

UUCAACCGCUUCCUUAAUACA

>P313

CAAAGCCAGCUUUGUGGAUGA

>P314

UGGGGUGCCCUUGGGAGAGCU

>P315

GACAUACCUGUAGACAGCCCA

>P316

UGGCAAAUCAUCUUUCGGGAC

>P317

UGCAACUAAAUAAAAAGUAUU

>P318

UUCCCUUCCCUUAAAAUAUGA

>P319

UAACCCAUCUUUCAUCAGCGA

>P320

UUGAGAAAAAUAUCCUUGUCG

>P321

GUAUUCUUCUUCCAAAGUUCU

>P322

UAGUCGGCCAUAUCCUGCUCA

>P323

UAUCAACAUCUAUAAGACGGU

>P324

UGGGGAGUUAUUUUUUUCCUU

>P325

CUGUGCUCCAUAUUCCUGCUU

>P326

UUGGAAGGAGUUUCUGGGGAA

>P327

GAAUCUUGGUUAAACUCCUUA

>P328

AGUUCAGAGGUCAAAGGCAGU

>P329

AAAAUGUCUUUUCAGUGGUCU

>P330

GCCUGUUGGGUUUCAAUGGAG

>P331

CCGCCCCGUCUUAGGAAGGAC

>P332

GAGUGGCCACUACCAGAACCC

>P333

UCCUGGCAUAUAGGUGACAAU

>P334

AAGAGCUUGCUCAAAAGGGAC

>P335

ACACUGUCGCUGUCUUCAGAC

>P336

UCUCGGCCCUUUACGAAUGUC

>P337

UGAACUUGUAUACAUACACUA

>P338

UUUAAUGUGUUUACUCCUAGC

>P339

CAGAACGAGCUUAAAAAGAGG

>P340

ACAGUAAAGCUUUAAAACCAU

>P341

GGGUCUCUGAUACAUAAAAGC

>P342

UUGUACAGUGUAUUCGUUGUA

>P343

CAGCAAAGUGUUCAAGAGCAA

>P344

UCUCACUCUCUUCCGAGUCCA

>P345

UGGGGCGGCGUACAAACACGU

>P346

UUAACGGCAGUGAGGACUGUG

>P347

CGCUCCCAUCUACUGCAUCUG

>P348

UACCGACAUGUGUUGUUGUUG

>P349

UUUGAGUCUGUAGCUUUGGGG

>P350

CACCCCCAACUGCGGGCGCUG

>P351

CCGAAGUGUGUCCACCUCUGG

>P352

CUUCUCUCUCUCUCUCUUUUU

>P353

CAAGGAUUCAUUUCCCACCAU

>P354

CUUUUUUGACUUUGUCCCUCU

>P355

UACCAAGCGUUAUAUAGUUAU

>P356

GUUUUCAUUGUAGUAUUUUAU

>P357

AGAACCACCAUACCAGGCCUU

>P358

GAUAACUGAAUUCUGGCCAUC

>P359

AUUCUGGGGGUACCAAGUCCG

>P360

GAAACAACCUUAUCAGGCUUU

>P361

AAGAGAAAGCUUUGCCCAAUG

>P362

UUAGACCGAUUAUUGUGGUGC

>P363

UCACCAGAGAUAAAUGACAAU

>P364

CAUAAAAUGUUUUUCCUGGAA

>P365

GAGGUGGGACUAUACUACGUC

>P366

CUGUGCUGACUAUACACGUCA

>P367

CUAGUAGUGAUACAGAUGAUG

>P368

AGUGGGCUGCUAAGCCUGGAU

>P369

GUCAGCAGUGUUUGUGAUCCU

>P370

CCGGCUCCCAUUUGUCUGGUG

>P371

GGCUUUUCAUUUUAAAUCUUU

>P372

UUAUAGACAAUUCCAUGUUAC

>P373

GAGAAACCGUUUCAAAAAUAA

>P374

GGUUUCACAGUACUCAGCUGC

>P375

AUUUGAACUAUUAAAUUUUGG

>P376

GACAUUCCACUUCUGCUGAGC

>P377

UUCCUUUUGGUAAAGCAAAGA

>P378

GAUUGGCCGGUACAACCUGAC

>P379

UCCUUUUACUUACAGAAUAGU

>P380

UCGGUUCAUUUCCGGCCACCG

>P381

UUGUGGAGAAUAUGGCCGAGU

>P382

GAAUAUCUGAUAACUCCAGUG

>P383

GGUUGAGAGCUACCGGGCCAU

>P384

UGGCCUCAAAUACACUUGCAU

>P385

AUUUUCUCUUUAUAACCUUGG

>P386

ACAACUAGGGUAUUUAGCUGG

>P387

GUGCAAAAUGUACUUCAAAAA

>P388

UGAGGUGCGCUAGGAGGGAGG

>P389

GGUUGCAUUGUGUAGUGGGAC

>P390

UGGAGGGCUGUACAAAGGAGG

>P391

ACAGCGACAGUACAAAGAAGA

>P392

AGCAAUUGCCUAUGCUUUCUU

>P393

CAUUUGGUGAUUCUACCAUCA

>P394

GGCAACAAUUUUGCUCCCAAU

>P395

AUGAGAUGUUUAUAGGCCCUC

>P396

CUCUUAGGAUUACGGGUGACC

>P397

UUCAUCCAGCUAAUGAAAUGA

>P398

CUUCUGCCACUACUAUAUAGC

>P399

AGCCUCUCUGUAGGAAUAUUG

>P400

ACGACUCUCUUAGACGAUUUU

>P401

UGAGCGAACAUUAGAAGAAGA

>P402

GAUGGGACAGUAAAUGUUGAG

>P403

UGAGAAGGAUUUUAGCUAAAG

>P404

GGGAAUAUUGUAGACUUCUUU

>P405

UCCAAGAAGUUAUAACCUUCC

>P406

CAACACCUGGUAUGUUGAAAA

>P407

CUCUUCUCCUUUCUCUGUCCC

>P408

ACUCAGUCCUUAUUUUCUCUG

>P409

ACAGAAUCAGUACCAGGCCUG

>P410

UUUACAUUUUUUUUUUAAUGU

>P411

UUCCUUCCAAUACGAAGUUUG

>P412

UGAACGGGGAUCCAGCUUUUA

>P413

GAAUGGACAAUGAGACAGGCA

>P414

ACCAUGACCAUAUGCCAGUUU

>P415

UGUACACAAAUAAAAAGGCUU

>P416

GUUUGUUUUGUUCUGUUUUUU

>P417

AGCACGCUGCUACCACCACCC

>P418

GCUUAACUCUUAUUUGUGGAC

>P419

UUGGGGAUGGUAUUAAAGUGC

>P420

CACUGAGCCAUGUACGCAAAA

>P421

CUCAGUGUUGUUUCUAGGGGU

>P422

CAACAAGUAGUAAAUAGCGGC

>P423

GCCUACAGUAUCCCAGCCUUG

>P424

UGAUGAUGAAUACGACCGAAG

>P425

GACCCCCAGGUCCAAGUGGGG

>P426

ACCCCAGGAGUUCAGCUUCGG

>P427

GUUUAGGGUUUUUAUGAGUGU

>P428

AACUUCCUGAUACAGUGGGGU

>P429

GGCCGCGCUUUAUGGUGGCGU

>P430

GGAAUUGGGGUACGGAAAAAA

>P431

UUCAACAAAGUAGGCCAGCAU

>P432

CCACUAAGGGUACAUGUGCGC

>P433

UUAUCUCCCCUAUGGAGGACU

>P434

AAAAAAUACCUACAAAUUUCU

>P435

AGAGCACCGGUAUUUCAGAAA

>P436

UCCUGAAAUUUAUUUUUCUUU

>P437

UCGCCUGCUCUAGAGGAAGAA

>P438

CCUCUGGGUUUAAUGUAUUUA

>P439

AGUGAUUUCUUAAGUUCAAGU

>P440

GGUAGUGAUGUUCGAGACAUG

>P441

GCCUGGCGGCUUCUGGCGCCG

>P442

GGAGAGACAAUAAAUGUUUUA

>P443

GGUUAAAUACUUUAAAUGGGA

>P444

CCCUUAAGGAUAUGUGGCUAA

>P445

AGAGUCAAAGUAGUUGGCCGA

>P446

GGUGGGGCCGUUCGUUCUGAG

>P447

UCUUGUUUUUUAAAGAGUUGG

>P448

CGUGGUUCUCUUUGUUUUGGU

>P449

CCUGGUUUUGUUCUUUUUCAC

>P450

CAACAGAAGAUCUUCCUCCGC

>P451

UAACCAAAACUAUCCAGAAUG

>P452

ACUUUUUAUUUUAAAAACCCU

>P453

GUUUGUGAGCUACUUCUUUUU

>P454

GAUCUUUUUCUAAGAGUUUGG

>P455

GGUGCAGGAAUUCAACCGGGA

>P456

AUGUGUUAGGUGUUUAAAAAU

>P457

UUUGGAGGUGUGGCCUUGUUG

>P458

GAGCCUGCUGUAGAAAUUGAA

>P459

GCAGAAAAAAUACUAGAGUCC

>P460

GGUGUUUUGAUAUGGUAUUAA

>P461

GCUGAGGAUGUAUAUGAUGUG

>P462

AAGUCCAGGUUAUCCUGUUAC

>P463

UUGUGCAAGAUACAGCCCCAC

>P464

GUGCUGUUGGUACGGAAGGAA

>P465

CAGUGUAGGCUACUGUGAGCA

>P466

CCCCUUUGGAUUCAACCCCUC

>P467

AGUUAAUUCAUAAUUUCCUUU

>P468

AACUAAAGCGUAUUUUAACAC

>P469

GGCACCGAGUUUCCUUGGGAG

>P470

UUUUAUGUGCUUUCAAAUGCA

>P471

GUCCCAAAUGUAGUGGCCUUU

>P472

GCCACAAACUUCUAAGGAUUG

**II. 472 false Ψ-site-containing sequences (negative samples)**

>N1

UAGCCCUAGAUUCCAGGCAGA

>N2

UAGGUAAAUAUAAAAUAAUAC

>N3

UUCCAUGUAAUAGUUAGGGAA

>N4

UCAAUGGGGUUGAGACAUUCU

>N5

UGGAAACACAUUUUACCCAGA

>N6

CACACUCUCCUAAGAACAGAA

>N7

GCUCGACACUUCAAGACUAAG

>N8

ACUAGUUCACUCACUCCUCCA

>N9

ACAAGUGGACUCAAAGAUGGA

>N10

UUGUGAGCCCUGCUGCCCCCU

>N11

CUGAGGCAGGUGGAUCUCUGU

>N12

AUAACUGAAAUGUAGUACCGC

>N13

CCUCAAAGGAUCACUCUUAUA

>N14

UAUGGAUAGCUAAAACUGCCA

>N15

AGCCCCCUGAUGAACAUGAUU

>N16

GCCCUUCUUUUUGCCUGAUUG

>N17

ACAAGUUGGUUCUUGCCUAAA

>N18

AAUAUCACUAUAAGGAAAUAG

>N19

CUUUUUCACCUAGUUGUACCU

>N20

UCCGACCACCUCCCCCGCCAG

>N21

GAGAGGCUUUUGCCUCAAGAA

>N22

CAGAAGGUACUCUAUCCAAUU

>N23

GCCUUGGAGAUAUGGGUACAG

>N24

GCUGUAGAUUUACAGCAGGAG

>N25

UGAGGGCUUAUAGAACAAUCU

>N26

UUAUUUAAUCUCAUCAGUGGU

>N27

CUCACUGUUGUACUUCGGCUG

>N28

GGGUGUGAGUUGUCACAUUUU

>N29

CAUUUUCACUUAUGUAUGAAA

>N30

UUAUUCGUAUUCUUAGGGGGA

>N31

GUGAUAAAACUCCCGAGACCA

>N32

AUAUUUUGCAUGUAACACCCC

>N33

UCUUGUAAUUUUGUACAGCUA

>N34

UAUAACAAAAUACGGGGAGGG

>N35

GGAGUUUUUGUGGAAGAAUAU

>N36

GGGCACAUGCUCCCCUAUGUU

>N37

UUGAUCUUUUUAUAAACACUU

>N38

AAUGCCUGCCUCUCAUCAUUU

>N39

UAUAAAAAGUUAGAUGCUCGA

>N40

UCAUUUGAACUCAGGAAGAGA

>N41

CAAGAACACUUUGAGAACCAA

>N42

UAAAGUGACAUGGGAAACAGA

>N43

AUUCUCAGGAUCAACAAGGUG

>N44

AGGCCUUAGGUUUCACAAACC

>N45

UCCAGGGGAGUUGAUCCCAAC

>N46

GAGGGAGCUGUGAGUUGGCAC

>N47

CAAUGAAUUUUCACCUCCACC

>N48

ACUCCUGGAAUUCUGCAAGUU

>N49

GCCCCCCUUCUUUAUAAGAAG

>N50

AAUACAAAAGUGGUGCUCUGG

>N51

CUAUUUGCAAUACAGAUGAUU

>N52

GGUGCAUGCCUUUAAUCCCAG

>N53

UGUGCGUGUGUGUUCACAUGU

>N54

AAAUGACAUCUUUUUUCUCGU

>N55

AAAACUUCCCUACCCAAAUAG

>N56

CGGGUCCCCUUCCCCUUCCUU

>N57

UUAAACAAAAUUUACAUUUCU

>N58

GGGGCAUCUCUCCACCAAAUA

>N59

UAAGAACCGGUUGUUGAGAUU

>N60

CAUGCAACAAUCUCCAUUUGC

>N61

UUUCCAGGAGUAAGAGGCUGG

>N62

GCAUAGGGAGUGGGAGCAGAA

>N63

UUGCCUGAGAUGGCGAACCAA

>N64

AUGAUCAGCUUUUGGGGGAGG

>N65

AUGUGUUGAUUUAUCAACAUC

>N66

AUAGAGGCUGUAACCGCCGCU

>N67

ACCGGGGCCAUACACAUACCG

>N68

AUUUACAAAAUAAACAGCUAG

>N69

GACCCAGAUGUAGCUGUCUCU

>N70

AAAACAAUUUUCUUAAAGGAA

>N71

CAAGCCGCGCUGAGGCUGUUC

>N72

AAAACAGCACUGGGUGAGGGC

>N73

AGGAGAAAAUUAUCUGAGAGC

>N74

GGAGGUGAUGUGGUGCCUAUG

>N75

UGUACGAUGUUGGUUCCUGAU

>N76

ACUGCUCAAAUGCAAUGCAUU

>N77

GGAUCAAGUGUUACACUAAAA

>N78

CCCUAGUUUAUUAUUUUAAUG

>N79

GGAGCCGCCCUCACAUUCGCC

>N80

UCCGCCUCCCUUAUGGCCUCG

>N81

AGUCCCAAGUUCAUGGAGGUG

>N82

UACUAAUUAUUUAAACAAGUU

>N83

GGAACGCUCAUAAGUACUGUU

>N84

GGGCCAGGCCUCGCUCGAUUG

>N85

UGCUGGAGAAUGUUUCCGCGC

>N86

UUUCCGCAGCUUUUUAGGAAA

>N87

CUACGUUUACUGCCCCCCACC

>N88

UUCUACAUCCUUGGGAUCUUU

>N89

UCCGAGCAGAUGUCUGACCAC

>N90

UAUUAAGCCGUGACAUUGACA

>N91

CCGGCCGGAUUAGGCAGCCAG

>N92

CUCCUCCUCCUAGGUGCAGAG

>N93

GGGAAGGUGUUCAAGGCCCAC

>N94

CUGCGGUGGCUUCCCAGCAGC

>N95

GCCCAGCCAAUCCUGAGUUGC

>N96

UUACGGUUCGUAAUCUCGAGU

>N97

GUUGCGAUUAUUUAAGGAAAU

>N98

GGAGUCUCAUUUGCAGUACAC

>N99

CCACCUGGGCUGCAGGAGACC

>N100

AUAAUAGUAGUUAACAGCACC

>N101

GUCCAGGCAGUGGUGGCACAU

>N102

GGAGAUUUUCUGACUCUUUCU

>N103

UAGGAAAUGAUAAGCAAAGAU

>N104

AGAUUUAUUUUUCAGCCAGGC

>N105

AAUAAAAGCUUUGCCGUAGAA

>N106

CACCAGGUGCUGAUAGGAGAC

>N107

CCCCCCCCCAUUUCACAGGCC

>N108

AAGGAUUGGGUUUUUUCUAGA

>N109

AUUUGAUUUGUGCGGGUUAAA

>N110

CUGGCCACCAUGCAUAAGUUG

>N111

AAGGGGGAUAUGUUUUUUAAG

>N112

GAAAAUCCACUUUGGAUCCCG

>N113

CUGCAAAGGGUGGAUUCUCAU

>N114

AUUCGAACCCUGGCAGCAACG

>N115

AUGGGCUAAAUUUCAACAAUU

>N116

GAGAAGGAAUUUGCUAUGAAA

>N117

CAAGACGGGUUUUUUGUUUUG

>N118

AGACCCGGAUUUAAAAGCAUA

>N119

AAAAGGGAUUUAAUAAAAUAU

>N120

GGUAAACUUUUAGGCUAUUUG

>N121

CAACUGAGGCUCAGAGGCCCC

>N122

AGUUGUUUUUUUCCUAAAAAA

>N123

AUAUUUUAGCUAAUGCCUCUA

>N124

AAGGAAAGUUUAAAGUUCUGG

>N125

AGUCUGUGUUUCCGACAUGGU

>N126

UUUAUAAAAUUAUUAAAAUGA

>N127

GUGUCAUUAUUCGCACAAGAC

>N128

AAUUUGGUUAUUAGGAGGAGG

>N129

CAUGGUGCCUUCUCCAAAAUU

>N130

GAUGCUAUGAUAGUAUAUAUA

>N131

UAUGGUCACUUGAUCUUUGAC

>N132

GUGAUGUCUAUCCUUGAUAUU

>N133

CGAUUCCUGUUAUUUUGAUUU

>N134

UAUUGUUGUGUUGUAUGCAUG

>N135

UUAGGAUGAUUAGUAACACAA

>N136

AAUGAGCCACUCGAGGGGUUC

>N137

GGACGCUUUCUCUAAAUUGUU

>N138

UUAGGGAAGUUUGCACAGGUG

>N139

UAACUGGUGUUCAGUCGAGGU

>N140

AUGGCACAGGUCGAAAGGCUU

>N141

GUAGCUCUGGUUAAAAUAUCU

>N142

GGGGGGAGGCUUGUUUGCUGU

>N143

AGGUUUUGUGUAAUGCCCCUA

>N144

CCUUGUGCCCUGCCUGUCCCG

>N145

UCUUAUCCCCUACAGUCAUAA

>N146

AACUCCUGAUUCGGCGUGCAC

>N147

UUAAUGAAAAUAUCCCCGUUU

>N148

GCUAUAGACCUCUCUAAUGUU

>N149

GAGACACCAAUUCAAUGGCAU

>N150

AAGGGGACCUUGCAGUAACAG

>N151

UCUUUUAACAUUUGAAAUAUC

>N152

ACCUAUCUUGUGGGUAGUGGC

>N153

UGCAUGGGAAUGCACUAAAUA

>N154

UUGCCAAAUGUUGGGUCCGGC

>N155

UAAAAAUAAAUGACACUUCAG

>N156

CUGUAGAGUUUUUGGCUUUUU

>N157

UAAUUGUGUAUGUGUCUUUUA

>N158

CAGAAAUACCUCUCCUGGGCA

>N159

GUGCUGACUCUGGGGGCCAUA

>N160

AGGAGUAGGAUGGGGGCUGGG

>N161

CUCAGAAUCCUCGAAACGCUC

>N162

GGAAAAAUUCUUCUAAGUGGG

>N163

AAAGAAAGGGUUUAUUAAAAG

>N164

UGAAACCUGUUUGCUCAGGUU

>N165

UACUCCAAGAUGAUUUGGCGG

>N166

CCUUGAUUAAUAAGGACGCAC

>N167

UGUUUUUUCCUCUACCAGCCA

>N168

GAAAAAAAAAUGUUCAUGUCU

>N169

AAGAGUGACAUGUUAUUUUUA

>N170

UUUUGAAAAGUUUAAUUGGUA

>N171

CUUGGGACAAUAACAGACACC

>N172

UAGUUUCAGAUCGGGUUGCUU

>N173

UUGAAAGAGGUUGGUACCAAA

>N174

CCCACCCAAAUAAAAGAGCCG

>N175

UUAGUAGCUGUUUAGGGAGGG

>N176

UUAGCUUCCUUGUCCAAUGGG

>N177

AUAAACCCUUUCCUCUUCAUC

>N178

UUUGUUGUAUUUUAAAAGUGC

>N179

AGCACCCGAUUUUCUACUGGG

>N180

CCUCCUCAAGUGAUGUCCCAG

>N181

UAUUUGGAGUUUGUCUUAAGA

>N182

GACUCUGACAUUCUAGUUGGC

>N183

UAAUUAAUGUUCCACUCUACA

>N184

CGUAAAUUUAUAUGUAUACCA

>N185

UUCUCAGGACUGAAUGGGAUA

>N186

ACCCCCCUAGUUCCUGGGCCC

>N187

ACUGCUGGCAUUCCCUUUGGG

>N188

UACGGCGGCCUCCGAGCCCCC

>N189

CAGUCAAAGUUAAAAGCCACA

>N190

UGUUCUUUUUUUUUUUUUUUU

>N191

GUGCUCGAAGUGGCCACAGAA

>N192

AGCAGGGUCAUGUUCGUCAAC

>N193

UCGCUCUUGGUUUUGGCUUCC

>N194

AAAUGUUAAGUGAGGACAAGG

>N195

GUGCUAGUGAUUGAGCCACCA

>N196

AAUAGGGGUCUUAUGUUUUUA

>N197

UUAAAAUGACUCUCAAGGGCU

>N198

GUCCUGGGGCUCCAACUCAGG

>N199

GAUGUAGACCUGAGGCCUCAG

>N200

AUAAGAAAAGUUCCAAGGUAC

>N201

AGAUGGUAGAUAAAACCUAGC

>N202

AGGAAGAGGCUGAGCACAAGG

>N203

UGUGUAUAUAUAUAUAUAUAC

>N204

UUUGAGGGUGUGGCCUUGCUG

>N205

CUGGGGAGCUUAGGACUCCCU

>N206

AACUAUGUUUUAAUUGAUGAU

>N207

UGGGGGUUUUUUUGGGGGGUG

>N208

GGGGGGGGUGUACUAAGAAUU

>N209

UUUUUUUUUCUAACUUGGGCU

>N210

CGCCUUAAGAUCAACGGAAAA

>N211

GCAAAAAAUAUCAUGGCAAAG

>N212

UCUGUAAGCUUAAGGCCCAAA

>N213

CUACACUUUUUCACUGCAACC

>N214

AUGUGGUUGCUGGGAAUUGAA

>N215

GGAGGGAAGGUAGAAAAUGAG

>N216

GACGCUCAGGUGGCACGCGGC

>N217

CCCUGUUUUAUGCAAUGCUGG

>N218

AAGUGAAGCCUAAGCAGCCAG

>N219

UUUAGGUUUCUGGGUGGAGCG

>N220

GAAGGCCACUUUGGAAGAGUC

>N221

GCCCUCGGACUACACGUGGAG

>N222

UUGUGUCUGGUGUGGUUGGGA

>N223

CGGAUGGUUGUGAGCCACCAU

>N224

CUGUGGGAGGUGGGGAGAGGG

>N225

AAAAAGAGUUUGGACUACACA

>N226

CCCGGGUCACUUUGGGCUCGG

>N227

CAGGGAGGGAUACUGGCAUUG

>N228

UACUCCCACCUCCUGCUCAGA

>N229

UGAGCUAUGGUGAGUCUGGGU

>N230

AUGUAUUUUUUAUAAAGAAUA

>N231

AUUUCAGACCUUGAAGACAAG

>N232

GAUGUUCCCUUUCUUCAUAAC

>N233

UAUGGUAUAUUAUUCAUCUAU

>N234

CCCCACUGACUAGCGUUCAUG

>N235

AGCAUGAACUUGAGUGUGGUG

>N236

AACAACCAAAUAAAAGAAAAA

>N237

UUUCUUUAAAUAAUGUGUGUU

>N238

GAAAGAAUUGUAAGCCAGCUA

>N239

CACCCACCGGUACUACUGAAG

>N240

CAUGAGAUCCUGGGGACAGUC

>N241

GUAUAGCCUUUUGUAGAAGGA

>N242

UGUCAGAGACUGAGAGGAGAG

>N243

AGUUGCCACUUUAGCAUAGGC

>N244

AACAGGCUGCUUCAUGGAUUA

>N245

GUGGCUACAAUUGUUGCACUC

>N246

CAGUUCUGAGUGUCUGGAUUU

>N247

AGUCAGUAAGUCAGGUCCCAU

>N248

AAGUUGAAUGUGGAGGGGAAA

>N249

ACAGACAGGGUACUUGUUUGG

>N250

CAAACCAACCUGCCUUUUUCA

>N251

GAGGAAACACUGAGGUAACGG

>N252

AACACAAAACUACAAAAGAAC

>N253

UAUGUAGUACUGGUUGGGCCA

>N254

AAGGAUGGCAUGGAUACUAGG

>N255

CAGAUAAUCCUAGAUUGUGUU

>N256

CGAGGUCCCCUAGACACAGGC

>N257

CCUUUGCCCAUGUUAACAUUA

>N258

GUGCCUUGCCUGCGGAGUCUC

>N259

UGGAAGGAGUUACAGAGACAA

>N260

UUUGUGGUGGUGUUAAAAAGA

>N261

UCUUUCCAGAUAAAAGGUGGA

>N262

UUGCAGGGGAUGAAAAGGGCU

>N263

GACUACAGGCUCCCCUCAUUG

>N264

GCCAUUCAAUUCCUUAAUACU

>N265

GCGAAGACCAUCUCCCCCUCC

>N266

CCACCCGGCCUGAUAGUCUGG

>N267

AUCUGCACAGUCAAGAGAAGU

>N268

AAGAUGAAUGUCAAGGAUGUU

>N269

UGGUAUCAAGUCCAGUCCCAA

>N270

AUGCUAGACUUGCGAGCCUGC

>N271

AAAAAAAUGAUUGUUGUUUUU

>N272

GAAAGAUAACUACCUUGGGGA

>N273

GGCGCCCCAAUGAUUGUAUAU

>N274

UACUCACUUGUUCACCAGCAU

>N275

GAGUCAGCCCUGCAGGGCAAC

>N276

AAACAUAGAUUAUUUAUACCU

>N277

ACCUGCACUGUAUCGUGCCUC

>N278

AAUUCAGAGGUUACCACACUG

>N279

CCCUAACUCCUAAAUAUUUCU

>N280

ACUCCAACUAUGGAAGGAGGG

>N281

CUGACUGCUCUUCUGGAGGUC

>N282

UUUUUGGUCUUCAUGGGUACU

>N283

UUUCAGUAAGUAGGACACAGU

>N284

CUUAGAUAAAUAAGGUUUCAC

>N285

ACUUCCCCUCUAAUGCCCCCC

>N286

AGGCUAGAGUUAUAGGCAAGU

>N287

AUAAGGAACUUCCUUUGUGAU

>N288

UGUGGUUUGUUUGAGACAGGG

>N289

ACUGAUUCCUUCUCUAGGUGG

>N290

GAUCCAGACAUGGCCCUCCAA

>N291

CCAUGGCUGCUAAAGUAGUUA

>N292

CCUCUUCUUCUUCUCUCCCCC

>N293

CGUCAGGUAUUUUAUCAGAGC

>N294

AAGAAGAAGAUAAAGGAGGAG

>N295

AUCCAUCAGAUGGGAGAUGGU

>N296

AAAGAGAUAUUCUCUAGCAAU

>N297

CCUAUACAAGUCAAUGAAUAA

>N298

CACUGAUUCUUUAGCAGACAA

>N299

GAGCCAGUUGUGCUAUGAUCC

>N300

GGGGUGUCUUUCAAACAUAAU

>N301

GAGAUGAGGAUUGGGGUGCAU

>N302

CACACGAGGCUAGGUCUUGGG

>N303

GAGAUUGAUUUUCCUAUGGUC

>N304

CAUUAGGGGGUGUGUGACCCA

>N305

AGAGAAAGAUUAACACAUUCA

>N306

UCAGCUUUUUUCUUUUCUUUC

>N307

GCCUGCAGUUUAGGUAUGAUG

>N308

AACUAGGAGCUCUCUGGUUGA

>N309

AGUAGCCACAUUCUUCACCAA

>N310

CUUUAAAAGAUUUUUGAUUAA

>N311

ACGCCCAGCGUAAGGGCUUUA

>N312

AUAACCCACAUAACCAUACGU

>N313

GGGCUUGAAAUAGAGACCUCA

>N314

UAAUGGGUGCUGACAACAUCC

>N315

GACAAAAUACUUUGGGUCUUA

>N316

GGGAAGGCUAUAAGAUAUUAC

>N317

UAUUAUGUGGUCAAUUUUGGA

>N318

CCACAGGCCAUGGAAAAGUGU

>N319

UCUGAAACUCUAAGCCAGCCU

>N320

CAACCAGGAAUAUCUGCAGAA

>N321

AAUUAACAAAUAUAAACGUGG

>N322

UGUUACACAUUGAUAACAUCU

>N323

UCCUCAUUUAUACUGGAAGGA

>N324

AAAAAUACAAUUCCACGUCAC

>N325

GAACAUACCUUCGAAGUAUAG

>N326

GUAAACCUACUCCCCAAGACA

>N327

UUUUCUGCGGUUUAUGCUUCU

>N328

GAAGGUGCUGUAUAGGUUACU

>N329

CUUUUCCUAAUUAGUGGGUUU

>N330

GUUUGUUACUUGUAUCCCUCA

>N331

ACAAAUGCCUUUUAACCCUUU

>N332

AUUUGAUGGUUCCUGGAACCA

>N333

AAUUGGAGGAUUAGGAAUAAA

>N334

AUAUUUUAAAUAUUAAAAGAG

>N335

UUUGCUGAAGUGGAAGGCGAG

>N336

AACAUUGAAAUGGGUCUUCUC

>N337

CUCCAAAUAGUUUGGACCAGA

>N338

UUUUCUGAAGUCCACCUUCUU

>N339

AGGGGCCCAAUUCCCGCCAAA

>N340

GAAGGAGUUCUAAUACAUCAU

>N341

UGAAACACCAUGAAUUUGAAA

>N342

UCUUCUAAAGUAAGAUCCCCC

>N343

GGUAACAGAAUCACCAGGGGA

>N344

GAAAAAGGAGUGGAGAUCUGA

>N345

AAUUUAGACAUAAGACUCUCC

>N346

UUUGGAACACUGGGGAAACGA

>N347

GUGAUUGGGUUACCUCACUCA

>N348

CAGGGGCAGUUGUACCCCCUA

>N349

CAAACUACCAUUGGGGAGCUU

>N350

CUUUCCGCCUUACCACGUGGC

>N351

CUCCCGCAAAUCCAUUCUUGG

>N352

GAAAUAAUCAUACUCAGGGCU

>N353

AAUUUUAUUUUUAUUUCAAAA

>N354

AUAUCCAAAAUCUAUCCCCUC

>N355

ACGCUAAAUCUCCUUUGUUUC

>N356

AUCCCAAAAGUCCCCCAUACC

>N357

AUGAUACCACUAACAGUAUUC

>N358

GGUAGGACAGUCUCUCGGUGG

>N359

CCCCCACUAAUAGUAAUCCUC

>N360

GACAAUACCUUCUGUGUCUAA

>N361

GGGAAAAUGGUACAUAAUUUU

>N362

AAAGAGGGCAUGUAUGUGGGU

>N363

AUUCCCCUUCUAUAUCUAGAG

>N364

UCUGGAACCAUAGAUUCGAGC

>N365

UUUUGCUGCUUACAGGAAACU

>N366

CUCGGACAAAUCAAAAAGUUA

>N367

GGAUACCACUUUACCAUUAGC

>N368

CGGAAACCCCUUUCCUAUUUA

>N369

GAAGAUUAGGUUGAAAAUUAA

>N370

CCAAUGCUCAUGGACUGGCAG

>N371

AUCAAGGAACUCCACAUAAAA

>N372

GAGGUGAGGCUUGGAAGGGGG

>N373

UGAUGAUUUUUAAGGUAAUGG

>N374

CAGGCUCCUGUCUGCAGGUAU

>N375

GGGCUGUUUCUGGUUGAGAGA

>N376

UGGGCUAUCUUGUUGCCAGGU

>N377

GGUGCCCAACUAGAAACUUCA

>N378

UUGGCUUAAAUCAGUUUACAU

>N379

UAACAUCCUAUUAUGACACUG

>N380

GGGAGAGUCCUCACGUUGUGA

>N381

GGGAGGAGAAUGUUAUAGUUU

>N382

GAGUGAACAAUAAACCCAGAA

>N383

UUGAUAAACCUGGGAGGGGGA

>N384

GGAGGACAACUAGGGAGGAUU

>N385

GAGGUCAUUUUUUUUCCAAUU

>N386

CCAACCCACAUACACAGAGAG

>N387

GACUUAAAGUUAGCACACAAC

>N388

UUGUAAACUGUAAGGCUUGUA

>N389

AGUAUGAAGCUAUAAACGUUA

>N390

AGGAGGGUAAUAGAAUAAAUA

>N391

AGAACUCUGAUAUUCCACCUA

>N392

CAACAUUUAAUUGGGGCUGGC

>N393

CAUCACCAAAUGAAUUACAGA

>N394

AAAAAAGGUGUCAAACGUUUG

>N395

CAGAGGGACCUACGGCCUUUU

>N396

GCCAGAGAAGUACGACAGUGG

>N397

AUAACAGUGUUAGGAGGGGUG

>N398

GAAAAUUAAUUCUGCAGAGUC

>N399

CCAAAAGGUAUAAUCACACAU

>N400

GGGGGCGCGCUGGGAAGGCCC

>N401

UUGAGGCAGUUGCCAACAAGA

>N402

CACCUUCAGAUAACCCUGGGA

>N403

ACAAAACACAUAUUAUUUGAG

>N404

UUUGUGUGUGUGUGUGUGAUU

>N405

CAAAAGGACAUACGUGGUAUG

>N406

UACUAUAACAUAAGUAAGGUA

>N407

GGUGGAUCUCUUCACCGGAAC

>N408

AACUUCAGUAUAAUGGUUUAU

>N409

GGGGAUAGGGUAUGAGUAUAC

>N410

UUUUUUUUUUUGGGGGGGGGG

>N411

GAUAACCACAUGGCAAAAGGC

>N412

CAUGGGGAAAUAAGAAAAGAU

>N413

AUUAACCGCGUUUGGUAGAGU

>N414

AGCUGGUUCUUUGAGAAAAUC

>N415

AGCUUGUUCAUAACUUUAGAG

>N416

AAAAAAGAAAUCCAAGAAUGU

>N417

AGUUCCCUUUUGGCACAAGGG

>N418

AAACAAUGAGUUAGGGUGCUC

>N419

UGGGGGGUUCUAGGAGGAGCU

>N420

CAUGAUUUAAUAAUGCAAUAA

>N421

UCUACCCGUUUAAUUAUGAUC

>N422

GUAAAAAGGAUUAGAUAUCCA

>N423

AAUUCUGCUGUGAAUUUUCUU

>N424

UAGGCUCAUAUAUGCCACAGA

>N425

CCCGCAGUUCUGGUUCCGGAA

>N426

UUAUCUAACUUGAAGAGGGAA

>N427

CAAAUGUUUGUAGAAAGAAAG

>N428

AAGUCAGUGUUCUCACUUGGG

>N429

CGCAUAUUAAUGAGGUACUUG

>N430

UUAUUAUGUGUCGGGAGGAAU

>N431

GAUGGGACCCUGACCAAGAAG

>N432

AAUAUUAAAAUAUACAUAUUG

>N433

UUUUUCCGCAUGUAUAUUCAG

>N434

AGAGACGAGCUCUGGUGGUGG

>N435

GUGUUGGGGUUCCCUGGACUG

>N436

UGUCAGUUGGUAUUAAACUCU

>N437

CAUAGAAUGAUCUGAGUAUUG

>N438

AAAAGAAAGAUGGGCCUGGAA

>N439

GCUCAGGGGAUGAAGACAAUC

>N440

UUUCUUUGAUUGGUGGUUUAG

>N441

AGAUAUUGUGUGAAUUUGUUU

>N442

CUGAUCAAGCUGUAUUUCCCC

>N443

GAAAAAUCCGUUUUAUAUUGG

>N444

AACCCUGAAGUAACAAAUGAC

>N445

AUGUCACAAAUUGAGCAUUGU

>N446

CGCUCACGCCUCUGCGAUUAC

>N447

UAUCCAACCCUCUUUACAAAG

>N448

GACAUGCCGAUCACAGCUUUC

>N449

CUCAGCAGCAUACUAUGUUAU

>N450

AUAUGCAGUGUGGUUAUCUCA

>N451

GUGAAAUGACUUAUGGGGUGG

>N452

CUGCAGCCAAUCAGGGAGUGA

>N453

GAGUGAGACAUGAUGGAGACC

>N454

GGAAUCGGUGUGCCAGGAUAU

>N455

AUUGUUACCUUAGCUCCCCCC

>N456

GAAGAACCCAUCUGAGUUUCU

>N457

UUAAAAUUUCUACUAAGGUAU

>N458

UGGCUGUAAAUGGAGAGGAUA

>N459

AUCUGGGCAAUCCCCCAACUG

>N460

GGUCUCAGGUUUGGUUCCCAG

>N461

CAAGCAUCCUUCCCUGAGCCC

>N462

UCAUGAGAUGUGCCUUUAAAU

>N463

AGCAAGCACUUUACAUGCUGA

>N464

AGUGGGGGAAUAAAGAAGGGA

>N465

ACAAUCUAAAUAAGGGGAGUG

>N466

UAAAGUGGUUUCGUAUAUAUG

>N467

GUGGCCCCUUUCUAAAUAGUU

>N468

GCCCAGGCAAUCUUAAAGUCA

>N469

AGAUGGCAUGUUUAUAAAAAG

>N470

AUGUAAAACCUAAUAAAGACC

>N471

CUGAGGCAUUUCUUACCCUGC

>N472

AGCAUUAUUUUAUUAAAACAA
