# Supplementary material for: PseUI: Pseudouridine sites identification based on RNA sequence information
Source: BMC Bioinformatics. 2018 Aug 29;19:306. doi: 10.1186/s12859-018-2321-0 (PMC6114832; doi:10.1186/s12859-018-2321-0)
Supplement: Supplementary file 4 — The independent dataset H_200 for H.sapiens. The benchmark dataset H_990, S_628, and M_944 is formed by 495, 314 and 472 Ψ-site-containing sequences and 495, 314 and 472 false Ψ-site-containing sequences, respectively. Both H_200 and S_200 are formed by 100 Ψ-site-containing sequences and 100 false Ψ-site-containing sequences, and none of the samples included here occur in the corresponding benchmark datasets. Each of these samples for H.sapiens and M.musculus is 21-bp long with the uridine located at the center, and each of these samples for S.cerevisiae is 31-bp long with the uridine located at the center. None of the sequences included here has ≥60% pairwise sequence identity to any other in a same subset. (DOCX 26 kb) [file 12859_2018_2321_MOESM4_ESM.docx]

**The independent dataset H_200 for *H.sapiens***. It is formed by 100 Ψ -site-containing sequences and 100 false Ψ-site-containing sequences. Each sample is 21-bp long with uridine located at the center. None of the samples included here occurs in H_990 of the Additional file 1.

**I. 100** Ψ**-site-containing sequences (positive samples)**

>P1

GCUAAACAGGUACUGCUGGGC

>P2

UUAUUGAGUGUCUACUGUGUG

>P3

GAUAAACUGUUACGCAUAUAU

>P4

UUGUCGGUGUUAACAAAAUGG

>P5

UCGGGCCUAGUUCAAACCUUU

>P6

UUUUUAAGUAUACAGGGGUCU

>P7

GGCCGGUCUGUAGCGGAUCAC

>P8

UAGCUAUCGCUUCUCGGCCUU

>P9

UGAAAGUAACUUUGCCCGAGC

>P10

ACUAUUCUGUUAAAAUCAGGA

>P11

GCAGCUGCCUUUCCAACAGCC

>P12

CAAAAUGACUUUCGUUCUUCU

>P13

UUCAGAUACUUACAUAGUUUU

>P14

CCGAAUCAACUUUGCCGUGUU

>P15

GACUCAAAGUUACUCUCCUUC

>P16

CUACCCACCUUUCCCAGAAGU

>P17

GGACAAUAUAUUAAAUGGAUU

>P18

GAGGACAAUAUAUUAAAUGGA

>P19

GUGUAGUAUCUGUUCUUAUCA

>P20

AAGUGUAGUAUCUGUUCUUAU

>P21

UCAAGUGUAGUAUCUGUUCUU

>P22

AGAUCAAGUGUAGUAUCUGUU

>P23

CUUCUCGGCCUUUUGGCUAAG

>P24

AGUAAUCGCUUCUCGGCCUUU

>P25

GAGUAAUCGCUUCUCGGCCUU

>P26

GAUGUAUUGUUUGCACUCUUC

>P27

AUGAUUCUAUUAUAGUAUUCU

>P28

UGUUUUUGUAUUGUUGCUCCU

>P29

UUCUUUUUUUUGGCCUUUCUC

>P30

GCUAAACAGGUACUGCUGGGC

>P31

CCAUUAUCGCUUCUCGGCCUU

>P32

CAUUAUCGCUUCUCGGCCUUU

>P33

UGUAAUAUUUUAUCCCUGGAC

>P34

UAGUAUCUGUUCUUAUCAGUU

>P35

GUAGUAUCUGUUCUUAUCAGU

>P36

GUGUAGUAUCUGUUCUUAUCA

>P37

AAGUGUAGUAUCUGUUCUUAU

>P38

UCAAGUGUAGUAUCUGUUCUU

>P39

AGAUCAAGUGUAGUAUCUGUU

>P40

GUAUUGAGUGUCUACUGUGUG

>P41

UUUUCAUCACUAUGGCUUAGC

>P42

GCAUCAAAACUUCACUUUUUG

>P43

AUUGGUGGUAUAGUGGUGAGC

>P44

GAUAAAAGGCUAAUAUCCAGA

>P45

GGUCCCUGGUUCGAUCCCGGG

>P46

AGACUGAAGAUCUAAAGGUCC

>P47

GGGAGAGCGUUAGACUGAAGA

>P48

UGGGAGAGCGUUAGACUGAAG

>P49

AAAUCCUUUCUAAAUUGCAUG

>P50

CAUAAAAAGUUUUUUCUUCAG

>P51

AGAGUAUGGAUUCCGAUAUGA

>P52

AAGACAUGAAUAAGAACUGAU

>P53

GACUUUCAAUUAUCUGUGUGA

>P54

GCCUUUUCUUUGUUUGUAACU

>P55

AGCCAUCAGGUAAGCCAAGAU

>P56

CUUCUCGGCCUUUUGGCUAAG

>P57

AUCCAUCGCUUCUCGGCCUUU

>P58

GGGCCCAGGGUGCUGUGGAGA

>P59

AUUGUCCUCCUUCUGAAGCCC

>P60

CCUCCUUUUCUGAGGAAGGUG

>P61

AUUGGAACGAUACAGAGAAGA

>P62

AGACUAUACUUUCAGGGAUCA

>P63

GCGCCCCAAUUAUUAUGACUG

>P64

UAAGUUAUUUUGCUCUCACUG

>P65

GCAAUUUGGUUCCACCACAUC

>P66

ACUCAAUACUUACCUGGCAGG

>P67

CACUCAAUACUUACCUGGCAG

>P68

CUGGCUGCUGUAGGUCUUUUC

>P69

AUUGUUGAUAUUUGCCCAGCA

>P70

GGGCCUCAGUUAGCUCUCAAG

>P71

UCCCAUGGUGUAAUGGUUAGC

>P72

GUUAGCACUCUGGACUUUGAA

>P73

GGACUUUGAAUCCAGCGAUCC

>P74

GCGAUCCGAGUUCAAAUCUCG

>P75

CGAUCCGAGUUCAAAUCUCGG

>P76

UCAUUUUAUGUAUAUUUAUCA

>P77

CCUUUCCAGUUACUCCUUAUA

>P78

UAAGUUAUUUUGCUCUCACUG

>P79

UCAAGUGUAGUAUCUGUUCUU

>P80

GGUAGGUGAGUUUAAAGUCUU

>P81

CUCUUACCUGUUAAAAUCAGG

>P82

GCAACAGAGUUCAACUAUCUC

>P83

CAUUUGCUGUUACUCUGGAGA

>P84

UCAAGUGUAGUAUCUGUUCUU

>P85

GUAAAAGGGUUACUCUCAUAC

>P86

UUUUAUUAUUUGGAUGAAUAU

>P87

CUUCUCGGCCUUUUGGCUAAG

>P88

AACUAUCGCUUCUCGGCCUUU

>P89

AAACUAUCGCUUCUCGGCCUU

>P90

CCCUGGAGGUUCCAAUCCUGC

>P91

UUCUCCAUGAUUCGUGCAUCU

>P92

CUAAUUAUGCUGGACUGUUUU

>P93

AUUGGAACGAUACAGAGAAGA

>P94

AUAUUUCUCAUUUCUUUUAGU

>P95

UAUACUAAAAUUGGAACGAUA

>P96

AUUGGAACGAUACAGAGAAGA

>P97

ACACGCAAAUUCGUGAAGCGU

>P98

AAGUGUAGUAUCUGUUCUUAU

>P99

UCAAGUGUAGUAUCUGUUCUU

>P100

UCAAGUGUAGUAUCUGUUCUU

**II. 100 false** Ψ**-site-containing sequences (negative samples)**

>N1

GUGAUAUAACUCAGUGGCAGA

>N2

GGCCUUGGAUUUCAUCCCCAG

>N3

GGAGAGGGAGUGGGAACAGGA

>N4

UUUGCAAGACUCCUAGUACCU

>N5

UGUGUAGCAAUGGUGUCCAGG

>N6

AGUAACAAGUUCAGGUUCACC

>N7

GCAAAGUCACUCUAUUCUGAU

>N8

CCCAAAGGUUUACUUAAUGUU

>N9

UAGGUUCCUGUUGCUUGCCAU

>N10

CUAAGAGGUUUGUUGUCCUAU

>N11

UGGAAGUCUUUUCCUUUAAAG

>N12

UCUCUUAGCAUCAGACACUUA

>N13

AGAGAGAGAAUGAGAAUCAUC

>N14

GUGGAAUGAAUAGACUUAACU

>N15

GUCAGGAGGCUGUCUUACGUA

>N16

CACAAUUGCAUGUGGAAGCUG

>N17

CAAUAACUCAUUCCUACAGCC

>N18

CCACAAACGGUUUAAGCUUGA

>N19

GUCACAAUAAUCAUCAUUUCA

>N20

UUCCUUCAAAUAAAAAAAAAU

>N21

CAUUUCUGAAUUCAGAUGUAU

>N22

CUAUCAUAGUUGGGUUUAAGA

>N23

AUCAGAACAUUGGGUAUAUUC

>N24

CACCAUGGUGUCUGGGAGCAC

>N25

ACAUUACCCCUCCCUUCCCGC

>N26

ACCAACGAUCUGCUUGUGAAC

>N27

AGAGCUUUAGUCCAGAGCAAG

>N28

CCCCCGCCUUUUUUUCUGUUG

>N29

UAAAUUUUGUUAUGCAAUUAA

>N30

UUUAGAGGAAUAGGGAAAGUG

>N31

GACGUGUCUGUUGUUUCUCAA

>N32

GGGUCCGGACUGUUUGACACU

>N33

GAUGAAUGCUUUCUCAAAAGU

>N34

UUAAACAGUUUCAUUUGGAAG

>N35

UAGGGUCGCCUUAAGUCAACA

>N36

UCACAGAUGCUCCAGCAGGCA

>N37

ACCAUAUGUUUAGAAAUAAAA

>N38

CCAGCCGCGGUGCCAGCAAAG

>N39

AACAGACACAUUACUUGAACU

>N40

UGUUCUGAGUUCUACUGUCUU

>N41

ACCCAAAUGCUCGGAAACUCU

>N42

CUUAUGACUGUGACUUCAGAA

>N43

AAAGAAGGAUUCCAAAGACAA

>N44

ACUCAAAUUCUUAGAUGACCA

>N45

AGGCAGACAGUAGGAAGAGUA

>N46

AUGGAAAUCCUUUUGUUUUGU

>N47

UGUUCUGUUGUUGUCAAGUGC

>N48

AAAAAUAUAAUUUGUUGAAUA

>N49

UGUGUGCUUCUGUCCUACUAC

>N50

AUUUCUUCCAUUUUUAAUUAA

>N51

AAAGUAGAGCUAGGACCCACU

>N52

CUUGUUCCUGUACUCACUGUA

>N53

GGACCCCACCUAAAAGUAUAA

>N54

UCCUGAGAGUUCACGCUGAGC

>N55

CUUUUCUCUCUCUUCCUGAAA

>N56

ACUGAAGUGUUCCCAAAGCUA

>N57

UGUGUAAAGGUUUGGUUCUCA

>N58

UCUCUCUCUCUCUCUCUCUCU

>N59

UGUAGGUGGGUAGUAGGUGAG

>N60

CAGCUGGGAGUUAAAUACUCU

>N61

GUGGAACCUCUCUAGUUAAAA

>N62

GUAACCAGUCUGUGGGAAGUA

>N63

AAAGCAACAUUCCCUGCUGGA

>N64

GGCUCCAGGAUCCUAAGGGAC

>N65

GUCUGUACUCUAAGGGGACAU

>N66

UUAAAUUGCAUCUCCCUCAUU

>N67

AAAUGAUGACUGAUGCUACUA

>N68

UGUUUAAACAUUGGAUUUAAC

>N69

GUUUAUUUCAUUGUUUUUAUU

>N70

UCACUGUGGGUCUGGGCUUUA

>N71

AGACCCUCAUUUUAGCUGCCU

>N72

AGCCUUCAGAUGAAGGGGGGG

>N73

UCUCUGCUAAUUAUACAUCUG

>N74

GAGUUCAGCCUUCAGAACUUG

>N75

UCAGCCACCCUACCCUACUUG

>N76

GACCAUGUCUUGAAAAGACAA

>N77

GUGGUUGACUUUGGGUUUCUU

>N78

AUGUGUUUGUUUGUUUGUUUG

>N79

UUUGUUUUGCUCCUGACACCA

>N80

CCACCCUCUUUUAAGUAGAUU

>N81

GUGACCAGAAUAGUAACUAAA

>N82

AUGUUGAAUUUAUUUGCUUAA

>N83

CAAAUGUGGCUCUAAAUUUUA

>N84

AGGAUCAUUAUGAAAGAUGAA

>N85

UAGCUCCCCUUUCUCUGCUUG

>N86

UGAACACGUAUGCCAAUGGAC

>N87

UCUGCUCCCGUGUUACAGUGU

>N88

GACCUAACUUUGGAUACUUUU

>N89

UCCUCUAUAGUUAACCACAUU

>N90

AAUUUCAAAAUUGCAGAGAAA

>N91

UGGAUCACUUUGCAUCAGUAG

>N92

GGCUGGUAAAUUGAAAUACUG

>N93

GACCAUCACAUAUUUCCUGGU

>N94

GCUUCUUUGUUUAUUCAUUUG

>N95

GCUAUUCCAUUGUUCCUGUAC

>N96

CAUCAAUCUUUCUCAGUUUGU

>N97

GAACAUGAGCUCUUGAGAUUC

>N98

AUUCAGGAGGUCUCAGAACAC

>N99

UAAGGCUUUAUUGUCUCCUAA

>N100

UCUUAACUCUUGGGGCUGGUA
