# Supplementary material for: PseUI: Pseudouridine sites identification based on RNA sequence information
Source: BMC Bioinformatics. 2018 Aug 29;19:306. doi: 10.1186/s12859-018-2321-0 (PMC6114832; doi:10.1186/s12859-018-2321-0)
Supplement: Supplementary file 5 — The independent dataset S_200 for S.cerevisiae. The benchmark dataset H_990, S_628, and M_944 is formed by 495, 314 and 472 Ψ-site-containing sequences and 495, 314 and 472 false Ψ-site-containing sequences, respectively. Both H_200 and S_200 are formed by 100 Ψ-site-containing sequences and 100 false Ψ-site-containing sequences, and none of the samples included here occur in the corresponding benchmark datasets. Each of these samples for H.sapiens and M.musculus is 21-bp long with the uridine located at the center, and each of these samples for S.cerevisiae is 31-bp long with the uridine located at the center. None of the sequences included here has ≥60% pairwise sequence identity to any other in a same subset. (DOCX 25 kb) [file 12859_2018_2321_MOESM5_ESM.docx]

**The independent dataset S_200 for *S.cerevisiae*.** It is formed by 100 Ψ -site-containing sequences and 100 false Ψ -site-containing sequences. Each sample is 31-bp long with the uridine located at the center. None of the samples included here occurs in S_628 of the Additional file 2.

**I. 100** Ψ **-site- containing sequences (positive samples)**

>P1

CUAUCAUCGCUGAUCUCCCACUCCCUGAUCU

>P2

GAAGAGGUCAUCGGUUCGAUUCCGGUUGCGU

>P3

GUAAGAUGCAAGAGUUCGAAUCUCUUAGCAA

>P4

GCGAAAGAUUAGAAAUCUUUUGGGCUUUGCC

>P5

GGUUAAGGCGAAAGAUUAGAAAUCUUUUGGG

>P6

UUUAGGACCGAGCUUUUAGUGGAUGUCAUCA

>P7

GGACACUUCUGAUGUUUCAAAAGAUAUUCCA

>P8

GGUACUGGACGAGAAUCGCAGAACAAUUUGA

>P9

CGUAGAUGUUUGUUGUUCACCCACAACUGAA

>P10

GAGUUGUCGAGUUUUUUGAGGUUAAGAAUGA

>P11

AAGGUCGAAAAAGUUUCAGGCAGUUUCUCAG

>P12

CGUUGGGCCCCCGGUUCGAUUCCGGGCUUGC

>P13

UGGUAAAAUCCAACGUUGCCAUCGUUGGGCC

>P14

UAAGCGCAAGUGGUUUAGUGGUAAAAUCCAA

>P15

GGUUAAGGCGAAAGAUUAGAAAUCUUUUGGG

>P16

GCGAAAGAUUAGAAAUCUUUUGGGCUUUGCC

>P17

GGCUUCAUUAACAUGUACUUCAACUACGGAA

>P18

GUGGAGAUCAUCGGUUCAAAUCCGAUUGGAA

>P19

UUUGGUUUUCAAGUGUAAUAGGCUACGUGAU

>P20

CAGUGGUUCAAGACGUCGCCUUUACACGGCG

>P21

UAGUGGUUAUCACUUUCGGUUUUGAUCCGGA

>P22

CACUUUCGGUUUUGAUCCGGACAACCCCGGU

>P23

AAUUGAUCUAUGUUGUAGCUGCGCUGGCGGC

>P24

AACUCCAGUUCUUUAUCUUCUUUCUCCGCUG

>P25

GCGUCUGACUUCUAAUCAGAAGAUUAUGGGU

>P26

UCUUCCGUGAUAGUUUAAUGGUCAGAAUGGG

>P27

CAGAAUGGGCGCUUGUCGCGUGCCAGAUCGG

>P28

GUGCCAGAUCGGGGUUCAAUUCCCCGUCGCG

>P29

AGAAAAAGCCAAUGAUGAGAUACAAGCCAUU

>P30

AUCGACAUAUGCUGGUUACAUGGCAGUAGAA

>P31

GAAUAUACAUUCUAUUAUCGAACCUGGCCAU

>P32

GAAACAAGAUUUCUGUAGCAUACUCGCUUCA

>P33

UACUUGUUUUCUUUUUUGUGCCUUUGUUACG

>P34

UUGCUUUGUGGAAGUUCGAAACUCCAAAGUA

>P35

UGAGUGAUGGAAGUGUAGUUAUCCGGAGAUC

>P36

AGGGUCAAAUCUUCGUUGACCGUCAAUUACA

>P37

UGCAGCACAAAUUUGUAGACAGGCUGGUUUG

>P38

AGGAUUACUUGGACAUUAACGGUUCUCCUAU

>P39

UCAAGACAAAAGUGUUCUUUCAUCUGCAGUG

>P40

UUGGCGUACAGAUUGUAGUUGUGGCUGCUAC

>P41

CUUUUUUAAUGUCCGUUUCUAUGAUUGGGCU

>P42

AUUGUUCGAAGGUAAUGCCUUGAUCAGAAGA

>P43

CUGUUGGUCCUUAGUUCGAUCCUGAGUGCGA

>P44

GCAGCAGAUUGCAAAUCUGUUGGUCCUUAGU

>P45

UUAUCCGAUAUAGUGUAACGGCUAUCACAUC

>P46

CGUGGAGACCGGGGUUCGACUCCCCGUAUCG

>P47

GGUAUGUUAUUUAUGUAACGGGUAUGCGAAC

>P48

AUUCUUUUUUUGAUGUAAUAGGAUAAGCUUG

>P49

CUGUUCUUUUCAGUGUAACAACUGAAAUGAC

>P50

UGUAGUAUCUGUUCUUUUCAGUGUAACAACU

>P51

GUGUAGUAUCUGUUCUUUUCAGUGUAACAAC

>P52

AAGUGUAGUAUCUGUUCUUUUCAGUGUAACA

>P53

CAAGUGUAGUAUCUGUUCUUUUCAGUGUAAC

>P54

AUCAAGUGUAGUAUCUGUUCUUUUCAGUGUA

>P55

UCAUUGUUCUUGGAUUUCAAUGGGUGCUGUC

>P56

UAAAUUUCGCCACUGUAGAUGAAGAAGACGA

>P57

AAAAUGAGAAGAGUGUAGAUGUAUUAUCCUU

>P58

CCAAGAUAGACUAUGUAAUGGUAAAGAACAU

>P59

AUGGCGGCGGGUGCCUUUGGAGCAGCAAUCG

>P60

AUGGUGUGGUCACUGUAAGAGAUUGGCCCCA

>P61

CCAUGGACGAGCCUGUAGUAUACAACGGUAA

>P62

ACAAAGGUCUUCCUAUGAUUCCGGCGUUCGU

>P63

CUUUCUCAUACCCUGUAGACCAGACCUCUCU

>P64

AGAAUACUUUGAAGGUUUAACCGAGGAAAUG

>P65

CGUGGAGACCGGGGUUCGACUCCCCGUAUCG

>P66

UUAUCCGAUAUAGUGUAACGGCUAUCACAUC

>P67

GGACACUUCUGAUGUUUCAAAAGAUAUUCCA

>P68

UAACUGUGGGAAUACUCAGGUAUCGUAAGAU

>P69

GUAAGAUGCAAGAGUUCGAAUCUCUUAGCAA

>P70

ACAAUUUUCACAGUUUAAGGCCAAGAACAAG

>P71

GCCCGUUUACACAUUUUGAUACAACCGUAGA

>P72

CGGGAGGUCCCGGGUUCGAGUCCCGGCUCGC

>P73

GAUUCUCGCUUAGGGUGCGGGAGGUCCCGGG

>P74

GCGUGCGACUGUUAAUCGCAAGAUCGUGAGU

>P75

CGCAAGAUCGUGAGUUCAACCCUCACUGGGG

>P76

CGUUGGGCCCCCGGUUCGAUUCCGGGCUUGC

>P77

UGGUAAAAUCCAACGUUGCCAUCGUUGGGCC

>P78

CUAGCGCAAGUGGUUUAGUGGUAAAAUCCAA

>P79

GGCUGUGUUCUUCUUUCUAAAUUCCCUAUCG

>P80

GGAAAAACCCGUUGCUAGAAGCGCAACUGGU

>P81

GAAAAAAGUUCAGAAUUGCAGAAAAGUGGUG

>P82

AGUGGUUUCCUAGUGUAUCAGCCACUAUCGG

>P83

CAUAAGGUUAGGGGUUCGAGCCCCCUACAGG

>P84

GCAAUCGGUAGCGCGUAUGACUCUUAAUCAU

>P85

AAACAAAAGAAGCUGUUCCAGAGAGCCCAAG

>P86

CCGGACAACCCCGGUUCGAAUCCGGGUAGGA

>P87

CACUUUCGGUUUUGAUCCGGACAACCCCGGU

>P88

GGUUAUCACUUUCGGUUUUGAUCCGGACAAC

>P89

UAGUGGUUAUCACUUUCGGUUUUGAUCCGGA

>P90

UGUUGCCGCUAAGUGUAAGGAAGUCGGUAUC

>P91

CUGGUAUAUUCUAUAUACUCACUUAUUACUU

>P92

UUCUGGUAUAUUCUAUAUACUCACUUAUUAC

>P93

AAUGGCUCUUUUUGUUAUUCGAAAGCUUACA

>P94

UAAAAAGUUCGGCUAUCUCUUGGGCUCUGCC

>P95

UCUGCCCGCGCUGGUUCAAAUCCUGCUGGUG

>P96

CAUGGAUGAUAUUUGUAGUAUGGCGGAAAAC

>P97

GUGGAGAUCAUCGGUUCAAAUCCGAUUGGAA

>P98

AUACUAUUCAGUUUCUCAGAUAUAGGUUGCA

>P99

GCAAUUGGAAAAAUCUAUUAACCCAGAUGAA

>P100

CCAGUGCGUCUACUAUUACUCGGCCAAAUAU

**II. 100 false ψ site containing sequences (negative samples)**

>N1

UCGUAAUUUGAGAUCUCUGCAAAACAAUGCA

>N2

AAACAAUGCACCUCCUGGCAAAAACAUCAAU

>N3

AAACAUCAAUGUCAAUUGUUUGAACGUCAAU

>N4

GAACGUCAAUUCUUGUUCGUUGUCCGCAAGC

>N5

AAUUAAUAUGGCUUGUAAUGGAAACAAGCAA

>N6

AAACAAGCAAGAUCUUCCCAUACCGUUUCCC

>N7

ACCGUUUCCCCUGCAUGUAGAAUGCAACGAU

>N8

AUCAAUGUUUAAUCAUAACAGAUCAAAGAGC

>N9

AUCAAAGAGCAGUGGUACUACAGAUGCGUCA

>N10

AGGUACGCAUAAGCGUGAACCCCGGUCGACG

>N11

CCGGUCGACGAUACAUACAGAGCUGUUACAA

>N12

UAUAGCAAAGGACAGUAGAAACCUGAGUAAU

>N13

CCUGAGUAAUGGAUCUUUGAAUGAUAUUAAC

>N14

UGAUAUUAACGAAAAUGAAGAGCUCCAAAAU

>N15

GCUCCAAAAUUUCCAUAGAAAAAUCAGCGAA

>N16

UUCCCCAAGGAAAAAUAGCGAAACCAGAAAG

>N17

GUUAAUGCGGGAAGAUUACAUUGCCUUGAAA

>N18

UGCCUUGAAACAACCUCCAAGCUUGGGAGAU

>N19

GAGGAGAUCUCGUCGUUUAAGAACCAAGUCA

>N20

AACCAAGUCAUUCGGUAACAAGUUCCAAGAC

>N21

GUUCCAAGACAUUACUGUCGAACCUCAAUCC

>N22

CGUAGGUAAAGUGUAUUUAGUGAGGGAACGC

>N23

CGCGACAAGUGAUCAUCCAUUUAUUGUGACA

>N24

UAUUGUGACACUGUAUCAUUCCUUUCAAACC

>N25

AAACAUAUUACUGCAUCAAUCUGGUCAUGUC

>N26

UGGUCAUGUCAUGCUUUCUGACUUUGAUUUA

>N27

AGAUACAAAAAUUUGUUCAGAUGGAUUCAGA

>N28

UGGAUUCAGAACUAAUUCCUUUGUUGGUACU

>N29

UGGCUGUACUCCAUUUAAAGGAGAUAAUUCA

>N30

CGUCAAAUUUCCACAUGAUAAGGAAGUUUCG

>N31

GGAAGUUUCGAAGAAUUGUAAAGACCUGAUA

>N32

CUUCUUCAAAAAAGUUCAGUGGUCGUUCUUA

>N33

CCCCCCUCUAAUACCUGCAUUAAAUGAUAAC

>N34

UCCUUUUAUAUUGUCUUGCAAUAAACACCCG

>N35

AAACGAUGAUGAAAUUGAUGAGGCUGAUCCA

>N36

GGCUGAUCCAUUCCAUGAUUUUAAUUCUAUG

>N37

CUACUCUGAAAAUUAUACCUACGGAAAAAUU

>N38

AUCUUAUGAUAAAAAUGUAAAAAAUUAUUUA

>N39

AAACGAGAAAGUGAAUGAAAAAUAUAAUAUC

>N40

AUAUAAUAUCAUUUAUUGUCUGAUAAUGCUG

>N41

UACGUACCAUCCGCAUCAGUGGAUAUCCAAU

>N42

GAUAUCCAAUGAUAGUAAUUUCGCGAGUUUA

>N43

CGCGAGUUUAUCCGUUGCUGUUAUAUUAUCA

>N44

UAUAUUAUCACUUUUUAAUAUUCUUUUCAAA

>N45

GGAUUCCUUCCGCAAUUCUUCUGAAAUACUG

>N46

CUCGCCAGUUUUUUGUUCUUCCACGUAAUCC

>N47

CCUUAUUAACGGAGAUUUGAUUUCUCCCAGC

>N48

ACCGAUUCGAGUGAGUACGUUUUCAAAUAUG

>N49

UUCAAAUAUGCUUAAUCUGAUCUCUUCUGCG

>N50

GCCGAUCUGUGCCAUUAUAGUAAGCAGUGCC

>N51

AAGCAGUGCCACUUGUCUAAUAUAAGAUGAU

>N52

GAUUUUACCGUUUUCUGGGGACAUCAUGAUA

>N53

CAUCAUGAUAUCAUUUGGUACAUAAUGAACA

>N54

GAUAAUUGGAUUUCUUGCAUUUUUUGCGAUU

>N55

UUUUGCGAUUAUGGCUUGUUGACCAUUCACA

>N56

ACCAUUCACAAAAGUUGGUCUAACAUAAUUU

>N57

UAAGUCCUUGUAAUAUUCUAGCUUUUGAGUC

>N58

UCUGGGAGUGGUAAAUCUACUGACCAUCUUC

>N59

UUUUAUCCAAUCAUCUGGCAAGUCCUUAAUU

>N60

UUCAUCUCUAAAAUUUAGAUAUGGACGUUUG

>N61

UGGACGUUUGAGAUAUUUUCGUAUUUCUGCC

>N62

UAUUUCUGCCAAUUCUUCCUUUAACUGUGAC

>N63

UAACUGUGACCGUACUGAUUCGCUUUCCCUU

>N64

GCUUUCCCUUUGAAUUUUUAUUAUACCCUCU

>N65

CAUUACUUGCUUAUCUGAAUUUUUUUCCAUU

>N66

UUGUUUGCCUAUCCUUCCAUCUGAUGACUUG

>N67

UGAUGACUUGAAAUGUUCUGACAGGUAAGAU

>N68

UCUCAACAUUCUUAAUCCAAACGAUGUCCUU

>N69

CUCCUGCUUGUGUAUUAAAGGACAUGAAAUA

>N70

UUUCGCUACAUGUAAUGGAAGAUCAUCUGUA

>N71

GAUUCGUAUCUGUAGUUUCUCAUCAGCAAGA

>N72

UCUUUCAAAAACGCUUGAUUUGCUGGCACCU

>N73

CUUAAUAGCGCUUGUUUCUGCUUGCUCUACC

>N74

CUCUAGGUGAACGUUUAAUCUGACAUCCGGG

>N75

AAGUUUGAUGUGAAGUAUUCUGCUAACCGUU

>N76

CAGGUCCUUCACCUGUUUGUCCAAGGGAGUA

>N77

AGGGGACUUUCUGGCUUUUUUUUUUACGAAA

>N78

CUCUUCCUCAUCAUCUUCAGCCUCAACAUUU

>N79

UCCAACCGCAACUUCUUGUUCUUGCUUAUGC

>N80

CCUGCUUAUUGUGGGUUGUCCCGCCAUUAUU

>N81

CGCCAUUAUUGUUAAUAGAUUCAACAAAAUA

>N82

UUUAUCAUUGAAAAUUCACGUGAUCGCAAUA

>N83

GAUCGCAAUAUUCCGUCAGGAGUGAUAAAUA

>N84

UCGUCAUUGCACAAAUUAGUUUAUUAUUCAC

>N85

UAUUAUUCACGACUCUUAACAACGACAAUUU

>N86

UAGACAGGUCGUCCGUAGAUAUUUACAUAAA

>N87

UACUACACAGACUACUAUUAGAAUUUGCGAA

>N88

AAUUUGCGAAGGAUUUACCGAAGAAAAGCAC

>N89

AGAAAAGCACAGACCUUAUUGAGCUUUUGAA

>N90

UCAAUAACCAGGAGUUUCAAAAACAAACAGG

>N91

CACUUUUCAUUGAUCUAUUUGAUAAAUCUGC

>N92

CACUAGAGUCCAAUCUACGCGACUUAUUGCA

>N93

ACUUAUUGCAUUCCUUGGAAGGUGAAAGUCU

>N94

UGCACGAUGGUCCAGUUAUUGAUGAAUUUUU

>N95

AUUUGGCCAUUCAACUUCAUAAGUGGUCGGU

>N96

AAGGUACCAGGAAAGUUUCUGAAACCAUCCC

>N97

AAUCUUUACUCUACUUAUUAUCCAUUGCAUC

>N98

CCCCUGAAUAUCUUAUUUUAGCAUUAGUCAA

>N99

CAUUAGUCAAAGAAAUGAAGCGGUUCGUUUU

>N100

GGUUCGUUUUAUUGAUAGAAAACAGGACAGU
